# Synthesis of polynorbornadienes by ring-opening metathesis polymerization and their saturated derivatives bearing various ester groups and carboxyl groups†

**DOI:** 10.1039/d2ra07779e

**Published:** 2023-01-24

**Authors:** Xiaoxue Lin, Jianjun Shi, Satomi Niwayama

**Affiliations:** a College of Chemistry and Chemical Engineering, Hainan Normal University Haikou Hainan 571158 P. R. China; b Division of Sustainable and Environmental Engineering, Graduate School of Engineering, Muroran Institute of Technology 27-1, Mizumoto-cho Muroran Hokkaido 050-8585 Japan niwayama@mmm.muroran-it.ac.jp

## Abstract

Various symmetric and non-symmetric polynorbornadienes having a variety of ester groups and carboxyl groups were synthesized by ring-opening metathesis polymerization (ROMP) with Grubbs' third generation catalyst (G3 or [Ru]-III catalyst) in a controlled living manner from half-esters prepared by the selective monohydrolysis of symmetric diesters that we previously reported. The half-esters thus obtained can be directly submitted to ROMP with the G3 catalyst, leading to mostly the *trans* structure and narrow polydispersity indexes. The subsequent hydrogenation yielded saturated polymers, improving the thermostabilities according to the *T*^5^_d_ results. Our selective monohydrolysis reactions combined with ROMP initiated by the G3 catalyst have proven to be an efficient tool for the production of a variety of homopolymers with well-controlled structures in a living manner.

## Abstract 2

Polymer libraries were synthesized in a controlled living manner from half-esters efficiently prepared by the selective monohydrolysis of symmetric diesters.

## Graphical abstract

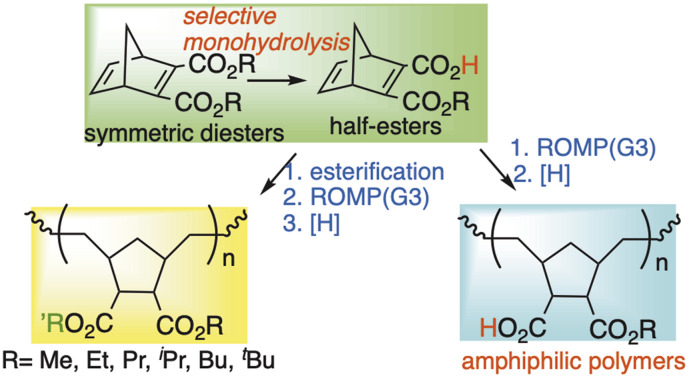

## 1 Introduction

**Scheme 1:**
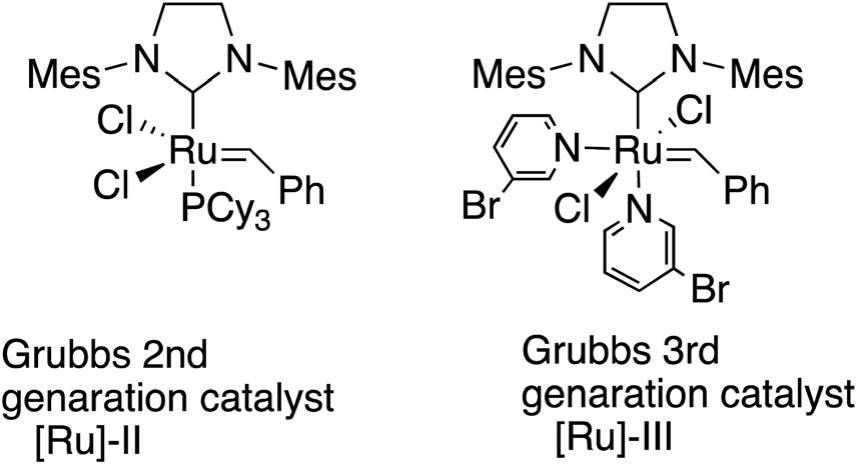
Grubbs 2nd and 3rd generation catalysts.

Polyolefins are among the most widely utilized materials in a variety of research fields. Ring-opening metathesis polymerization (ROMP) is a metal carbene-catalyzed polymerization reaction starting from strained cyclic alkenes, which produce a wide range of polyolefins,^1^ and has been attracting a great deal of attention because of its ability to enable living polymerization and hence a wide range of applicability in polymer materials.^2^ In particular, Chauvin, Grubbs, and Schrock *et al.*^3^ developed ROMP with [Ru]-based catalysts, taking advantage of their general insensitivities to air or moisture and their high tolerance for various functional groups. The driving force for the polymerization is explained to be the strain of the [Ru] catalysts in addition to the strain of alkenes.^4^ Among these catalysts, 2nd generation (G2 or [Ru]-II)^5^ and 3rd generation (G3 or [Ru]-III)^3*f*,6^ are widely utilized because of their stability (Scheme 1). Functionalized polymers bearing polar functional groups^7^ have also been synthesized by the highly reactive G3 catalyst, and have been applied to various materials.^8^

**Scheme 2:**
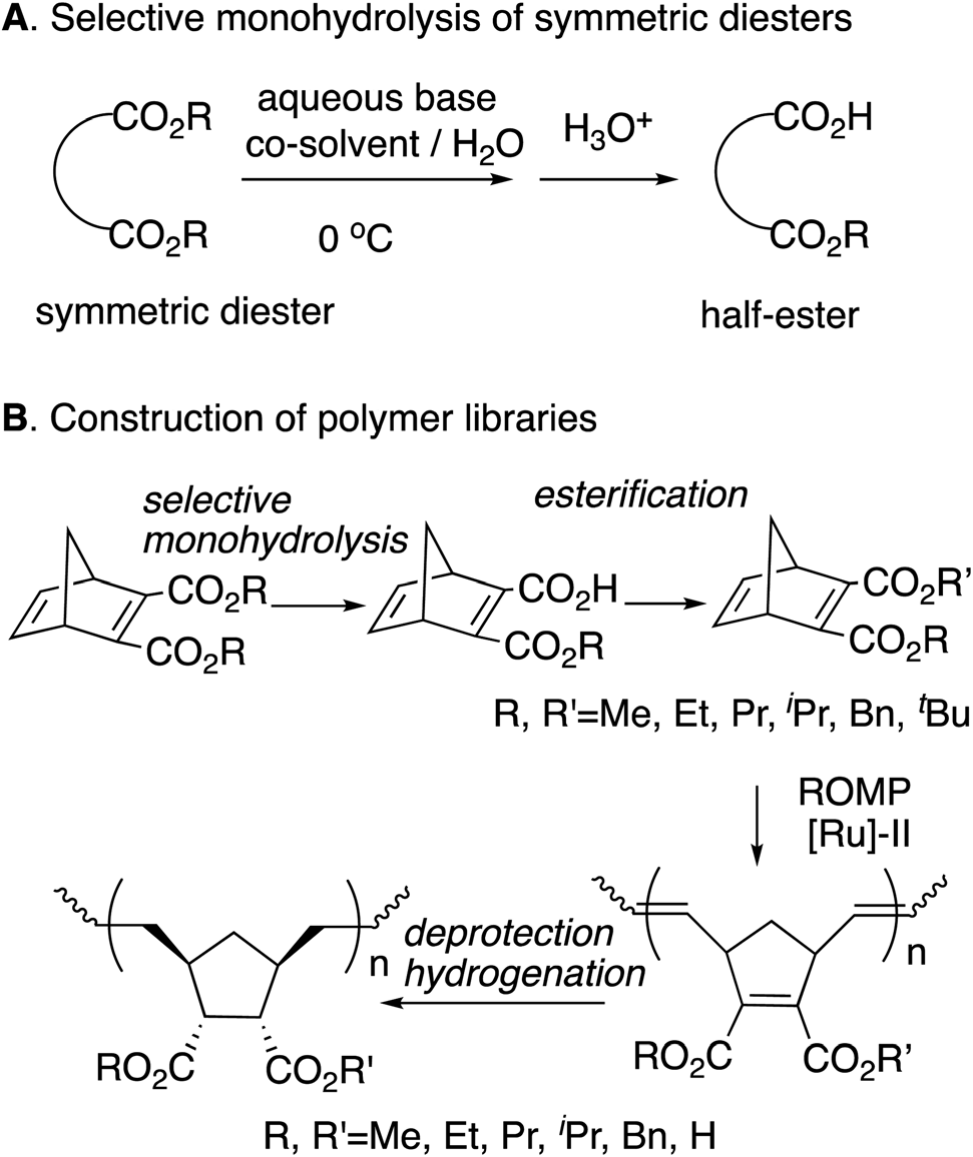
Selective monohydrolysis and the application to polymer synthesis.

We previously reported the synthesis of polymer libraries by ROMP with the Grubbs 2nd generation catalyst using the half-esters obtained by the selective monohydrolysis of symmetric diesters we developed earlier.^9^ This selective monohydrolysis reaction enables hydrolysis of one of the two identical ester groups in various symmetric diesters, producing half-esters in high yields (Scheme 2A).^10^ The symmetric diesters having a norbornadiene skeleton were efficiently monohydrolyzed for the production of the corresponding half-esters, and their various derivatives, bis(alkoxycarbonyl)norbornadienes, were polymerized with the Grubbs 2nd generation catalyst in a well-controlled manner. We therefore demonstrated that this selective monohydrolysis is a powerful tool for the construction of libraries of polymers with a variety of functional groups including amphiphilic polymers having carboxyl groups (Scheme 2B).

However, these polymers exhibited rather broad polydispersity indexes ranging from around 1.2 to 1.9. The molecular weights of the obtained polymers also appeared rather uncontrolled in some cases because of the slower reaction rates in the initiation step and faster propagation rates.^11,12^ The [Ru]-II catalyst also appears to be less tolerant of a polar functional group like COOH. Since the Grubbs 3rd generation catalyst is known to enhance the reactivities in the initiation steps of the polymerization by the 3-bromopyridine ligand, resulting in improved living polymerization, here we report a more precise and controlled living ROMP method for the construction of libraries of polymers with the Grubbs 3rd generation catalyst from symmetric and non-symmetric diesters bearing the norbornadiene skeleton that were obtained by our selective monohydrolysis reactions. We also found that the amphiphilic homopolymers can be synthesized directly from the half-esters obtained by the selective monohydrolysis reaction because of the higher tolerance of the catalyst.

## 2 Results and discussion

### 2.1 Polymerization, characterization, and thermal properties of norbornadienes bearing various ester groups

We first studied the ROMP of six symmetric diesters 1–6 to examine the influence of the [M]/[I] ratios with the [Ru]-III catalyst at −15 °C. The results of the isolated yields, number average molecular weight (*M*_n_), the *trans* contents around the C=C bonds in the main chain of polymers, the thermogravimetric analysis (TGA) results, and the differential scanning calorimetry (DSC) of polymers 1a–6a are shown in Table 1.

**Table 1:** ROMP of symmetric norbornadiene diesters and the characterization of the polynorbornadienes

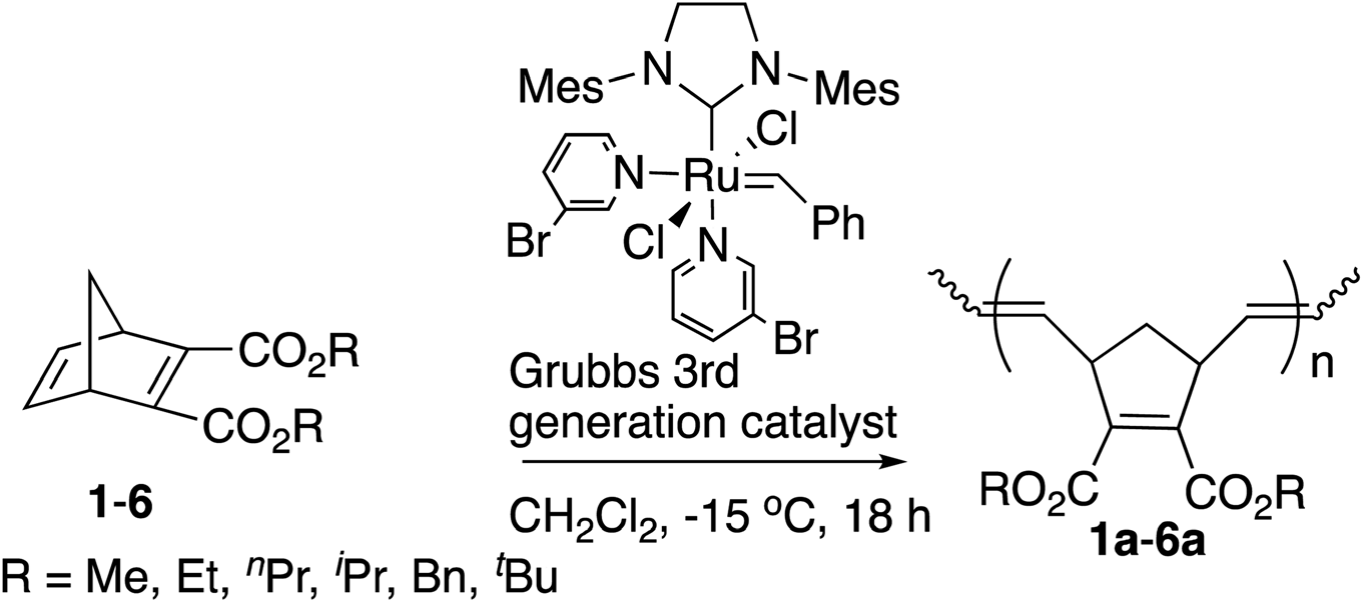										
Entry	Diester	R	[M]/[I] (mole ratio)	Yield^a^ (%)	*M*_calcd_ b (kDa)	*M*_n_ (GPC)^c^ (kDa)	PDIs^d^	*Trans* content^e^ (%)	*T*_g_ (°C)	*T* ^5^_d_ f (°C)
1	1	Me	333	—g		—g	—g	—g	—g	—g
2	1	Me	200	82	3.4	7.0	1.05	>99	114	229
3	1	Me	100	72	1.5	1.3	1.09	>99	77	205
4	2	Et	200	76	3.6	6.7	1.08	>99	105	231
5	3	^*n*^Pr	200	71	3.7	5.7	1.06	>99	92	232
6	4	^i^Pr	200	72	3.8	5.2	1.04	95	99	184
7	5	^*n*^Bu	200	67	3.9	3.7	1.05	>99	84	217
8	6	^*t*^Bu	200	64	3.7	2.1	1.10	90	117	125

a Isolated yield after precipitation.

b Calculated theoretical molecular weight obtained by a formula: *W*_monomer_ × [M]/[I] × yield%, where *W*_monomer_ is the molecular weight of monomer.

c Determined by GPC with polystyrene standards.

d Values of *M*_w_/*M*_n_ determined by GPC.

e Determined by ^1^H NMR or ^13^C NMR.

f Temperature at 5% weight loss.

g Solid polymer did not form after reprecipitation.

Solid polymer 1a was not obtained under the common [M]/[I] ratio of 333 ^13^ after the quench and evaporation of the solvent (entry 1, Table 1). Extending the reaction time from 18 to 48 hours and raising the polymerization temperature from −15 °C to room temperature did not help the polymerization either. However, when the amount of [Ru]-III catalyst was increased ([M]/[I] ratio = 200), the yield improved to 82% and a rather narrow polydispersity index of 1.05 was observed at −15 °C after 18 hours (entry 2, Table 1), indicating that corresponding polymer 1a was produced in a controlled living manner as has been reported by others.^3*f*,14^ Therefore, this molar ratio (200) was adopted for the polymerization of the remaining monomers in Table 1.

The yields tended to decrease with the increased size of the alkyl groups. This tendency is consistent with the previous studies by us^9^ and others.^13,15^ When the reaction time took more than 18 hours, the *M*_n_ values and the PDIs increased with the reaction time (data not shown). Polymer 2a was obtained with a high yield (76%) and narrow PDI (1.08) at −15 °C under the same reaction conditions as 1a (Table 1, entry 4), which is better than the reported ROMP of 1 and 2 with the Ru-based catalysts^16^ and the Mo-based catalysts^17^ (1.2–1.8 of PDI values). The comparison also demonstrates that the polymerization was initiated by the [Ru]-III catalyst rapidly even at −15 °C, suppressing the chain-transfer reactions despite the increased steric hindrance and possible intramolecular/intermolecular coordination of ester groups. Moreover, more sterically hindered monomers 3–6 showed high reactivities of ROMP in a living manner with narrow PDIs of 1.04–1.10 (Table 1, entries 5–8). The observed *M*_n_ values of 2a–6a showed better matches with the calculated theoretical values than polymer 1a, indicating that the controlled *M*_n_ was within a reasonable scope under the [M]/[I] ratio of 200. The fact that the observed *M*_n_ values show somewhat broader ranges than the theoretical values was perhaps derived from the imbalanced rates between the initiation and the propagation.

Polymers 1a–6a from symmetric diesters with the norbornadiene backbone often favor the “*trans*” configuration in ROMP initiated by Ru-based catalysts, and NMR spectroscopic studies have also been reported for this stereochemistry.^16*a*,18^ Generally, it is perceived that the “*trans*” isomers are more thermally stable, but the *trans* content of polymers 1a–6a decreased slightly with the increase in the size of side groups, perhaps due to the increased steric hindrance of the ester functional groups in the process of forming metallacylobutane intermediates.^13,15,16^

Polymers 1a and 2a obtained here revealed the presence of one sharp doublet at near 5.40 ppm, suggesting the all-trans structures. Polymers 4a–6a, with an increased size in the alkyl substituent, slightly lacked solid microstructures, reducing the *trans* ratios for the junctions between two adjacent cyclopentene units. The *trans* content (%) was determined from the ^1^H NMR or ^13^C NMR spectra as reported in the literature.^16^

The glass transition temperature (*T*_g_) and the 5% decomposition temperature (*T*^5^_d_) exhibited a broad range, 77–117 °C and 125–232 °C, respectively. Various *T*_g_ values are necessary for practical applications for the synthesis of libraries of polynorbornadienes. These polymers from symmetric diesters displayed good thermal stabilities, and the crystalline melting point was not detected. In general, the *T*_g_ decreased with the extended size of alkyl pendant chains and increased with the branching of alkyl groups. The *T*_g_ values of polymers 4a (R = ^i^Pr, 99 °C) and 6a (R = ^*t*^Bu, 117 °C) bearing the branched alkyl pendant were higher than the corresponding polymers with a linear structure, polymer 3a (R = ^*n*^Pr, 92 °C) and 5a (R = ^*n*^Bu, 84 °C). This effect can be explained by the plasticizing effect of the hydrocarbon chain and the softer side groups of the flexible longer alkyl pendant.^13,15^ The *T*^5^_d_ of polymer 6a was found to have the lowest temperature, 125 °C, resulting from the facile decomposition of *tert*-Bu substituted polymers due to the release of the isobutene molecule.

Next various non-symmetric diesters, 7–11, were prepared from the half-esters obtained by the highly efficient selective monohydrolysis of symmetric diesters 1–6, and were polymerized by the [Ru]-III catalyst under the same conditions as above. The results are summarized in Table 2. The *tert*-Bu group was introduced into the COOH of the half-esters with the use of magnesium chloride in high yields.^19^ As these diesters were racemic mixtures, the *tert*-butyl and alkyl ester groups were interchangeable in these structures.

**Table 2:** ROMP of non-symmetric norbornadiene diesters and the characterization of the polynorbornadienes

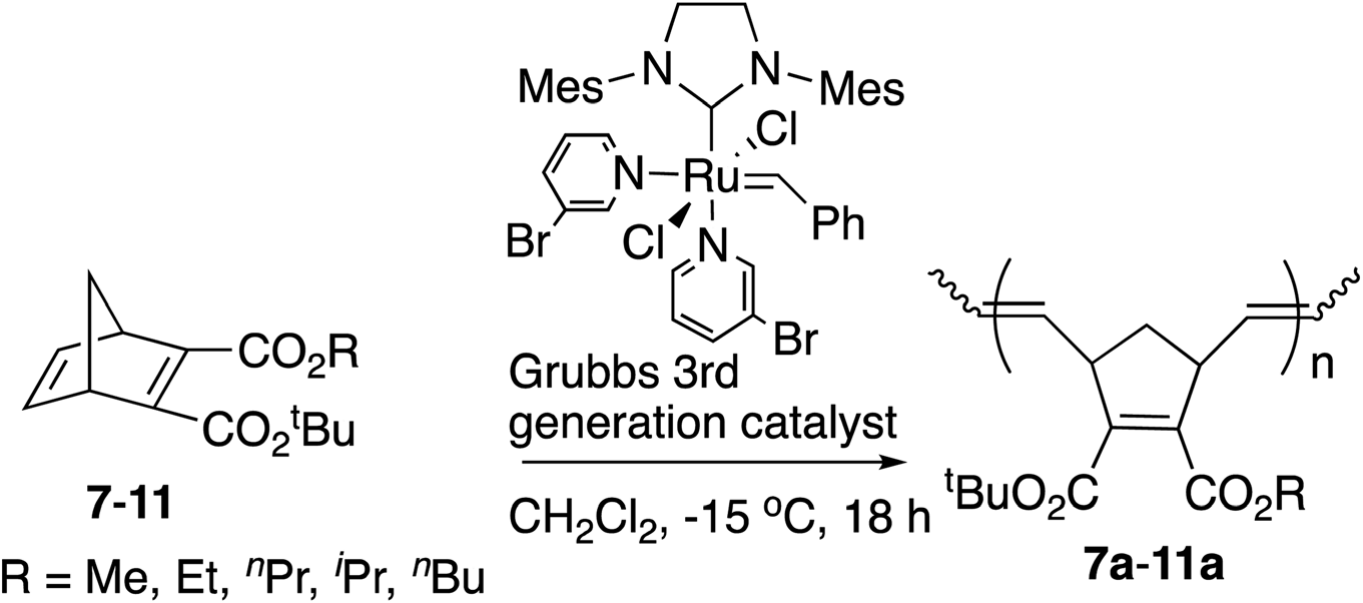									
Entry	Diester	R	Yield^a^ (%)	*M*_calcd_ (kDa)	*M*_n_ (GPC) (kDa)	*M*_w_/*M*_n_^b^	*Trans* content^c^ (%)	*T*_g_ (°C)	*T* ^5^_d_ d (°C)
1	7	Me	68	3.6	7.11	1.08	53	121	231
2	8	Et	62	3.9	5.96	1.07	51	114	235
3	9	^*n*^Pr	58	4.0	5.07	1.05	47	107	236
4	10	^i^Pr	57	4.1	4.89	1.09	45	109	182
5	11	^*n*^Bu	49	4.3	2.73	1.09	45	96	162

a Isolated yield after precipitation.

b Values of *M*_w_/*M*_n_ determined by GPC with polystyrene standards.

c Determined by ^1^H NMR.

d Temperature at 5% weight loss.

Decreased yields of polymers 7a–11a with an increase in the size of alkyl groups in ester substituents were also observed. The yields of these polymers from non-symmetric monomers were lower than those of polymers from symmetric monomers due to the steric hindrance of *tert*-Bu ester groups. The *M*_n_ observed by GPC for polymer 7a, which is similar to polymer 1a, was somewhat higher than theoretical values. The correlations between the calculated theoretical *M*_n_ (ranging from 3.6–4.3 kDa for polymers 8a–11a) and the observed *M*_n_ values from GPC (ranging from 2.73–5.96 kDa for polymers 8a–11a) were better than for the symmetric polymers, 1a–6a. Despite the less-than-ideal yields, the narrow PDIs (1.05–1.09) of the resulting polymers implies living polymerization. The structure of the non-symmetric polymer unit was determined by the ^1^H NMR and ^13^C NMR spectra. The *trans*/*cis* ratios were near 1 : 1 according to the integral ratios of olefinic protons in the ^1^H NMR spectra, without showing the diastereoselectivities. One probable reason is that the possibilities for the coordination of the active center of metal carbene at the position of *exo*-face or *endo*-face in the ring-opening step are equal. The other probable reason is the steric hindrance or the increased stability of the *cis* configuration. Again an increase in *T*_g_ values was observed with branched alkyl groups, and a decrease in *T*_g_ values was observed with longer alkyl chains.

### 2.2 Synthesis of amphiphilic polymers with half-esters by Grubbs' 3rd generation catalyst

**Scheme 3:**
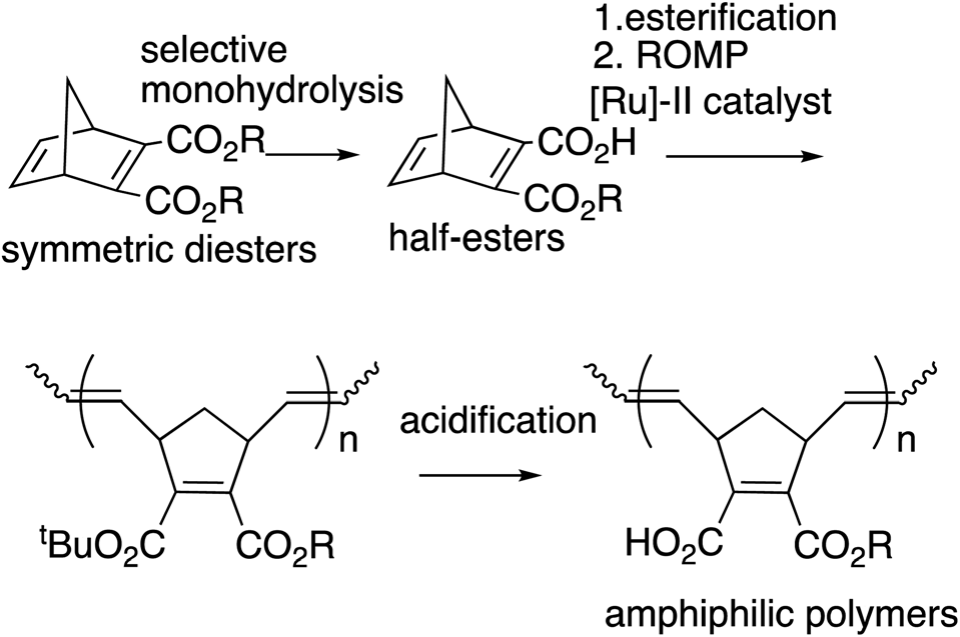
Synthetic route of amphiphilic polymers with the [Ru]-II catalyst.

In our previous studies,^9^ the direct polymerization of half-ester monomers by the [Ru]-II catalyst led to the production of only a trace amount of polymers even after more than 3 days due to the catalyst sensitivity with the carboxyl group. Therefore, the –COOH was protected as the *tert*-Bu ester before ROMP and was deprotected to the –COOH group successfully with trifluoroacetic acid (TFA) afterwards as shown in Scheme 3.^10^

However, half-esters 12–16 were able to be polymerized by the [Ru]-III catalyst directly because of the catalyst tolerance for carboxylic groups as shown in Table 3. The polymerization was conducted in anhydrous THF because of the low solubility of the obtained polymers in CH_2_Cl_2_.

**Table 3:** Synthesis of amphiphilic polymers with half-esters and characterization of the polymers

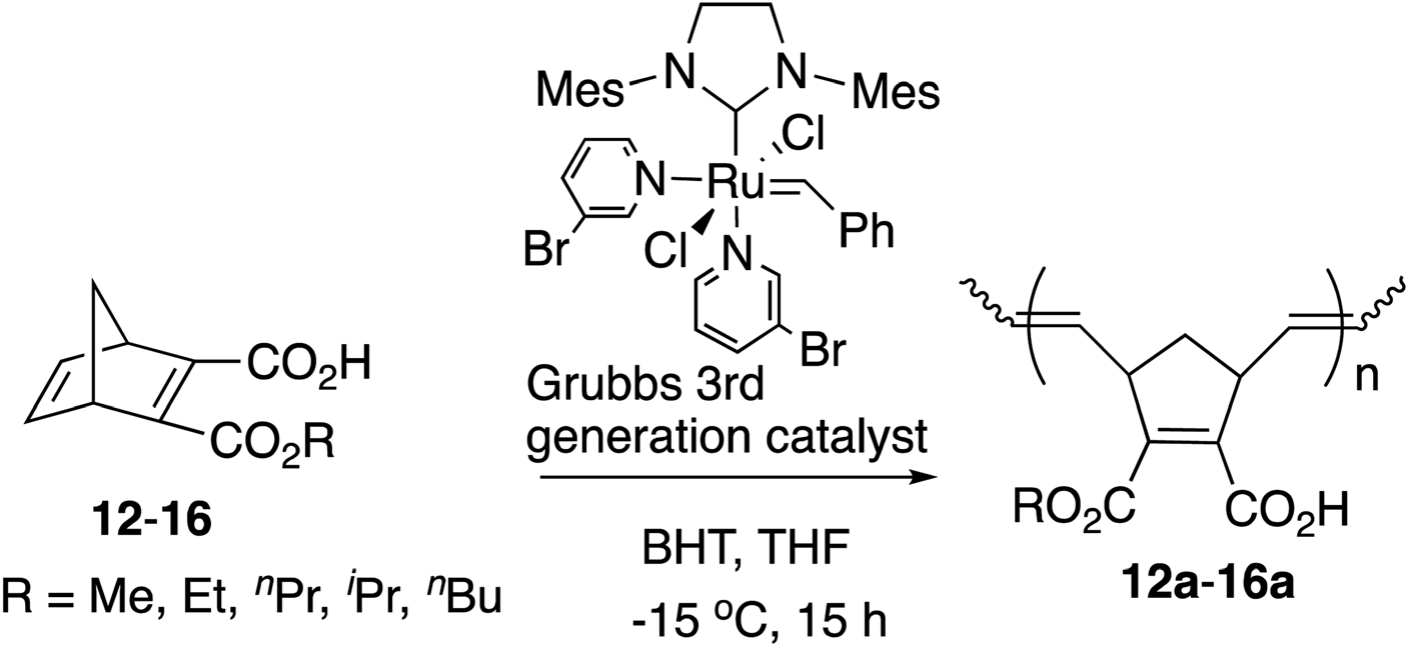										
Entry	Half-ester	R	[M]/[I] (mole ratio)	Yield^a^ (%)	*M*_calcd_ (kDa)	*M*_n_ (GPC) (kDa)	*M*_w_/*M*_n_^b^	*Trans* content^c^ (%)	*T*_g_ (°C)	*T* ^5^_d_ d (°C)
1	12	Me	100	58.3	1.13	1.93	1.13	>99	73	175
2	12	Me	50	77.2	0.75	2.64	1.24	>99	76	184
3	13	Et	100	32.6	0.68	1.85	1.16	>99	72	154
4	13	Et	50	70.9	0.74	2.57	1.27	>99	74	201
5	14	^*n*^Pr	100	18.1	0.40	0.92	1.20	99	67	179
6	14	^*n*^Pr	50	61.4	0.68	1.91	1.29	>99	69	208
7	15	^i^Pr	100	14.5	0.32	0.66	1.18	>99	69	175
8	15	^i^Pr	50	62.5	0.69	2.21	1.23	98	70	183
9	16	^*n*^Bu	100	—e	—e	—e	—e	—e	—e	—e
10	16	^*n*^Bu	50	37.4	0.42	0.75	1.22	98	68	177

a Isolated yield after precipitation.

b Values of *M*_w_/*M*_n_ determined by GPC with polystyrene standards.

c Determined by ^1^H NMR.

d Temperature at 5% weight loss.

e Solid polymer did not form after reprecipitation.

The [M]/[I] ratio of 200, which was effective in the ROMP of 1–11, yielded only a low (∼27%) yield in the ROMP of 12 (data not shown). However, an increased catalyst ratio of 50 led to the production of the corresponding amphiphilic homopolymers. The yields obviously decreased with the increase in the size of the alkyl groups. It appears that addition of a radical scavenger, 2,6-di-*tert*-butyl-4-methylphenol (BHT), helped improve the polymerization as has been reported for polymerization of other carboxyl group-containing monomers.^7*a*^ Although the PDIs of these amphiphilic polymers showed a marked increase compared to the polymers from the diester monomers above, these values remained within a favorable range (1.13–1.29). The observed *M*_n_ values from GPC (0.75–2.64 kDa) were comparable to the theoretical *M*_n_ values (0.42–0.75 kDa). The higher molecular weights also indicate good polymerization control and a “living” manner. The olefinic protons in the monomers at around 6.40 ppm disappeared, and new olefinic protons were found at 5.63–5.37 ppm, indicating the *trans* and *cis* structures around the main chain in the polymers. Other significant differences in the polymers above from the non-symmetric monomers include their high selectivities towards the *trans* structures, probably due to the mutual interaction of carboxylic groups in the polymer chains. Judging from the results of *T*_g_ and *T*^5^_d_, their thermal stabilities decreased slightly compared to polymers 1a–11a above with the same ester groups, perhaps due to the decreased *M*_n_ values.

### 2.3 Synthesis of saturated polynorbornadienes by hydrogenation

Next we performed hydrogenation of the polymers above in order to obtain saturated polymers, which is expected to improve thermal properties as well as to provide useful information for structural elucidation.^16*b*^ The hydrogenations were performed by the rapid *in situ* addition of diimide (NH=NH) with the use of *p*-toluenesulfonyl hydrazide (TSH) and 2,6-di-*tert*-butyl-4-methylphenol in xylene at 135 °C. The results are summarized in Table 4.

**Table 4:** The synthesis of polymers with a saturated backbone by hydrogenation and characterization of the polymers

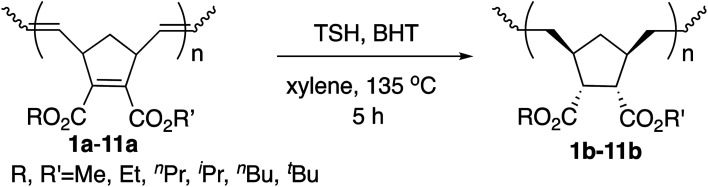								
Entry	Polymer	R	R′	Yield^a^ (%)	*M*_n_(GPC) (kDa)	*M*_w_/*M*_n_^b^	*T*_g_ (°C)	*T* ^5^_d_ c (°C)
1	1a	Me	Me	99.4	6.65	1.04	72	233
2	2a	Et	Et	97.8	7.13	1.07	66	230
3	3a	^*n*^Pr	^*n*^P	96.6	5.72	1.07	58	242
4	4a	^i^Pr	^i^P	96.3	5.34	1.09	69	195
5	5a	^*n*^Bu	^*n*^Bu	95.5	3.96	1.08	50	221
6	6a	^*t*^Bu	^*t*^Bu	94.4	2.11	1.07	74	129
7	7a	Me	^*t*^Bu	90.4	6.77	1.07	89	243
8	8a	Et	^*t*^Bu	86.1	6.25	1.08	75	237
9	9a	^*n*^Pr	^*t*^Bu	83.4	4.94	1.08	62	239
10	10a	^i^Pr	^*t*^Bu	79.6	5.12	1.09	65	190
11	11a	^*n*^Bu	^*t*^Bu	72.7	2.93	1.10	48	174

a Isolated yield after precipitation.

b Values of *M*_w_/*M*_n_ determined by GPC with polystyrene standards.

c Temperature at 5% weight loss.

The yields were high, and the PDIs remained similarly narrow compared to those of the unsaturated polymers, ranging from 1.04 to 1.10. The isolated yields also similarly decreased with the increase in the size of the ester groups. The proton signals of the C=C bond in the olefinic region disappeared, and both the C=C bonds in the main chains and the cyclopentene ring were completely hydrogenated without side reactions according to ^1^H NMR and ^13^C NMR analysis as in our previous studies with the [Ru]-II initiated ROMP.^9^ Due to the concerted pericyclic mechanism, the addition mode of hydrogen atoms is known to be specifically “*syn*,” and the addition occurred from the *exo* face of the cyclopentene ring to afford thermodynamically stable and less hindered products.^16*b*,20^ There was no splitting of signal peaks as investigated in ^13^C NMR spectroscopy after hydrogenation, which also suggests the high tacticity of the polymers. The carbon atoms of C_5,6_ in cyclopentane associated with ester groups showed sharp singlets, which indicates that the stereoselective *cis*-addition of two hydrogen atoms occurred on the endocyclic C=C bond and without introducing new tacticity splitting. Although the *T*_g_ values decreased after hydrogenation because of the increased mobilities of the polymer chains, the thermostabilities were indeed improved, based on the *T*^5^_d_ values.

Hydrogenation was also performed on the amphiphilic polymers 12a–16a under the same reaction conditions, and the results are shown in Table 5. The hydrogenation was complete on both the polymer main chain and cyclopentane rings judging from the ^1^H NMR analysis.

**Table 5:** Synthesis of polymers with saturated backbone by hydrogenation and characterization of the polymers

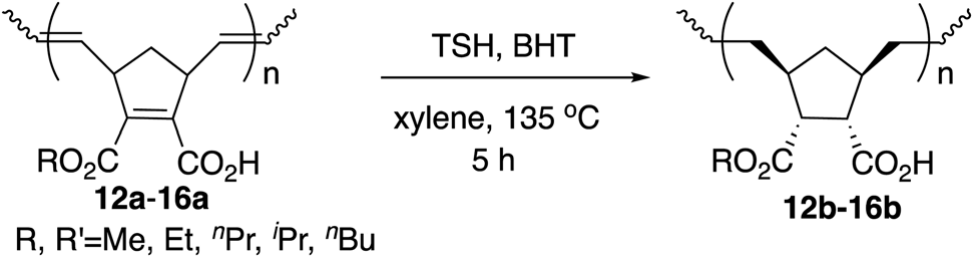							
Entry	Polymer	R	Yield^a^ (%)	*M*_n_(GPC) (kDa)	*M*_w_/*M*_n_ b	*T*_g_ (°C)	*T* ^5^_d_ c (°C)
1	12a	Me	93.1	2.67	1.26	73	185
2	13a	Et	89.2	2.58	1.28	62	211
3	14a	^*n*^Pr	74.8	2.06	1.28	54	213
4	15a	^i^Pr	76.5	2.35	1.29	55	186
5	16a	^*n*^Bu	61.3	0.98	1.27	48	182

a Isolated yield after precipitation.

b Values of *M*_w_/*M*_n_ determined by GPC with polystyrene standards.

c Temperature at 5% weight loss.

Again, high yields and narrow PDIs were observed. Compared with the results in Table 3, the thermal properties were indeed improved for all the cases judging from the values of *T*^5^_d_, although *T*_g_ values decreased as above. Although it is generally known that undesirable side reactions often occur with polymers, these hydrogenation reactions took place smoothly and completely, which is remarkable as in our previous studies.^9^

## 3 Conclusions

We have constructed libraries of polymers by ROMP initiated by the [Ru]-III catalyst from half-esters prepared by the selective monohydrolysis of various symmetric diesters. The polymers from symmetric diesters were obtained with mainly the *trans* olefinic microstructure in high yields. The *T*_g_ values decreased with the increased size of the ester groups, and the *T*_g_ values increased with the branched alkyl groups. The polymers from non-symmetric monomers did not exhibit selectivities toward the *trans* structures. In addition, various amphiphilic homopolymers bearing both ester groups and carboxyl groups were directly prepared from the half-esters by the [Ru]-III catalyzed ROMP. These amphiphilic homopolymers also exhibited high *trans* ratios for the olefinic stereochemistry and thermal properties similar to other polymers. Their thermostabilities were improved by the hydrogenation judging from the *T*^5^_d_ results.

Amphiphilic polymers generally constitute a wide range of advanced materials applied to tissue engineering, drug delivery, surface modifications, *etc.*, and many amphiphilic block copolymers consisting of hydrophobic and hydrophilic monomers have been reported by tandem ROMP or ATRP, often combined with protection and deprotection of polar functional groups. Although these copolymers can have well-defined structures, the composition distribution is not necessarily uniform, leading to low thermal stabilities. Therefore, the direct synthesis of amphiphilic homopolymers from amphiphilic monomers such as half-esters makes a significant contribution to materials development. Our selective monohydrolysis reactions coupled with ROMP initiated by the [Ru]-III catalyst serve as a versatile tool for the production of a variety of amphiphilic homopolymers with well-controlled structures in a living manner.

## 4 Experiment

### 4.1 General

All the reactions were carried out in a nitrogen atmosphere. The glassware was dried in an oven (180 °C) and heated under reduced pressure before use.

Nuclear magnetic resonance (^1^H NMR and ^13^C NMR) spectra were characterized on a Bruker AV 600 mHz NMR spectrometer with chloroform-d as a solvent; chemical shifts (in ppm) were reported using trimethylsilane as the internal standard. The gel permeation chromatography (GPC) was performed at 30 °C in CHCl_3_ (0.85 mL min^−1^) with the use of a JASCO PU-2080 system equipped with a set of Shodex K-804 and Shodex K-805 columns. The number average molecular weights (*M*_n_), weight average molecular weights (*M*_w_), and polydispersity indexes (PDI) of the obtained polymers were calculated on the basis of a polystyrene calibration. For polymers 12a–16a and 12b–16b, the GPC was performed on the methylated samples with trimethylsilyl diazomethane at 40 °C in THF (1.0 mL min^−1^) with the use of a Waters 150C and a Waters 410 refractive index detector. TGA analyses were carried out on a Shimadzu TGA-50 instrument in a nitrogen atmosphere (flow rate of 50 mL min^−1^) for the determination of 5% weight decomposition temperatures (*T*^5^_d_) at which 5% weight loss was observed (heating rate of 10 °C min^−1^). DSC analyses were carried out with a Shimadzu DSC-60 instrument in a nitrogen atmosphere (flow rate of 50 mL min^−1^) with liquid N_2_ as a refrigerant for the determination of the glass transition temperature (*T*_g_) (heating rate of 10 °C min^−1^).

### 4.2 Materials

The third generation Grubbs' catalyst was purchased from Sigma Aldrich and used without further purification. Functionalized norbornenes 1–16 were synthesized according to our procedures reported in the literature.^9,10^

### 4.3 Synthesis of polymers

#### 4.3.1 Typical procedures for the synthesis of homogeneous polymers with functionalized ester groups

Unless otherwise specified in the tables, the monomer (0.24 mmol) was dissolved in anhydrous dichloromethane (0.75 mL). The stoichiometric Grubbs' third generation initiator (1.2 × 10^−3^ mmol, 0.5 mol%) was added to the monomer solution in a nitrogen atmosphere at −15 °C. The reaction was carried out for 15 h at −15 °C under strict exclusion of oxygen, and quenched with ethyl vinyl ether (1.4 × 10^−2^ mL, 0.14 mmol). The reaction mixture was stirred for 10 min at −15 °C. The polymer solid was obtained after reprecipitation from dichloromethane (1 mL)/cold hexane (100 mL) with vigorous stirring three times. The yielded polymer was filtrated and dried under reduced pressure at room temperature for 12 h, producing an off-white solid, which was used for the further characterization.

#### 4.3.2 Polymer 1a

Pale white solid. ^1^H NMR (500 MHz, CDCl_3_) *δ* = 5.42 (d, 2H, =CH), 3.93 (br, 2H, =CHC*H*), 3.73 (s, 6H, OCH_3_), 2.53 and 1.45 (m, 2H, CHC*H*_2_CH); ^13^C NMR (125 MHz, CDCl_3_) *δ* = 38.89 (–*C*H_2_–), 44.38 (C=C–*C*H), 52.21 (CH_3_), 131.60 (C=C), 142.42 (*C*=*C*–C=O), 165.40 (C=O).

#### 4.3.3 Polymer 2a

Pale white solid. ^1^H NMR (500 MHz, CDCl_3_) *δ* = 5.43 (br, 2H, =CH), 4.18 (q, 4H, OC*H*_2_–CH_3_), 3.94 (br, 2H, =CHC*H*), 2.53 and 1.45 (m, 2H, CHC*H*_2_CH), 1.26 (t, 6H, OCH_2_C*H*_3_); ^13^C NMR (125 MHz, CDCl_3_) *δ* = 14.20 (CH_3_), 38.94 (–*C*H_2_–), 44.46 (C=C–*C*H), 61.09 (OCH_2_), 131.60 (C=C), 142.17 (*C*=*C*–C=O), 164.95 (C=O).

#### 4.3.4 Polymer 3a

Pale white solid. ^1^H NMR (500 MHz, CDCl_3_) *δ* = 5.56 and 5.30 (br, 2H, *cis* and *trans* =CH), 4.10 (m, 4H, OC*H*_2_–CH_2_), 3.93 (d, 2H, =CHC*H*), 2.48 and 1.46 (m, 2H, CHC*H*_2_CH), 1.64 (m, 4H, –CH_2_C*H*_2_CH_3_), 0.93 (t, 6H, CH_3_); ^13^C NMR (125 MHz, CDCl_3_) *δ* = 10.56 (CH_3_), 21.92 (–*C*H_2_–CH_3_), 38.93 (–*C*H_2_–), 44.48 (C=C–*C*H), 66.73 (OCH_2_), 131.61 (C=C), 142.09 (*C*=*C*–C=O), 165.00 (C=O).

#### 4.3.5 Polymer 4a

Pale white solid. ^1^H NMR (500 MHz, CDCl_3_) *δ* = 5.54 and 5.45 (br, 2H, *cis* and *trans* =CH), 5.06 (m, 2H, OC*H*(CH_3_)_2_), 3.92 (d, 2H, =CHC*H*), 2.51 and 1.45 (m, 2H, CHC*H*_2_CH), 1.24 (d, 12H, OCH(C*H*_3_)_2_); ^13^C NMR (125 MHz, CDCl_3_) *δ* = 21.95 (CH_3_), 39.21 (–*C*H_2_–), 44.55 (C=C–*C*H), 68.80 (OCH(CH_3_)_2_), 131.61 (C=C), 142.05 (*C*=*C*–C=O), 164.48 (C=O).

#### 4.3.6 Polymer 5a

Pale white solid. ^1^H NMR (500 MHz, CDCl_3_) *δ* = 5.57 and 5.42 (br, 2H, *cis* and *trans* =CH), 4.11 (m, 4H, OC*H*_2_–CH_2_), 3.95 (d, 2H, =CHC*H*), 2.49 and 1.43 (m, 2H, CHC*H*_2_CH), 1.60 (m, 4H, –OCH_2_C*H*_2_CH_2_), 1.35 (m, 4H, –OCH_2_CH_2_C*H*_2_), 0.91 (t, 6H, CH_3_); ^13^C NMR (125 MHz, CDCl_3_) *δ* = 13.82 (CH_3_), 19.21 (–*C*H_2_–CH_3_), 30.57 (–CH_2_–*C*H_2_–CH_3_), 38.86 (–*C*H_2_–), 44.48 (C=C–*C*H), 64.99 (OCH_2_), 131.57 (C=C), 142.02 (*C*=*C*–C=O), 164.96 (C=O).

#### 4.3.7 Polymer 6a

Pale white solid. ^1^H NMR (500 MHz, CDCl_3_) *δ* = 5.54 and 5.50 (br, 2H, *cis* and *trans* =CH), 3.84 (d, 2H, =CHC*H*), 2.50 and 1.48 (m, 2H, CHC*H*_2_CH), 1.45 (s, 18H, OC(C*H*_3_)_3_); ^13^C NMR (125 MHz, CDCl_3_) *δ* = 28.34 (C(*C*H_3_)_3_), 39.75 (–*C*H_2_–), 44.65 (C=C–*C*H), 81.79 (*C*(CH_3_)_2_), 131.71 (C=C), 142.25 (*C*=*C*–C=O), 164.35 (C=O).

#### 4.3.8 Polymer 7a

Pale white solid. ^1^H NMR (500 MHz, CDCl_3_) *δ* = 5.49 and 5.38 (br, 2H, *trans* and *cis* =CH), 3.90 (s, 3H, O–C*H*_3_), 3.73 (d, 2H, =CHC*H*), 2.49 and 1.58 (m, 2H, CHC*H*_2_CH), 1.45 (s, 9H, –C(CH_3_)_3_); ^13^C NMR (125 MHz, CDCl_3_) *δ* = 28.12 (–*C*H_3_), 39.20 (–*C*H_2_–), 44.28, 44.53 (C=C–*C*H), 51.89 (O–*C*H_3_), 81.91 (–*C*(CH_3_)_3_), 131.36, 131.79 (C–*C*=*C*–C), 141.23, 143.34 (*C*=*C*–C=O), 163.93, 165.64 (C=O).

#### 4.3.9 Polymer 8a

Pale white solid. ^1^H NMR (500 MHz, CDCl_3_) *δ* = 5.50 and 5.42 (br, 2H, *trans* and *cis* =CH), 4.20 (m, 2H, O–C*H*_2_–) 3.89 (m, 1H, =CHC*H*), 3.56 (d, 1H, =CHC*H*), 2.49 and 1.56 (m, 2H, CHC*H*_2_CH), 1.44 (s, 9H, –C(CH_3_)_3_), 1.28 (3H, t, –CH_3_); ^13^C NMR (125 MHz, CDCl_3_) *δ* = 14.40 (–CH_3_), 28.29 (–C(*C*H_3_)_3_), 39.33 (–*C*H_2_–), 44.44, 44.64 (C=C–*C*H), 61.07 (O–*C*H_2_–), 81.96 (–*C*(CH_3_)_3_), 131.57, 131.78 (C–*C*=*C*–C), 141.92, 142.42 (*C*=*C*–C=O), 163.97, 165.50 (C=O).

#### 4.3.10 Polymer 9a

Pale white solid. ^1^H NMR (500 MHz, CDCl_3_) *δ* = 5.50 and 5.42 (br, 2H, *trans* and *cis* =CH), 4.11 (m, 2H, O–C*H*_2_) 3.90 (m, 2H, =CHC*H*), 2.50 (s, 1H, CHC*H*_2_CH), 1.65 (m, 3H, CHC*H*_2_CH and –C*H*_2_CH_3_), 1.44 (s, 9H, –C(CH_3_)_3_), 0.91 (t, 3H, –CH_3_); ^13^C NMR (125 MHz, CDCl_3_) *δ* = 10.51 (–CH_3_), 21.91 (–*C*H_2_CH_3_), 28.11 (–C(*C*H_3_)_3_), 39.19 (=C–CH–*C*H_2_–CH–C=), 44.21, 44.47 (C=C–*C*H), 66.60 (–*C*H_2_–O_2_C), 81.82 (–*C*(CH_3_)_3_), 131.20, 131.63 (C–*C*=*C*–C), 142.03, 142.30 (*C*=*C*–C=O), 163.81, 165.38 (C=O).

#### 4.3.11 Polymer 10a

Pale white solid. ^1^H NMR (500 MHz, CDCl_3_) *δ* = 5.49 and 5.45 (br, 2H, *trans* and *cis* =CH), 5.04 (m, 1H, C*H*(CH_3_)_2_) 3.87 (s, 2H, =CHC*H*), 2.48, 1.70 (m, 2H, CHC*H*_2_CH), 1.42 (s, 9H, –C(CH_3_)_3_), 1.22 (d, 6H, CH(C*H*_3_)_2_); ^13^C NMR (125 MHz, CDCl_3_) *δ* = 21.94 (CH(*C*H_3_)_2_), 28.21 (–C(*C*H_3_)_3_), 39.42 (=C–CH–*C*H_2_–CH–C=), 44.51, 44.58 (C=C–*C*H), 68.61 (*C*H(CH_3_)_2_), 81.85 (–*C*(CH_3_)_3_), 131.41, 131.81 (C–*C*=*C*–C), 141.74, 142.43 (*C*=*C*–C=O), 164.00, 164.72 (C=O).

#### 4.3.12 Polymer 11a

Pale white solid. ^1^H NMR (500 MHz, CDCl_3_) *δ* = 5.50 and 5.42 (br, 2H, *trans* and *cis* =CH), 4.11 (m, 2H, O–C*H*_2_) 3.89 (m, 2H, =CHC*H*), 2.49 (s, 1H, CHC*H*_2_CH), 2.16 (m, 2H, –CH_2_C*H*_2_CH_2_), 1.68 (m, 3H, CHC*H*_2_CH and –C*H*_2_CH_3_), 1.43 (s, 9H, –C(CH_3_)_3_), 0.92 (–CH_3_); ^13^C NMR (125 MHz, CDCl_3_) *δ* = 10.42 (–CH_3_), 17.10 (–*C*H_2_CH_3_), 21.84 (–*C*H_2_CH_3_), 28.11 (–C(*C*H_3_)_3_), 39.32 (=C–CH–*C*H_2_–CH–C=), 44.41, 44.48 (C=C–*C*H), 68.51 (–*C*H_2_–O_2_C), 81.75 (–*C*(CH_3_)_3_), 131.31, 131.71 (C–*C*=*C*–C), 141.64, 142.33 (*C*=*C*–C=O), 163.90, 164.62 (C=O).

#### 4.3.13 Typical procedures for the preparation of amphiphilic polymers with a half-ester

Grubbs' third generation initiator [Ru]-III was adopted to catalyze half-ester monomers under strict exclusion of oxygen. The amounts of added monomers and the [Ru]-III catalyst are shown in Table 3. In a typical procedure, a half-ester (0.24 mmol) and 2,6-di-*tert*-butyl-4-methylphenol (BHT) (0.3 mg, 1.3 μmol) were added to a flame-dried flask, evacuated over 30 min, and dissolved in 0.75 mL of anhydrous THF. The stoichiometric [Ru]-III catalyst was added to another flame-dried flask, evacuated over 30 min, and dissolved in 0.25 mL of anhydrous THF. The monomer solution was added to the [Ru]-III catalyst solution and the reaction mixture was stirred for 15 h at −15 °C. The reaction was quenched with ethyl vinyl ether (1.4 × 10^−2^ mL, 0.14 mmol) and the reaction was continued for 10 min at −15 °C. The polymer was obtained after reprecipitation from cold diethyl ether. The obtained off-white solid was used for the further characterization.

#### 4.3.14 Polymer 12a

Pale white solid. ^1^H NMR (500 MHz, (CD_3_)_2_CO) *δ* = 5.56 and 5.37 (br, 2H, *trans* and *cis* =CH), 3.97 (s, 3H, O–C*H*_3_), 3.61 (d, 2H, =CHC*H*), 2.55 and 1.35 (m, 2H, CHC*H*_2_CH); ^13^C NMR (125 MHz, (CD_3_)_2_CO) *δ* = 39.77 (–*C*H_2_–), 44.83, 52.20 (C=C–*C*H), 55.25 (O–*C*H_3_), 132.01, 132.47 (C–*C*=*C*–C), 143.26, 143.34 (*C*=*C*–C=O), 165.82, 166.19 (C=O).

#### 4.3.15 Polymer 13a

Pale white solid. ^1^H NMR (500 MHz, (CD_3_)_2_CO) *δ* = 5.54 and 5.42 (br, 2H, *trans* and *cis* =CH), 4.12 (s, 2H, O–C*H*_2_–) 4.00 (s, 1H, =CHC*H*), 3.60 (d, 1H, =CHC*H*), 2.60 and 1.39 (m, 2H, CHC*H*_2_CH), 1.16 (t, 3H, –C*H*_3_); ^13^C NMR (125 MHz, (CD_3_)_2_CO) *δ* = 14.32 (–CH_3_), 39.98 (–*C*H_2_–), 45.18, 55.37 (C=C–*C*H), 61.48 (O–*C*H_2_–), 132.40, 132.75 (C–*C*=*C*–C), 142.86, 143.92 (*C*=*C*–C=O), 165.80, 165.95 (C=O).

#### 4.3.16 Polymer 14a

Pale white solid. ^1^H NMR (500 MHz, (CD_3_)_2_CO) *δ* = 5.62 and 5.48 (br, 2H, *trans* and *cis* =CH), 4.09 (m, 2H, O–C*H*_2_), 3.63 (m, 2H, =CHC*H*), 2.66, 1.62 (m, 2H, CHC*H*_2_CH), 1.66 (m, 2H, –C*H*_2_CH_3_), 0.93 (m, 3H, –CH_3_); ^13^C NMR (125 MHz, (CD_3_)_2_CO) *δ* = 10.69 (–CH_3_), 22.33 (–*C*H_2_CH_3_), 39.84 (=C–CH–*C*H_2_–CH–C=), 45.05, 66.95 (C=C–*C*H), 67.87 (–*C*H_2_–O_2_C), 132.31, 132.42 (C–*C*=*C*–C), 142.95, 143.63 (*C*=*C*–C=O), 165.64, 165.83 (C=O).

#### 4.3.17 Polymer 15a

Pale white solid. ^1^H NMR (500 MHz, (CD_3_)_2_CO) *δ* = 5.63 and 5.49 (br, 2H, *trans* and *cis* =CH), 5.03 (m, 1H, C*H*(CH_3_)_2_), 3.62 (m, 2H, =CHC*H*), 2.66, 1.46 (m, 2H, CHC*H*_2_CH), 1.23 (m, 6H, CH(C*H*_3_)_2_); ^13^C NMR (125 MHz, (CD_3_)_2_CO) *δ* = 21.94 (CH(*C*H_3_)_2_), 40.07 (=C–CH–*C*H_2_–CH–C=), 45.28, 55.39 (C=C–*C*H), 69.20 (*C*H(CH_3_)_2_), 132.33, 132.58 (C–*C*=*C*–C), 142.54, 144.55 (*C*=*C*–C=O), 165.65, 165.93 (C=O).

#### 4.3.18 Polymer 16a

Pale white solid. ^1^H NMR (500 MHz, (CD_3_)_2_CO) *δ* = 5.71 and 5.49 (br, 2H, *trans* and *cis* =CH), 4.19 (m, 2H, O–C*H*_2_), 3.67 (m, 2H, =CHC*H*), 2.67, 1.27 (m, 2H, CHC*H*_2_CH), 1.89 (m, 2H, –*CH*_2_CH_2_CH_3_), 1.65 (m, 2H, –C*H*_2_CH_3_), 0.95 (m, 3H, –CH_3_); ^13^C NMR (125 MHz, (CD_3_)_2_CO) *δ* = 9.78 (–CH_3_), 21.09 (–*C*H_2_CH_3_), 28.87 (–*C*H_2_CH_2_CH_3_), 29.49 (=C–CH–*C*H_2_–CH–C=), 45.17, 67.24 (C=C–*C*H), 68.36 (–*C*H_2_–O_2_C), 134.33, 134.72 (C–*C*=*C*–C), 143.46, 144.73 (*C*=*C*–C=O), 165.44, 165.33 (C=O).

#### 4.3.19 Typical procedure for hydrogenation

A 50 mL flask was charged with a polymer (0.24 mmol) obtained by ROMP from monomers 1–16 as described above, *p*-toluenesulfonylhydrazide (TSH, 3.6 mmol), 15 mL of xylene and 2,6-di-butyl-4-methylphenol (BHT, 0.036 mmol) in a nitrogen atmosphere. Polymer 1b was synthesized from polymer 1a from entry 2 in Table 1, and polymers 12b–16b were synthesized from polymers 12a–16a from entries 2, 4, 6, 8, and 10 in Table 3, respectively. The reaction mixture was heated at 135 °C for 5 hours. After the reaction was completed, the mixture was allowed to cool and poured into an excess of methanol. The hydrogenated polymer was further purified by dissolving it in chloroform and reprecipitating it with methanol. The hydrogenated polymer was collected and dried under vacuum.

#### 4.3.20 Polymer 1b

Pale white solid. ^1^H NMR (500 MHz, CDCl_3_) *δ* = 3.69 (s, 6H, –OCH_3_), 2.69 (d, 2H, –CH–CH–, cyclopentane), 2.23 (m, 2H, C*H*–CH_2_–C*H*, cyclopentane), 1.57 (d, 2H, –C*H*_2_–CH), 1.16 (d, 2H, –C*H*_2_–CH), 2.23 and 0.82 (m, 2H, CHC*H*_2_CH); ^13^C NMR (125 MHz, CDCl_3_) *δ* = 33.91 (–*C*H_2_–CH–), 39.05 (CH–*C*H_2_–CH), 42.63 (–CH_2_–*C*H–), 51.89 (O–*C*H_3_), 52.76 (–*C*H–*C*H–), 174.17 (C=O).

#### 4.3.21 Polymer 2b

Pale white solid. ^1^H NMR (500 MHz, CDCl_3_) *δ* = 4.06 (q, 4H, –OCH_2_–), 2.68 (d, 2H, –CH–CH–, cyclopentane), 2.16 (m, 2H, C*H*–CH_2_–C*H*, cyclopentane), 1.54 (s, 2H, –C*H*_2_–CH), 1.20 (d, 8H, –C*H*_2_–CH + –CH_2_C*H*_3_), 2.22 and 0.85 (m, 2H, CHC*H*_2_CH); ^13^C NMR (125 MHz, CDCl_3_) *δ* = 14.24 (–CH_2_*C*H_3_), 34.02 (–*C*H_2_–CH–), 39.15 (CH–*C*H_2_–CH), 42.77 (–CH_2_–*C*H–), 52.70 (–*C*H–*C*H–), 60.48 (O–*C*H_2_–), 173.70 (C=O).

#### 4.3.22 Polymer 3b

Pale white solid. ^1^H NMR (500 MHz, CDCl_3_) *δ* = 3.96 (s, 4H, –OCH_2_–), 2.66 (d, 2H, –CH–CH–, cyclopentane), 2.18 (m, 2H, C*H*–CH_2_–C*H*, cyclopentane), 1.64 (m, 4H, –C*H*_2_–CH_3_), 1.18 (s, 4H, –C*H*_2_–CH), 0.93 (m, 6H, –CH_3_), 2.18 and 0.84 (m, 2H, CHC*H*_2_CH). ^13^C NMR (125 MHz, CDCl_3_) *δ* = 10.57 (–CH_2_*C*H_3_), 21.98 (–*C*H_2_CH_3_), 34.14 (–*C*H_2_–CH–), 39.20 (CH–*C*H_2_–CH), 42.90 (–CH_2_–*C*H–), 52.69 (–*C*H–*C*H–), 66.20 (O–*C*H_2_–), 173.79 (C=O).

#### 4.3.23 Polymer 4b

Pale white solid. ^1^H NMR (500 MHz, CDCl_3_) *δ* = 5.07 (m, 2H, –OCH–), 2.61 (d, 2H, –CH–CH–, cyclopentane), 2.15 (m, 2H, C*H*–CH_2_–C*H*, cyclopentane), 1.59 (s, 2H, –C*H*_2_–CH), 1.24 (d, 14H, –CH(C*H*_3_)_2_ + –C*H*_2_–CH), 2.42 and 0.82 (m, 2H, CHC*H*_2_CH); ^13^C NMR (125 MHz, CDCl_3_) *δ* = 21.99 (–CH(*C*H_3_)_2_), 34.30 (–*C*H_2_–CH–), 39.18 (CH–*C*H_2_–CH), 43.04 (–CH_2_–*C*H–), 52.71 (–*C*H–*C*H–), 67.74 (O–*C*H–), 173.25 (C=O).

#### 4.3.24 Polymer 5b

Pale white solid. ^1^H NMR (500 MHz, CDCl_3_) *δ* = 4.01 (m, 4H, –OCH_2_–), 2.70 (d, 2H, –CH–CH–, cyclopentane), 2.16 (m, 2H, C*H*–CH_2_–C*H*, cyclopentane), 1.56 (m, 4H, –C*H*_2_–CH_2_–CH_3_), 1.36 (m, 4H, –C*H*_2_–CH_3_), 1.17 (s, 4H, –C*H*_2_–CH), 0.92 (m, 6H, –CH_3_), 2.21 and 0.82 (m, 2H, CHC*H*_2_CH). ^13^C NMR (125 MHz, CDCl_3_) *δ* = 13.86 (–CH_2_*C*H_3_), 19.29 (–*C*H_2_CH_3_), 30.72 (–*C*H_2_CH_2_CH_3_), 34.19 (–*C*H_2_–CH–), 39.21 (CH–*C*H_2_–CH), 42.96 (–CH_2_–*C*H–), 52.75 (–*C*H–*C*H–), 64.49 (O–*C*H_2_–), 173.80 (C=O).

#### 4.3.25 Polymer 6b

Pale white solid. ^1^H NMR (500 MHz, CDCl_3_) *δ* = 2.61 (d, 2H, –CH–CH–, cyclopentane), 2.14 (s, 2H, C*H*–CH_2_–C*H*, cyclopentane), 1.61 (s, 2H, –C*H*_2_–CH), 1.42 (s, 18H, –C(C*H*_3_)_3_), 1.25 (s, 2H, –C*H*_2_–CH), 0.80 (m, 2H, CHC*H*_2_CH); ^13^C NMR (125 MHz, CDCl_3_) *δ* = 28.41 (–C(*C*H_3_)_3_), 34.65 (–*C*H_2_–CH–), 39.34 (CH–*C*H_2_–CH), 43.12 (–CH_2_–*C*H–), 53.24 (–*C*H–*C*H–), 80.19 (O–*C*–), 173.27 (C=O).

#### 4.3.26 Polymer 7b

Pale white solid. ^1^H NMR (500 MHz, CDCl_3_) *δ* = 3.63 (s, 3H, –OCH_3_), 2.60 (d, 2H, –CH–CH–, cyclopentane), 2.15 (s, 2H, C*H*–CH_2_–C*H*, cyclopentane), 1.58 (d, 2H, –C*H*_2_–CH), 1.41 (s, 9H, –C(CH_3_)_3_), 1.23 (m, 2H, –C*H*_2_–CH), 2.22 and 0.83 (m, 2H, CHC*H*_2_CH); ^13^C NMR (125 MHz, CDCl_3_) *δ* = 28.13 (–C(*C*H_3_)_3_), 34.28 (–*C*H_2_–CH–), 39.17 (–CH–*C*H_2_–CH–), 42.55, 43.38 (–CH_2_–*C*H–), 51.60 (O–*C*H_3_), 52.57, 53.67 (–*C*H–*C*H–), 80.57 (–*C*(CH_3_)_3_), 173.08, 174.26 (C=O).

#### 4.3.27 Polymer 8b

Pale white solid. ^1^H NMR (500 MHz, CDCl_3_) *δ* = 4.06 (m, 2H, –OCH_2_–), 2.59 (s, 2H, –CH–CH–, cyclopentane), 2.15 (s, 2H, C*H*–CH_2_–C*H*, cyclopentane), 1.58 (d, 2H, –C*H*_2_–CH), 1.43 (s, 9H, –C(CH_3_)_3_), 1.24 (m, 5H, –C*H*_2_–CH + –CH_2_C*H*_3_), 2.15 and 0.83 (m, 2H, CHC*H*_2_CH); ^13^C NMR (125 MHz, CDCl_3_) *δ* = 14.75 (–CH_2_*C*H_3_), 28.58 (–C(*C*H_3_)_3_), 34.75, 34.92 (–*C*H_2_–CH–), 39.67 (–CH–*C*H_2_–CH–), 43.22, 43.73 (–CH_2_–*C*H–), 53.06, 53.99 (–*C*H–*C*H–), 60.81 (O–*C*H_2_–), 80.92 (–*C*(CH_3_)_3_), 173.50, 174.33 (C=O).

#### 4.3.28 Polymer 9b

Pale white solid. ^1^H NMR (500 MHz, CDCl_3_) *δ* = 4.06 (m, 2H, –OCH_2_–), 2.60 (s, 2H, –CH–CH–, cyclopentane), 2.18 (s, 2H, C*H*–CH_2_–C*H*, cyclopentane), 1.64 (m, 2H, –C*H*_2_–CH_3_), 1.46 (s, 2H, –C*H*_2_–CH), 1.42 (s, 9H, –C(CH_3_)_3_), 1.20 (s, 2H, –C*H*_2_–CH), 0.91 (t, 3H, –CH_3_), 0.80 (m, 2H, CHC*H*_2_CH); ^13^C NMR (125 MHz, CDCl_3_) *δ* = 10.67 (–CH_2_*C*H_3_), 22.06 (–*C*H_2_CH_3_), 28.15 (–C(*C*H_3_)_3_), 34.31, 34.51 (–*C*H_2_–CH–), 39.27 (–CH–*C*H_2_–CH–), 42.86, 43.24 (–CH_2_–*C*H–), 52.69, 53.54 (–*C*H–*C*H–), 66.07 (O–*C*H_2_–), 80.46 (–*C*(CH_3_)_3_), 173.01, 173.97 (C=O).

#### 4.3.29 Polymer 10b

Pale white solid. ^1^H NMR (500 MHz, CDCl_3_) *δ* = 4.95 (m, 1H, –OCH–), 2.57 (s, 2H, –CH–CH–, cyclopentane), 2.17 (d, 2H, C*H*–CH_2_–C*H*, cyclopentane), 1.60 (d, 2H, –C*H*_2_–CH), 1.41 (s, 9H, –C(CH_3_)_3_), 1.22 (m, 8H, –C*H*_2_–CH + –CH(C*H*_3_)_2_), 2.43 and 0.83 (m, 2H, CHC*H*_2_CH); ^13^C NMR (125 MHz, CDCl_3_) *δ* = 21.94 (–CH(*C*H_3_)_2_), 28.21 (–C(*C*H_3_)_3_), 34.35, 34.68 (–*C*H_2_–CH–), 39.26 (–CH–*C*H_2_–CH–), 43.01, 43.16 (–CH_2_–*C*H–), 52.67, 53.37 (–*C*H–*C*H–), 67.69 (O–*C*H–), 80.40 (–*C*(CH_3_)_3_), 173.02, 173.47 (C=O).

#### 4.3.30 Polymer 11b

Pale white solid. ^1^H NMR (500 MHz, CDCl_3_) *δ* = 4.02 (m, 2H, –OCH_2_–), 2.59 (s, 2H, –CH–CH–, cyclopentane), 2.39 (s, 2H, C*H*–CH_2_–C*H*, cyclopentane), 2.16 (br, 2H, –C*H*_2_–CH_2_–CH_3_), 1.58 (m, 2H, –C*H*_2_–CH_3_), 1.43 (s, 2H, –C*H*_2_–CH), 1.39 (s, 9H, –C(CH_3_)_3_), 1.25 (m, 2H, –C*H*_2_–CH), 0.93, 0.83 (m, 5H, CHC*H*_2_CH + –CH_3_); ^13^C NMR (125 MHz, CDCl_3_) *δ* = 13.67 (–CH_2_*C*H_3_), 25.06 (–*C*H_2_CH_3_), 28.56 (–*C*H_2_CH_2_CH_3_), 31.15 (–C(*C*H_3_)_3_), 37.31, 37.40 (–*C*H_2_–CH–), 42.27 (–CH–*C*H_2_–CH–), 45.83, 46.22 (–CH_2_–*C*H–), 55.67, 56.51 (–*C*H–*C*H–), 69.10 (O–*C*H_2_–), 83.45 (–*C*(CH_3_)_3_), 176.01, 176.98 (C=O).

#### 4.3.31 Polymer 12b

Pale white solid. ^1^H NMR (500 MHz, (CD_3_)_2_CO) *δ* = 3.64 (s, 3H, –OCH_3_), 2.62 (d, 2H, –CH–CH–, cyclopentane), 2.17 (s, 2H, C*H*–CH_2_–C*H*, cyclopentane), 1.54 (d, 2H, –C*H*_2_–CH), 1.25 (m, 2H, –C*H*_2_–CH), 2.22 and 0.82 (m, 2H, CHC*H*_2_CH); ^13^C NMR (125 MHz, (CD_3_)_2_CO) *δ* = 34.33, 34.39 (–*C*H_2_–CH–), 39.28 (–CH–*C*H_2_–CH–), 42.70, 43.51 (–CH_2_–*C*H–), 51.72 (O–*C*H_3_), 52.69, 53.78 (–*C*H–*C*H–), 172.95, 174.01 (C=O).

#### 4.3.32 Polymer 13b

Pale white solid. ^1^H NMR (500 MHz, (CD_3_)_2_CO) *δ* = 4.23 (m, 2H, –OCH_2_–), 2.71 (s, 2H, –CH–CH–, cyclopentane), 2.28 (s, 2H, C*H*–CH_2_–C*H*, cyclopentane), 1.59 (d, 2H, –C*H*_2_–CH), 1.19 (m, 5H, –C*H*_2_–CH + –CH_2_C*H*_3_), 2.00 and 0.98 (m, 2H, CHC*H*_2_CH); ^13^C NMR (125 MHz, (CD_3_)_2_CO) *δ* = 14.41 (–CH_2_*C*H_3_), 33.98, 34.86 (–*C*H_2_–CH–), 39.41 (–CH–*C*H_2_–CH–), 42.82, 43.43 (–CH_2_–*C*H–), 52.65, 52.98 (–*C*H–*C*H–), 61.01 (O–*C*H_2_–), 172.88, 174.05 (C=O).

#### 4.3.33 Polymer 14b

Pale white solid. ^1^H NMR (500 MHz, (CD_3_)_2_CO) *δ* = 4.05 (m, 2H, –OCH_2_–), 2.68 (s, 2H, –CH–CH–, cyclopentane), 2.21 (s, 2H, C*H*–CH_2_–C*H*, cyclopentane), 1.66 (m, 2H, –C*H*_2_–CH_3_), 1.43 (s, 2H, –C*H*_2_–CH), 1.21 (s, 2H, –C*H*_2_–CH), 0.98 (t, 3H, –CH_3_), 0.81 (d, 2H, CHC*H*_2_CH); ^13^C NMR (125 MHz, (CD_3_)_2_CO) *δ* = 10.65 (–CH_2_*C*H_3_), 22.75 (–*C*H_2_CH_3_), 34.39, 34.58 (–*C*H_2_–CH–), 39.24 (–CH–*C*H_2_–CH–), 42.92, 43.31 (–CH_2_–*C*H–), 52.64, 53.47 (–*C*H–*C*H–), 60.28 (O–*C*H_2_–), 172.87, 173.97 (C=O).

#### 4.3.34 Polymer 15b

Pale white solid. ^1^H NMR (500 MHz, (CD_3_)_2_CO) *δ* = 5.01 (m, 1H, –OCH–), 2.61 (s, 2H, –CH–CH–, cyclopentane), 2.21 (d, 2H, C*H*–CH_2_–C*H*, cyclopentane), 1.64 (d, 2H, –C*H*_2_–CH), 1.26 (m, 8H, –C*H*_2_–CH + –CH(C*H*_3_)_2_), 2.47 and 0.87 (m, 2H, CHC*H*_2_CH); ^13^C NMR (125 MHz, (CD_3_)_2_CO) *δ* = 21.23 (–CH(*C*H_3_)_2_), 34.73, 34.92 (–*C*H_2_–CH–), 39.87 (–CH–*C*H_2_–CH–), 44.97, 45.11 (–CH_2_–*C*H–), 52.29, 52.66 (–*C*H–*C*H–), 67.99 (O–*C*H–), 173.11, 173.56 (C=O).

#### 4.3.35 Polymer 16b

Pale white solid. ^1^H NMR (500 MHz, (CD_3_)_2_CO) *δ* = 4.23 (m, 2H, –OCH_2_–), 2.67 (s, 2H, –CH–CH–, cyclopentane), 2.39 (s, 2H, C*H*–CH_2_–C*H*, cyclopentane), 1.98 (m, 2H, –C*H*_2_–CH_3_), 1.76 (m, 2H, –C*H*_2_–CH_2_–CH_3_), 1.44 (s, 2H, –C*H*_2_–CH), 1.22 (s, 2H, –C*H*_2_–CH), 0.96 (t, 3H, –CH_3_), 0.85 (d, 2H, CHC*H*_2_CH); ^13^C NMR (125 MHz, (CD_3_)_2_CO) *δ* = 12.98 (–CH_2_*C*H_3_), 18.67 (–*C*H_2_CH_3_), 24.32 (–*C*H_2_CH_2_CH_3_), 36.47, 37.35 (–*C*H_2_–CH–), 43.77 (–CH–*C*H_2_–CH–), 47.42, 49.33 (–CH_2_–*C*H–), 54.12, 55.88 (–*C*H–*C*H–), 62.63 (O–*C*H_2_–), 173.22, 173.98 (C=O).

## Conflicts of interest

There are no conflicts to declare.

## Supplementary Material

RA-013-D2RA07779E-s001

## References

[cit1] Davidson T. A., Wagener A., Priddy D. (1996). Polymerization of dicyclopentadiene: A tale of two mechanisms. Macromolecules.

[cit2] Chen Q. C. (2021). Surface-initiated ring-opening metathesis polymerization (SI-ROMP): History, general features, and applications in surface engineering with polymer brushes. Int. J. Polym. Sci..

[cit3] Chauvin Y. (2006). Olefin metathesis: The early days (Nobel Lecture). Angew. Chem., Int. Ed..

[cit4] Grubbs R. H. (2006). Olefin-metathesis catalysts for the preparation of molecules and materials (Nobel Lecture). Angew. Chem., Int. Ed..

[cit5] Anderson D. R., Ung T., Mkrtumyan G., Bertrand G., Grubbs R. H., Schrodi Y. (2008). Kinetic selectivity of olefin metathesis catalysts bearing cyclic (alkyl)(amino)carbenes. Organometallics.

[cit6] Ogba O. M., Warner N. C., O'Leary D. J., Grubbs R. H. (2018). Recent advances in ruthenium-based olefin metathesis. Chem. Soc. Rev..

[cit7] Lienkamp K., Kins C. F., Alfred S. F., Madkour A. E., Tew G. N. (2009). Water-soluble polymers from acid-functionalized norbornenes. J. Polym. Sci., Part A: Polym. Chem..

[cit8] Chen J., Li H. F., Zhang H. C., Liao X. J., Han H. J., Zhang L. D., Sun R. Y., Xie M. R. (2018). Blocking-cyclization technique for precise synthesis of cyclic polymers with regulated topology. Nat. Commun..

[cit9] Shi J., Hayashishita Y., Takata T., Nishihara Y., Niwayama S. (2020). Syntheses of polynorbornadienes by ring-opening metathesis polymerizations of symmetric and nonsymmetric 2,3-bis(alkoxycarbonyl) norbornadienes and their conversion to half-ester derivatives. Org. Biomol. Chem..

[cit10] Niwayama S. (2000). Highly efficient selective monohydrolysis of symmetric diesters. J. Org. Chem..

[cit11] Sanford M. S., Ulman M., Grubbs R. H. (2001). New insights into the mechanism of ruthenium-catalyzed olefin metathesis reactions. J. Am. Chem. Soc..

[cit12] Sanford M. S., Love J. A., Grubbs R. H. (2001). Mechanism and activity of ruthenium olefin metathesis catalysts. J. Am. Chem. Soc..

[cit13] Nishihara Y., Izawa S., Inoue Y., Nakayama Y., Shiono T., Takagi K. (2008). Synthesis, characterization, and thermal properties of ring-opening metathesis polynorbornenes and their hydrogenated derivatives bearing various ester and cyano groups. J. Polym. Sci., Part A: Polym. Chem..

[cit14] Nishihara Y., Doi Y., Izawa S., Li H.-Y., Inoue Y., Kojima M., Chen J.-T., Takagi K. (2010). Enantioseparation of doubly functionalized polar norbornenes by HPLC and their ruthenium-catalyzed ring-opening methathesis polymerization. Rapid Communication. J. Polym. Sci., Part A: Polym. Chem..

[cit15] Nishihara Y., Inoue Y., Nakayama Y., Shiono T., Takagi K. (2006). Comparative reactivity of exo- and endo-isomers in the Ru-initiated ring-opening metathesis polymerization of doubly functionalized norbornenes with both cyano and ester groups. Macromolecules.

[cit16] Delaude L., Demonceau A., Noels A. F. (1999). Highly stereoselective Ruthenium-catalyzed ring-opening metathesis polymerization of 2,3-difunctionalized norbornadienes and their 7-oxaanalogues. Macromolecules.

[cit17] Khosravi E., Feast W. J., Al-Hajaji A. A., Leejarkpai T. (2000). ROMP of n-alkyl norbornene dicarboxyimides: from classical to well-defined initiators, an overview. J. Mol. Catal. A: Chem..

[cit18] Benedicto A. D., Novak B. M., Grubbs R. H. (1992). Microstructural studies of poly(7-oxabicyclo[2.2.1]hept-2-ene) derivatives prepared from selected ruthenium catalysts. Macromolecules.

[cit19] Bartoli G., Bosco M., Carlone A., Dalpozzo R., Marcantoni E., Melchiorre P., Sambri L. (2007). Reaction of dicarbonates with carboxylic acids catalyzed by weak Lewis acids: General method for the synthesis of anhydrides and esters. Synthesis.

[cit20] Amir-Ebrahimi V., Corry D. A. K., Hamilton J. G., Rooney J. J. (1998). Determination of the tacticities of ring-opened metathesis polymers of symmetrical 5,6-disubstituted derivatives of norbornene and norbornadiene from the ^13^C NMR spectra of their hydrogenated derivatives. J. Mol. Catal. A: Chem..

