# Supplementary material for: Synthesis of polynorbornadienes by ring-opening metathesis polymerization and their saturated derivatives bearing various ester groups and carboxyl groups
Source: RSC Adv. 2023 Jan 24;13(6):3494–504. doi: 10.1039/d2ra07779e (PMC9872776; doi:10.1039/d2ra07779e)
Supplement: RA-013-D2RA07779E-s001 [file RA-013-D2RA07779E-s001.pdf]

Electronic Supplementary Material (ESI) for RSC Advances.
This journal is © The Royal Society of Chemistry 2023

Synthesis, characterization and thermal properties of polynorbornadienes by ring-opening metathesis polymerizations and their hydrogenated derivatives bearing various ester and carboxylic groups

Xiaoxue Lin[a], Jianjun Shi[a,b*], Satomi Niwayama[b*]

[a] *College of Chemistry and Chemical Engineering, Hainan Normal University, Haikou, Hainan 571158, P. R. China.*

[b] *Division of Sustainable and Environmental Engineering, Graduate School of Engineering, Muroran Institute of Technology, 27-1, Mizumoto-cho, Muroran, Hokkaido, 050-8585, Japan.*

E-mail: niwayama@mmm.muroran-it.ac.jp

Table of Contents

1. ^{1}H NMR and 13C NMR spectrum in $CDCl_3$ of symmetric polymers, **1a-6a** prepared using Grubbs' third generation catalyst **S2**

2. ^{1}H NMR and ^{13}C NMR spectrum in $CDCl_3$ of non-symmetric polymers, **7a-11a** prepared using Grubbs' third generation catalyst **S8**

3. ^{1}H NMR and ^{13}C NMR spectrum in acetone-*d*6 of amphiphilic polymers, **12a-16a** prepared using Grubbs' third generation catalyst **S13**

4. ^{1}H NMR and ^{13}C NMR spectrum of hydrogenated polymers **1b-11b** in $CDCl_3$ **S18**

5. ^{1}H NMR and ^{13}C NMR spectrum of hydrogenated polymers, **12b-16b** in acetone-*d*6 **S29**

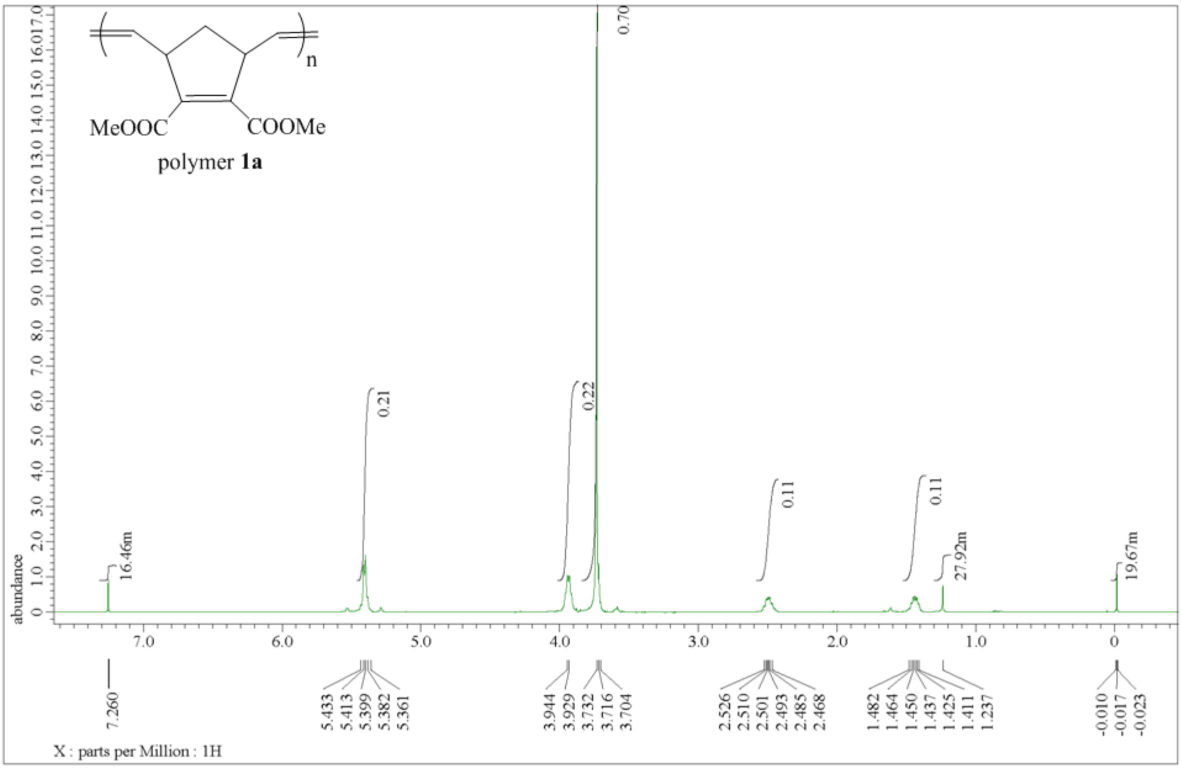
MeOOC
COOMe
n
polymer **1a**
abundance
16.46m
0.21
0.22
0.70
0.11
0.11
27.92m
19.67m
7.260
5.433
5.413
5.399
5.382
5.361
3.944
3.929
3.732
3.716
3.704
2.526
2.510
2.501
2.493
2.485
2.468
1.482
1.464
1.450
1.437
1.425
1.411
1.237
-0.010
-0.017
-0.023
X : parts per Million : 1H

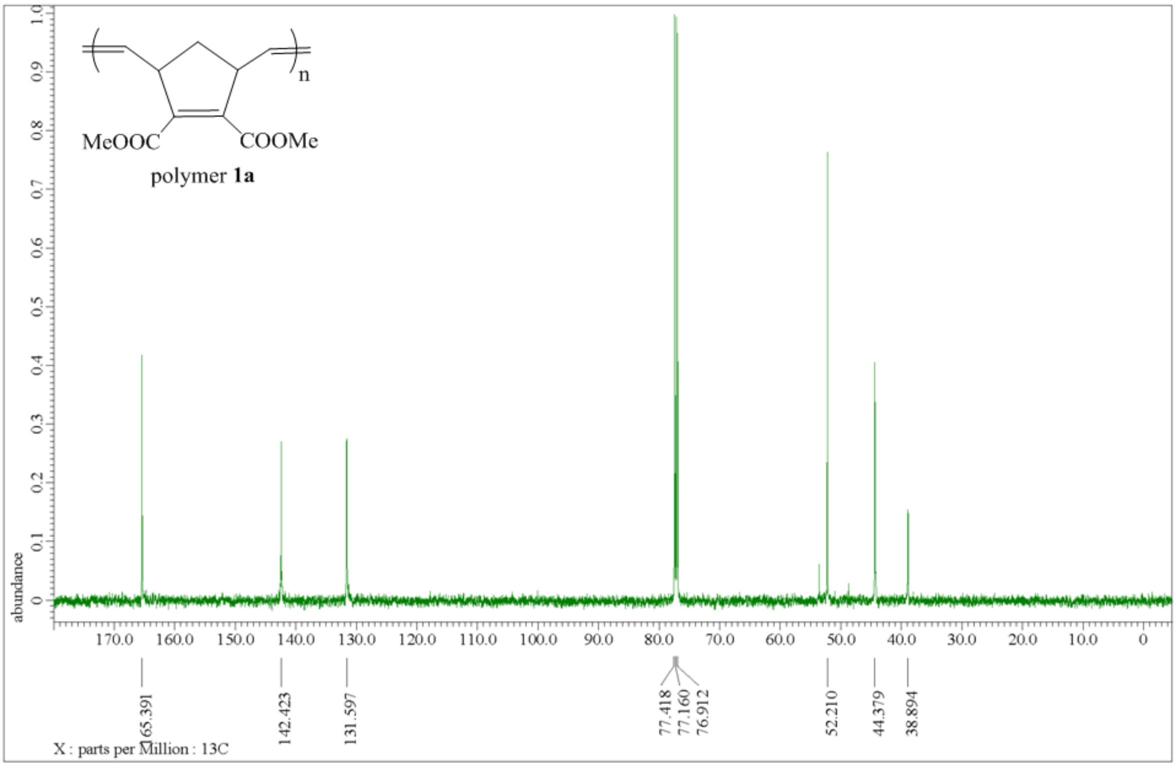
MeOOC
COOMe
n
polymer **1a**
abundance
165.391
142.423
131.597
77.418
77.160
76.912
52.210
44.379
38.894
X : parts per Million : 13C

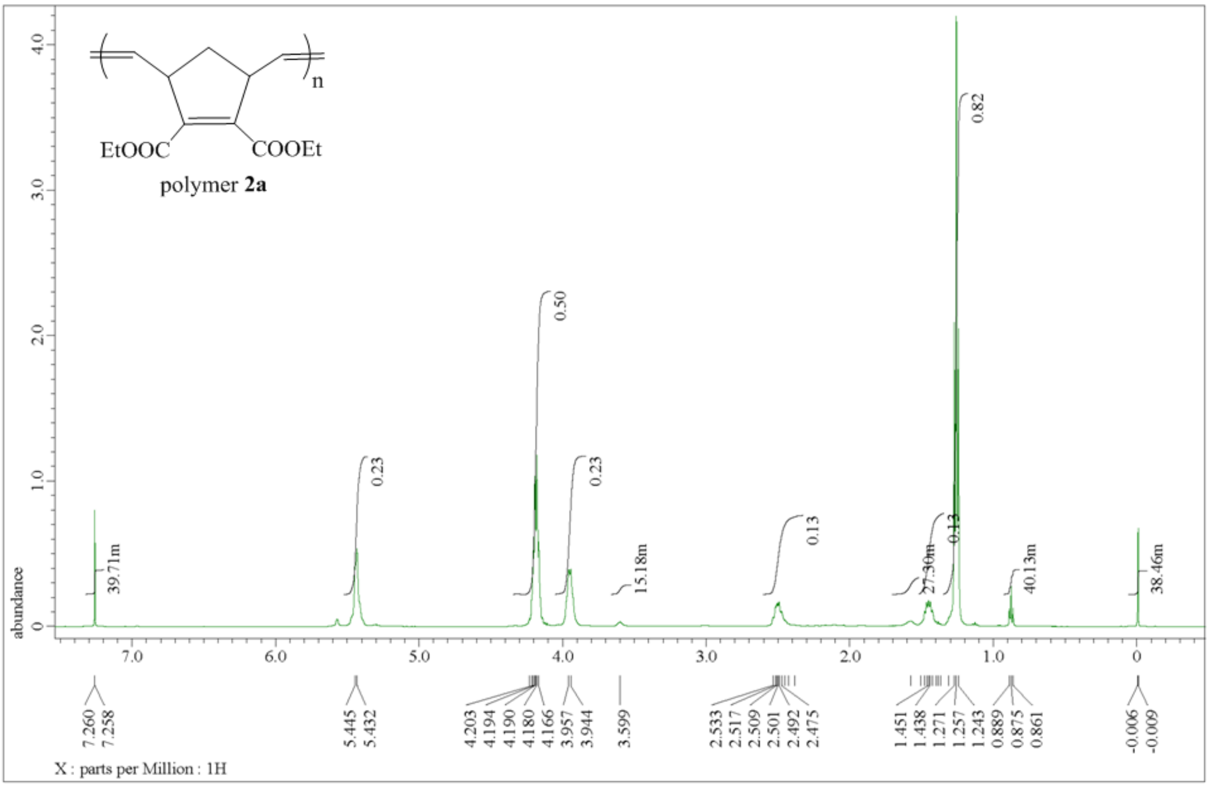
EtOOC
COOEt
polymer 2a
n
abundance
X : parts per Million : 1H
39.71m
0.23
0.50
0.23
15.18m
0.13
27.30m
0.13
0.82
40.13m
38.46m
7.260
7.258
5.445
5.432
4.203
4.194
4.190
4.180
4.166
3.957
3.944
3.599
2.533
2.517
2.509
2.501
2.492
2.475
1.451
1.438
1.271
1.257
1.243
0.889
0.875
0.861
-0.006
-0.009

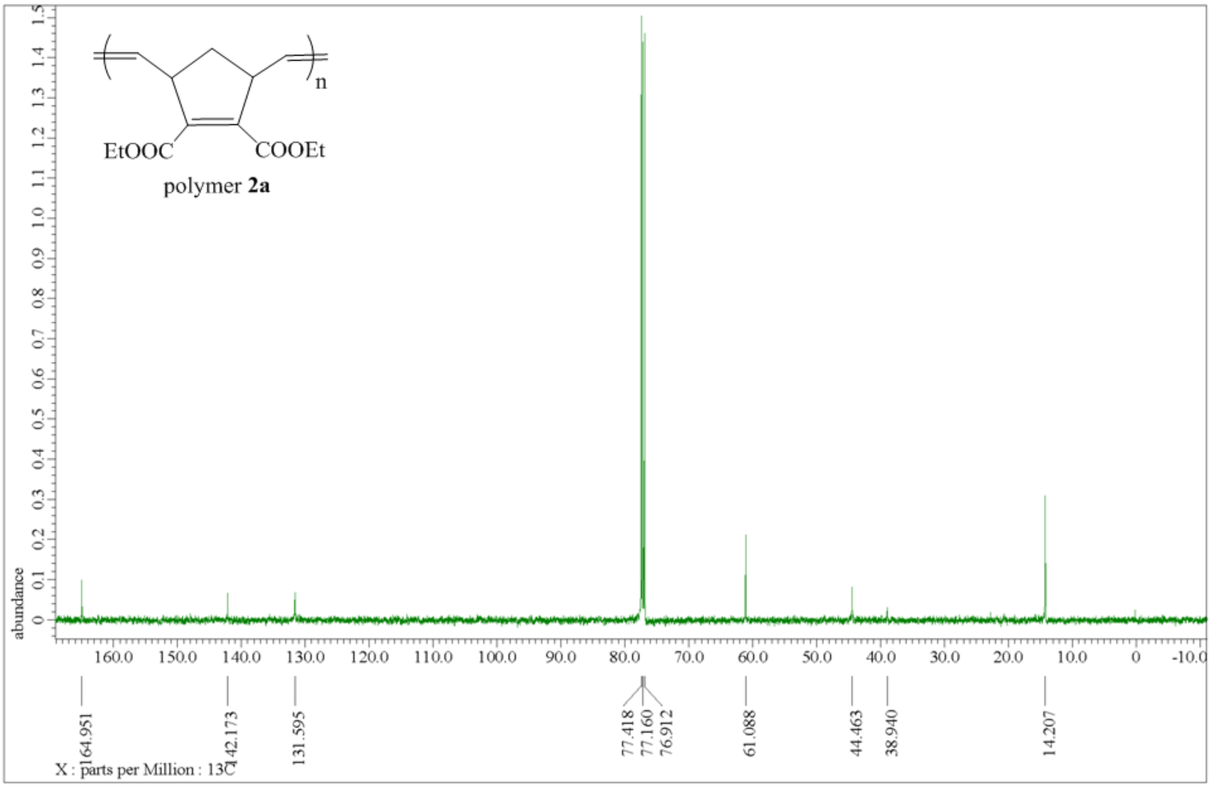
EtOOC
COOEt
polymer 2a
n
abundance
X : parts per Million : 13C
164.951
142.173
131.595
77.418
77.160
76.912
61.088
44.463
38.940
14.207

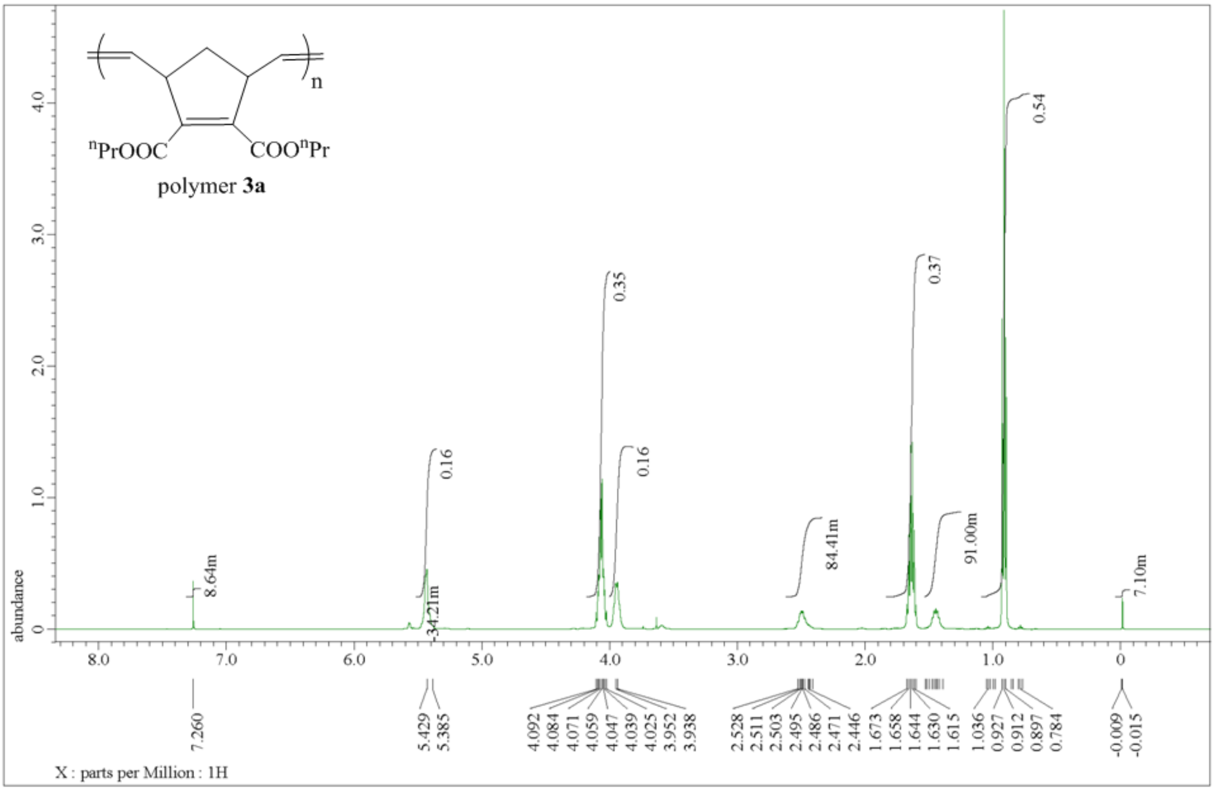
polymer 3a
X : parts per Million : 1H
abundance
7.260
5.429
5.385
4.092
4.084
4.071
4.059
4.047
4.039
4.025
3.952
3.938
2.528
2.511
2.503
2.495
2.486
2.471
2.446
1.673
1.658
1.644
1.630
1.615
1.036
0.927
0.912
0.897
0.784
-0.009
-0.015
8.64m
34.21m
0.16
0.35
0.16
84.41m
0.37
91.00m
0.54
7.10m

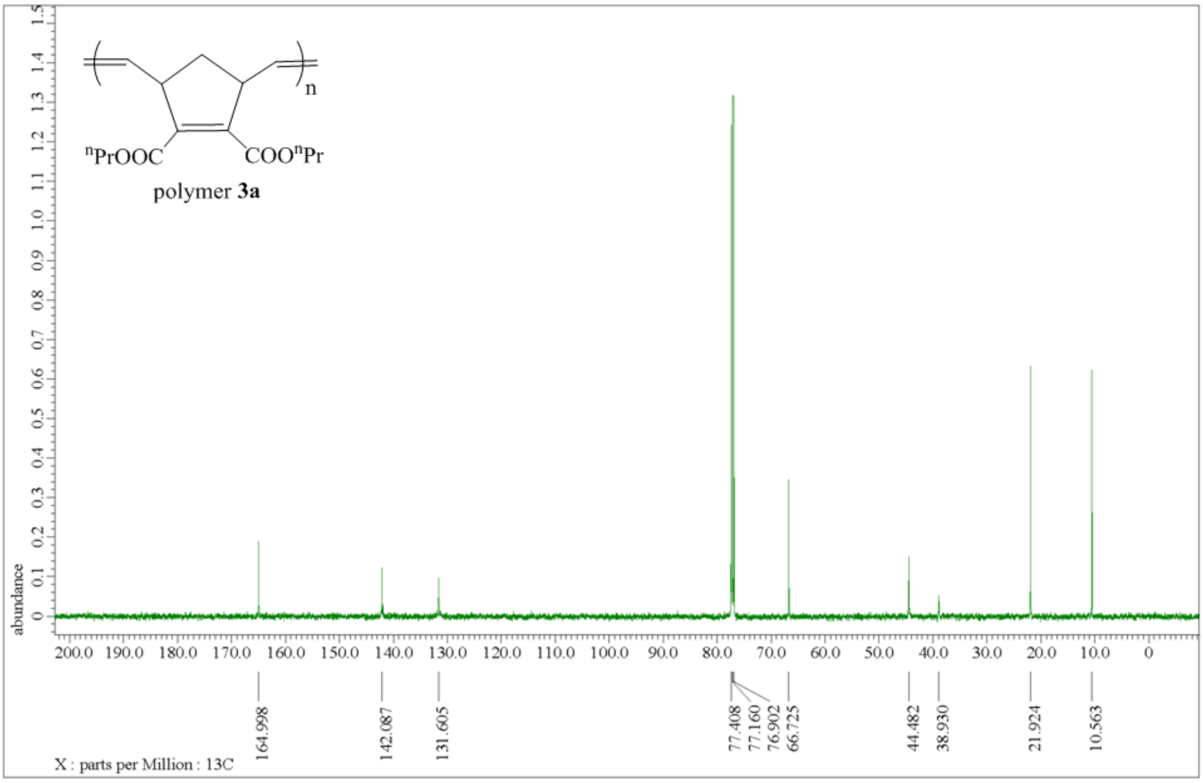
polymer 3a
X : parts per Million : 13C
abundance
164.998
142.087
131.605
77.408
77.160
76.902
66.725
44.482
38.930
21.924
10.563

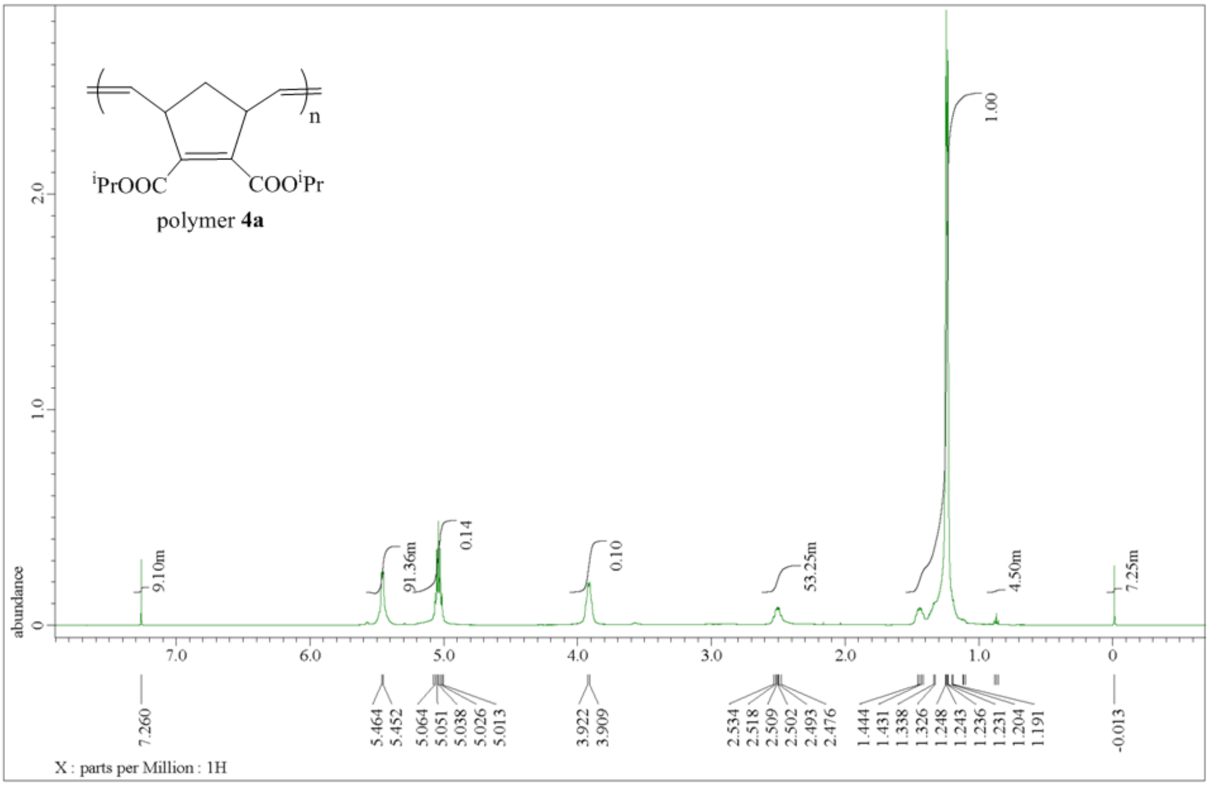

iPrOOC
COOiPr
n
polymer 4a
9.10m
91.36m
0.14
0.10
53.25m
1.00
4.50m
7.25m
abundance
7.260
5.464
5.452
5.064
5.051
5.038
5.026
5.013
3.922
3.909
2.534
2.518
2.509
2.502
2.493
2.476
1.444
1.431
1.338
1.326
1.248
1.243
1.236
1.231
1.204
1.191
-0.013
X : parts per Million : 1H

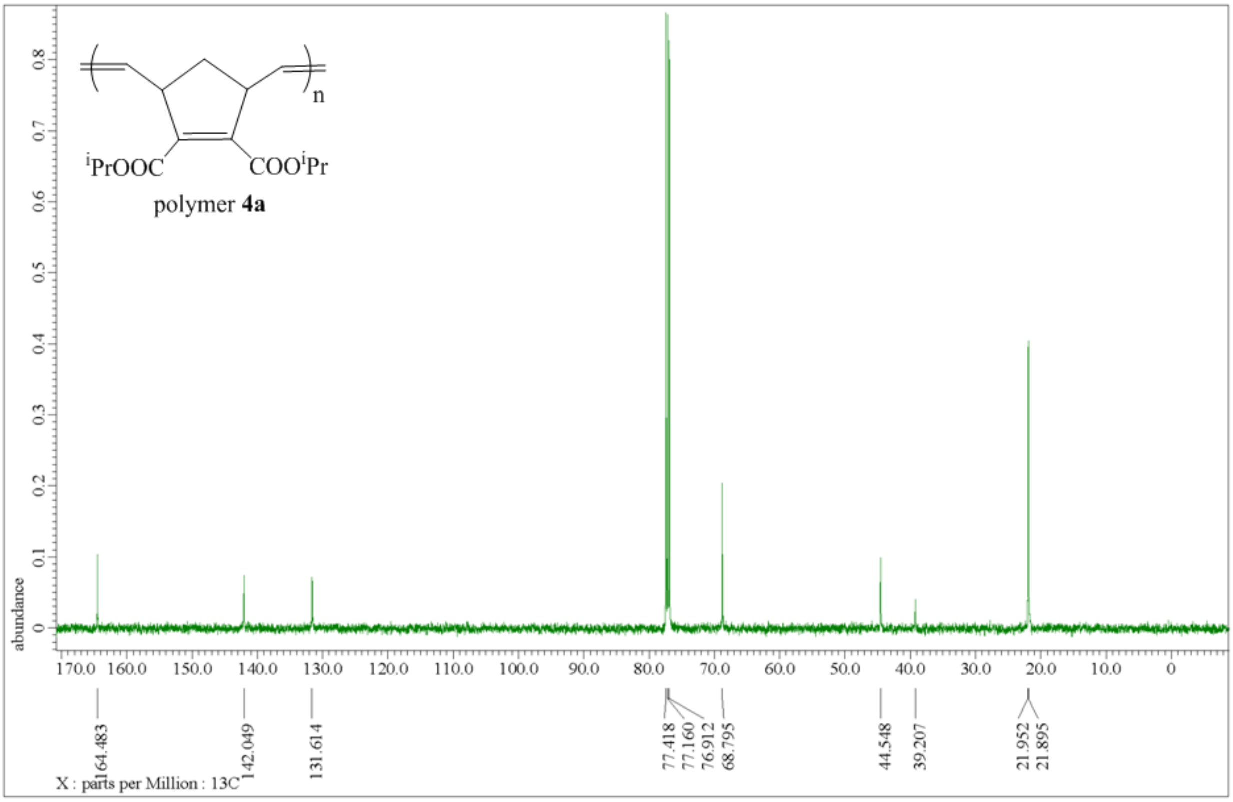

iPrOOC
COOiPr
n
polymer 4a
abundance
164.483
142.049
131.614
77.418
77.160
76.912
68.795
44.548
39.207
21.952
21.895
X : parts per Million : 13C

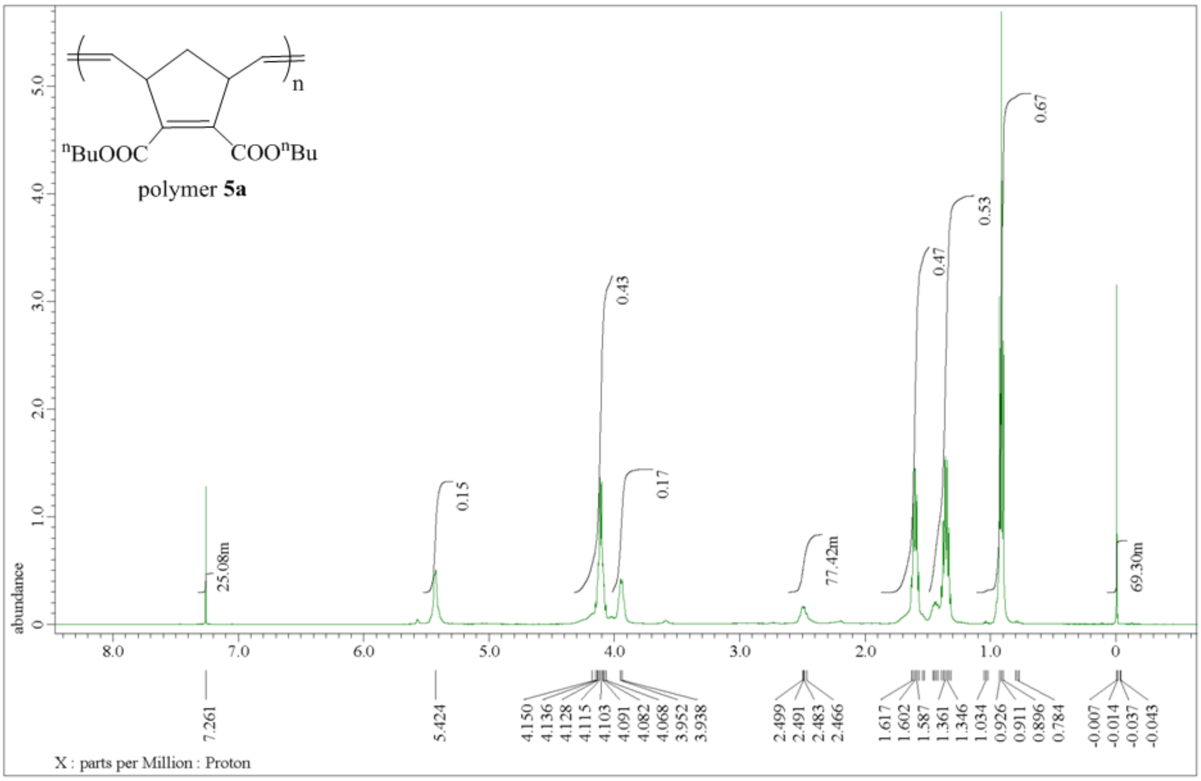

polymer 5a
^{n}BuOOC
COO^{n}Bu
n
abundance
X : parts per Million : Proton
7.261
5.424
4.150
4.136
4.128
4.115
4.103
4.091
4.082
4.068
3.952
3.938
2.499
2.491
2.483
2.466
1.617
1.602
1.587
1.361
1.346
1.034
0.926
0.911
0.896
0.784
-0.007
-0.014
-0.037
-0.043
25.08m
0.15
0.43
0.17
77.42m
0.47
0.53
0.67
69.30m

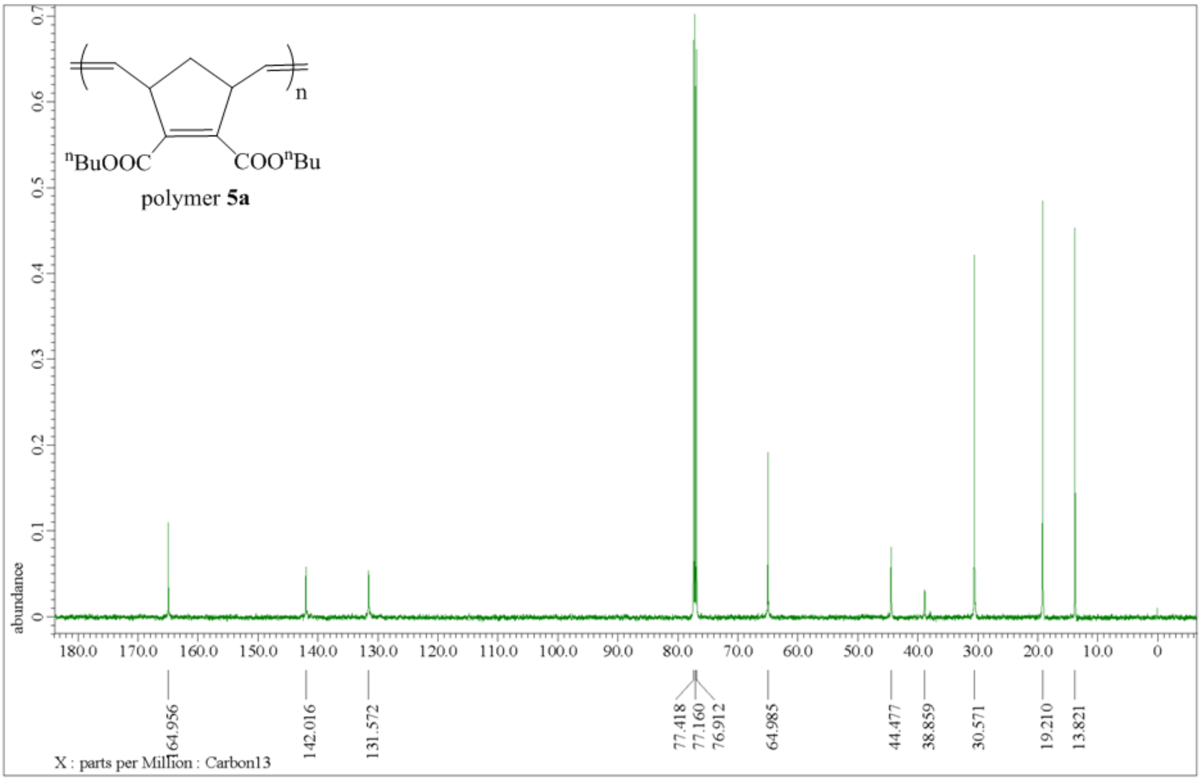

polymer 5a
^{n}BuOOC
COO^{n}Bu
n
abundance
X : parts per Million : Carbon13
164.956
142.016
131.572
77.418
77.160
76.912
64.985
44.477
38.859
30.571
19.210
13.821

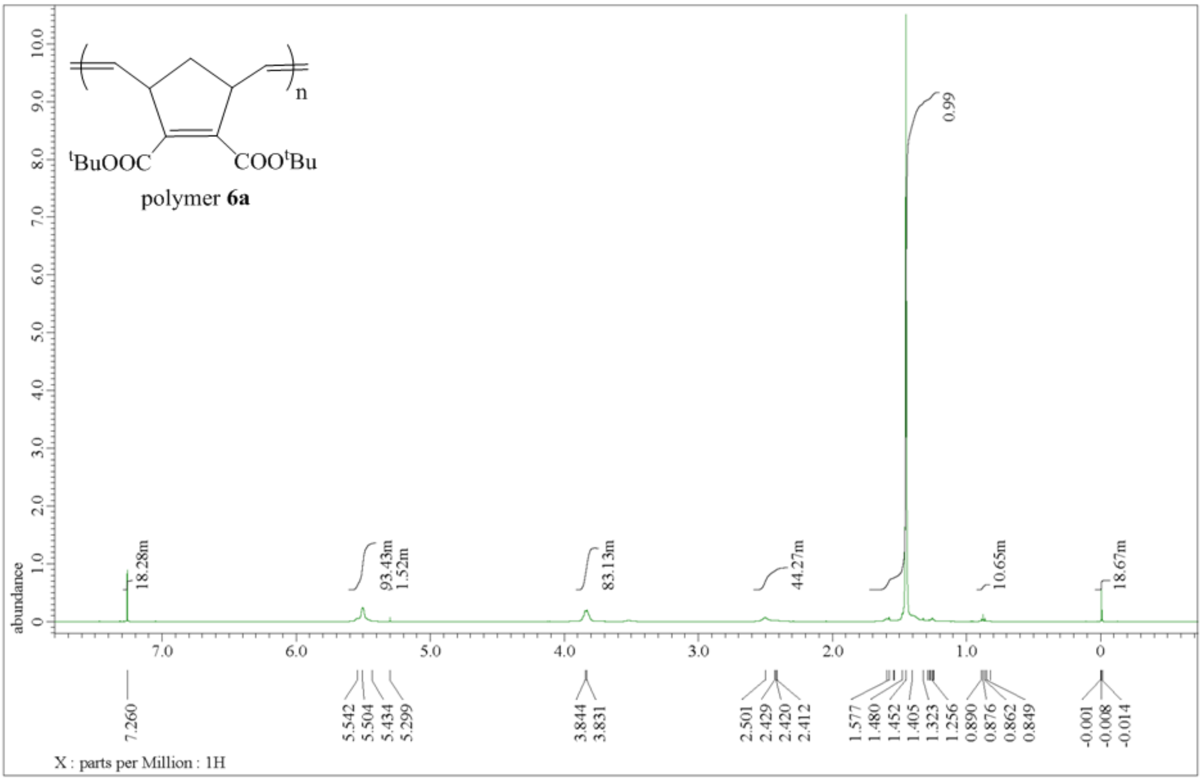
polymer **6a**
^tBuOOC
COO^tBu
n
abundance
X : parts per Million : 1H
18.28m
93.43m
1.52m
83.13m
44.27m
0.99
10.65m
18.67m
7.260
5.542
5.504
5.434
5.299
3.844
3.831
2.501
2.429
2.420
2.412
1.577
1.480
1.452
1.405
1.323
1.256
0.890
0.876
0.862
0.849
-0.001
-0.008
-0.014

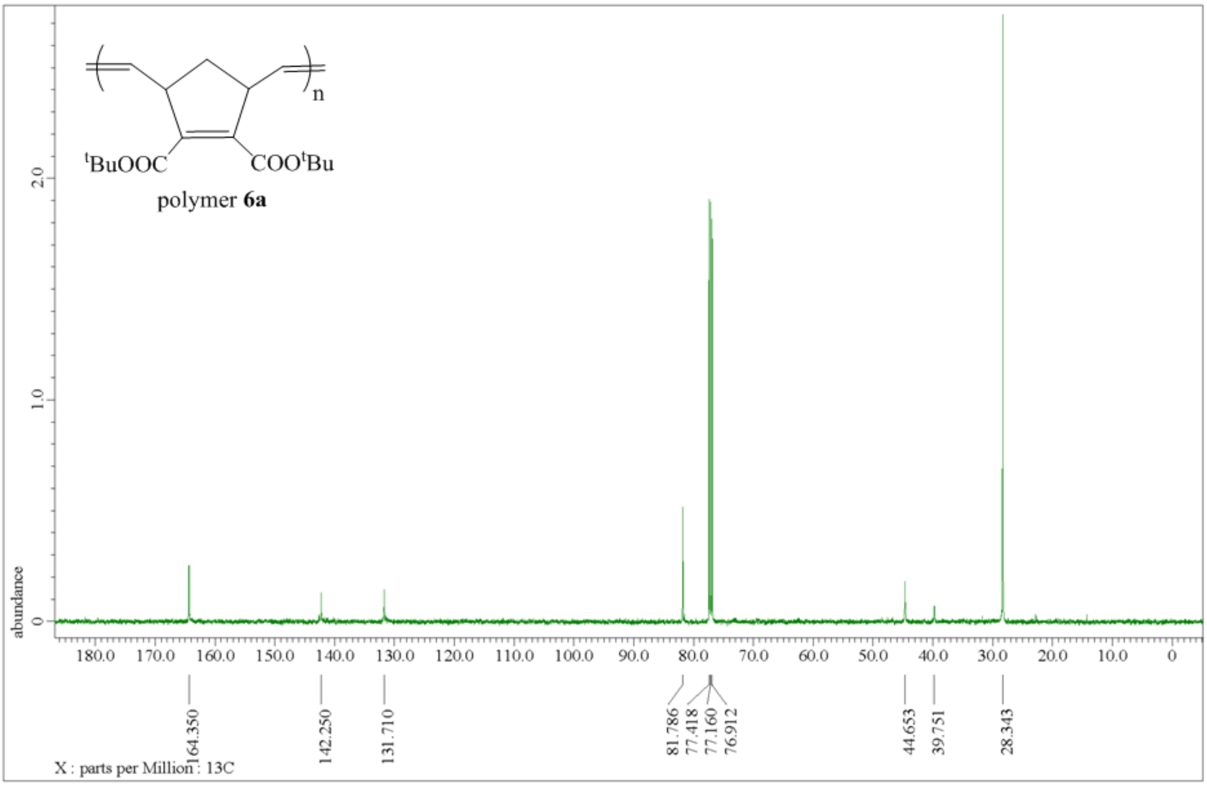
polymer **6a**
^tBuOOC
COO^tBu
n
abundance
X : parts per Million : 13C
164.350
142.250
131.710
81.786
77.418
77.160
76.912
44.653
39.751
28.343

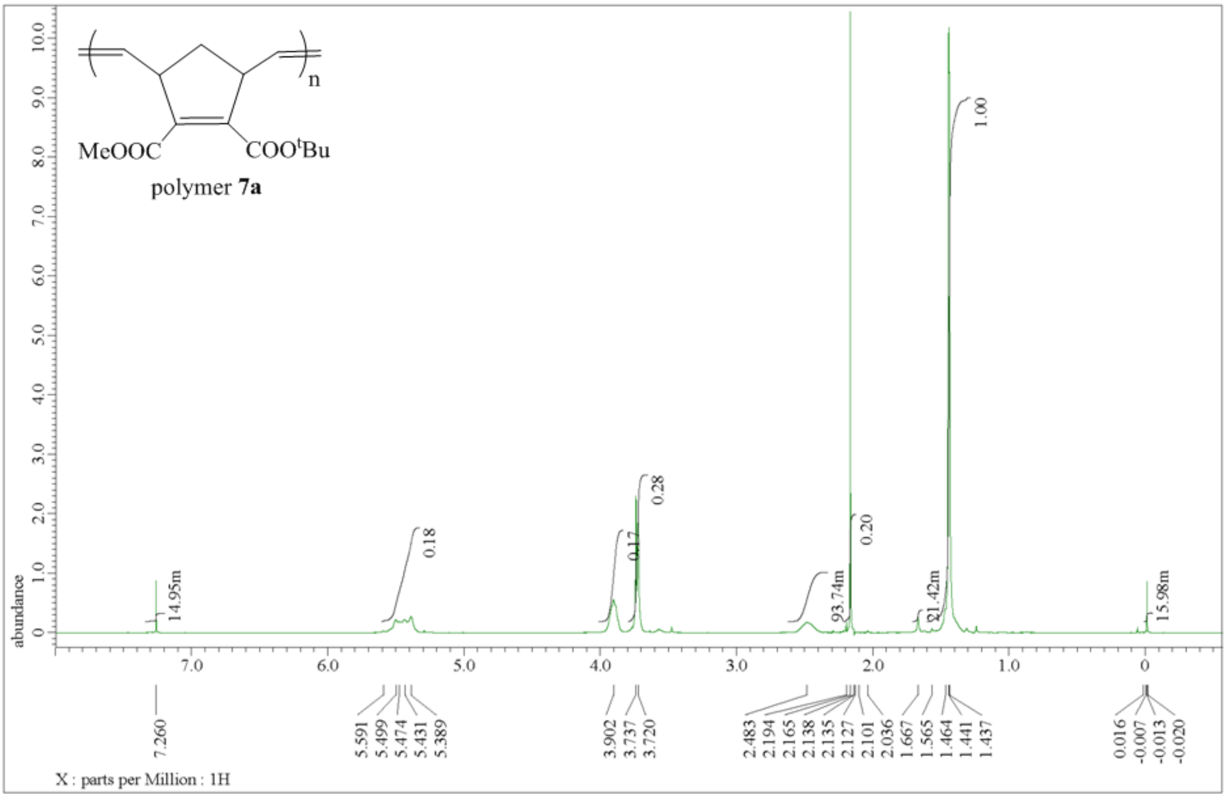
MeOOC
COO^{t}Bu
polymer **7a**
n
abundance
X : parts per Million : 1H

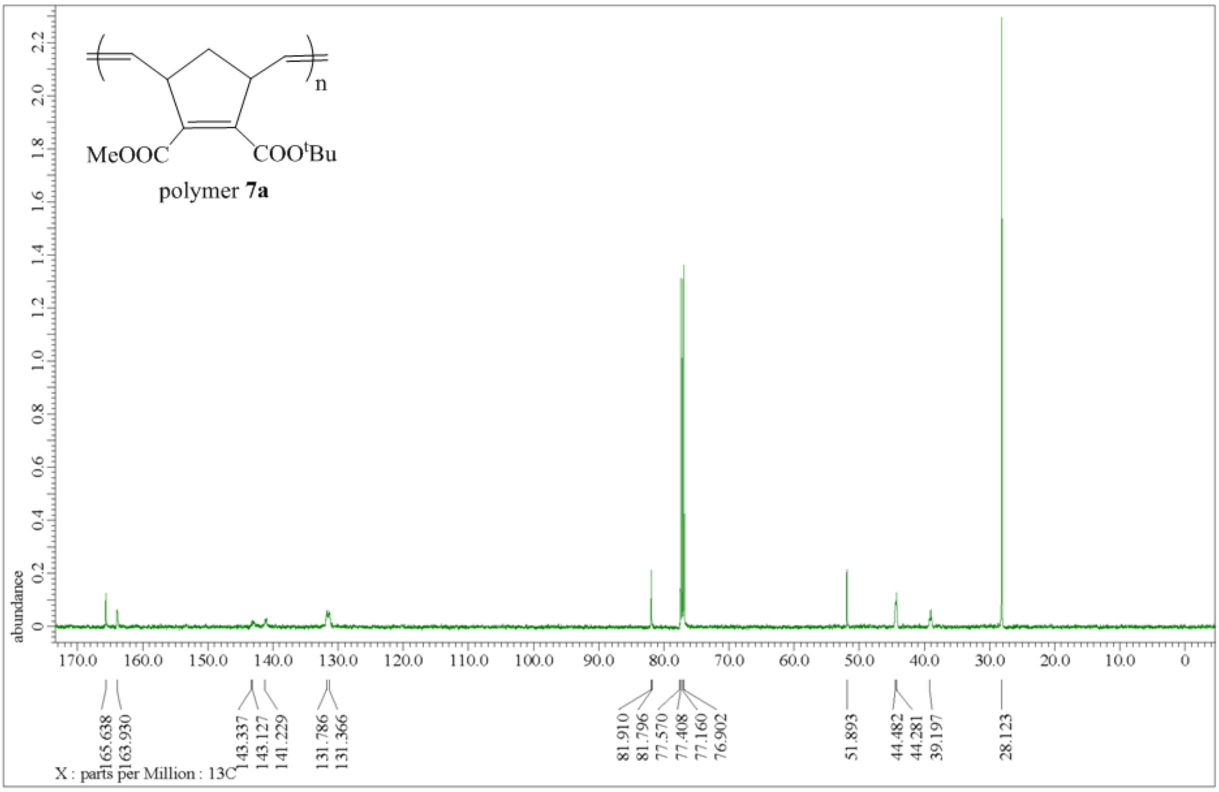
MeOOC
COO^{t}Bu
polymer **7a**
n
abundance
X : parts per Million : 13C

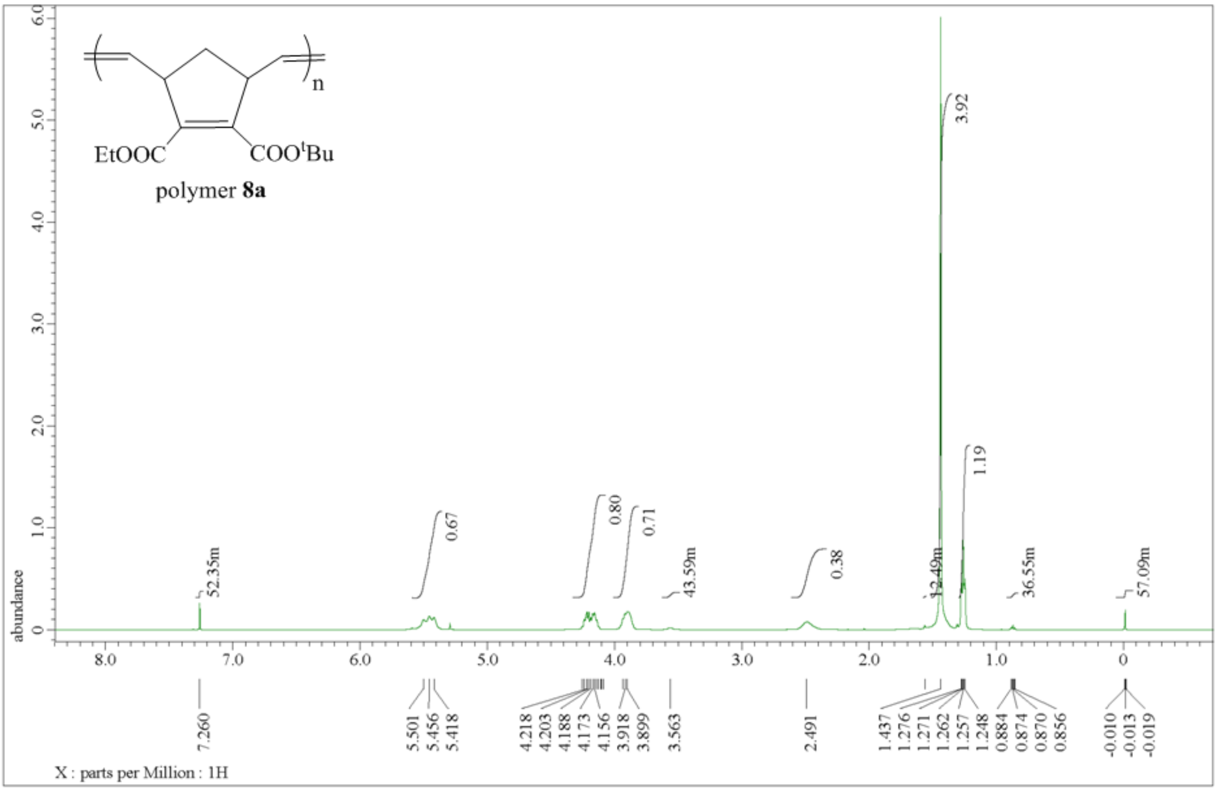
EtOOC
COO^{t}Bu
polymer **8a**
abundance
X : parts per Million : 1H
52.35m
0.67
0.80
0.71
43.59m
0.38
12.49m
3.92
1.19
36.55m
57.09m
7.260
5.501 5.456 5.418
4.218 4.203 4.188 4.173 4.156 3.918 3.899
3.563
2.491
1.437 1.276 1.271 1.262 1.257 1.248 0.884 0.874 0.870 0.856
-0.010 -0.013 -0.019

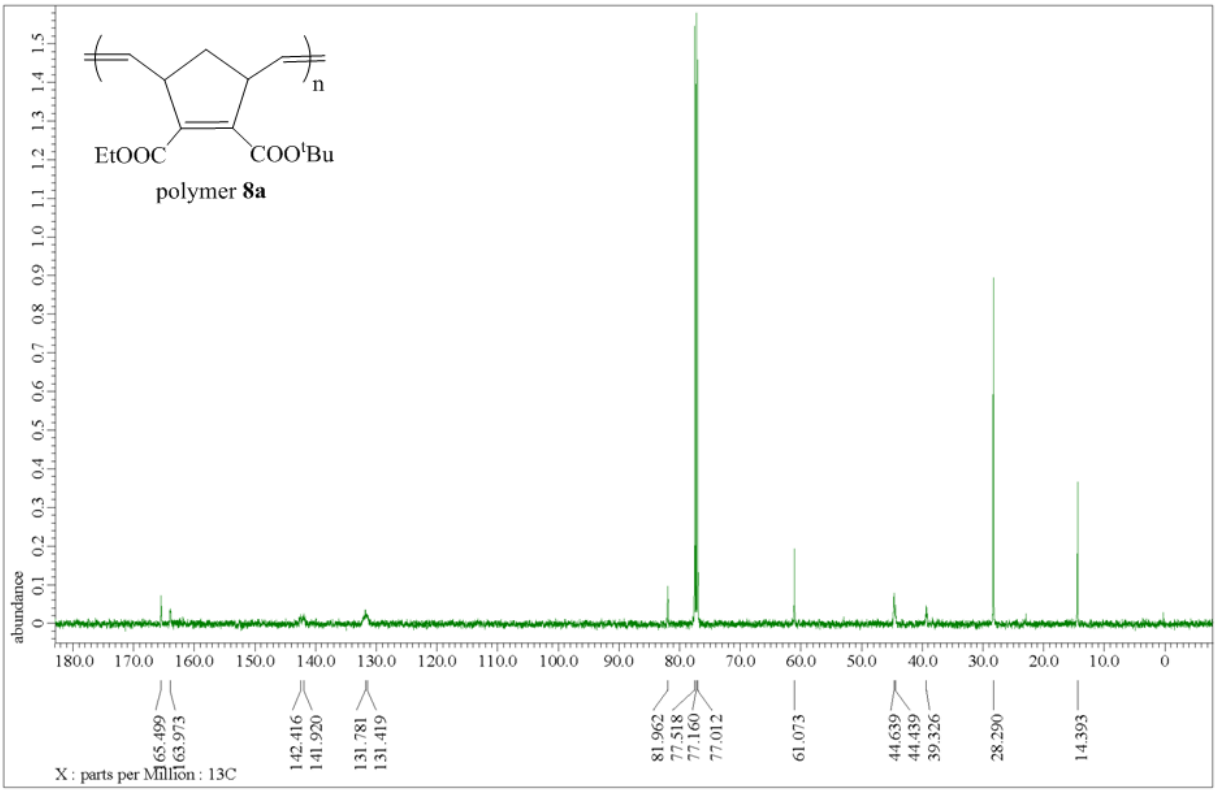
EtOOC
COO^{t}Bu
polymer **8a**
abundance
X : parts per Million : 13C
165.499 163.973
142.416 141.920
131.781 131.419
81.962 77.518 77.160 77.012
61.073
44.639 44.439 39.326
28.290
14.393

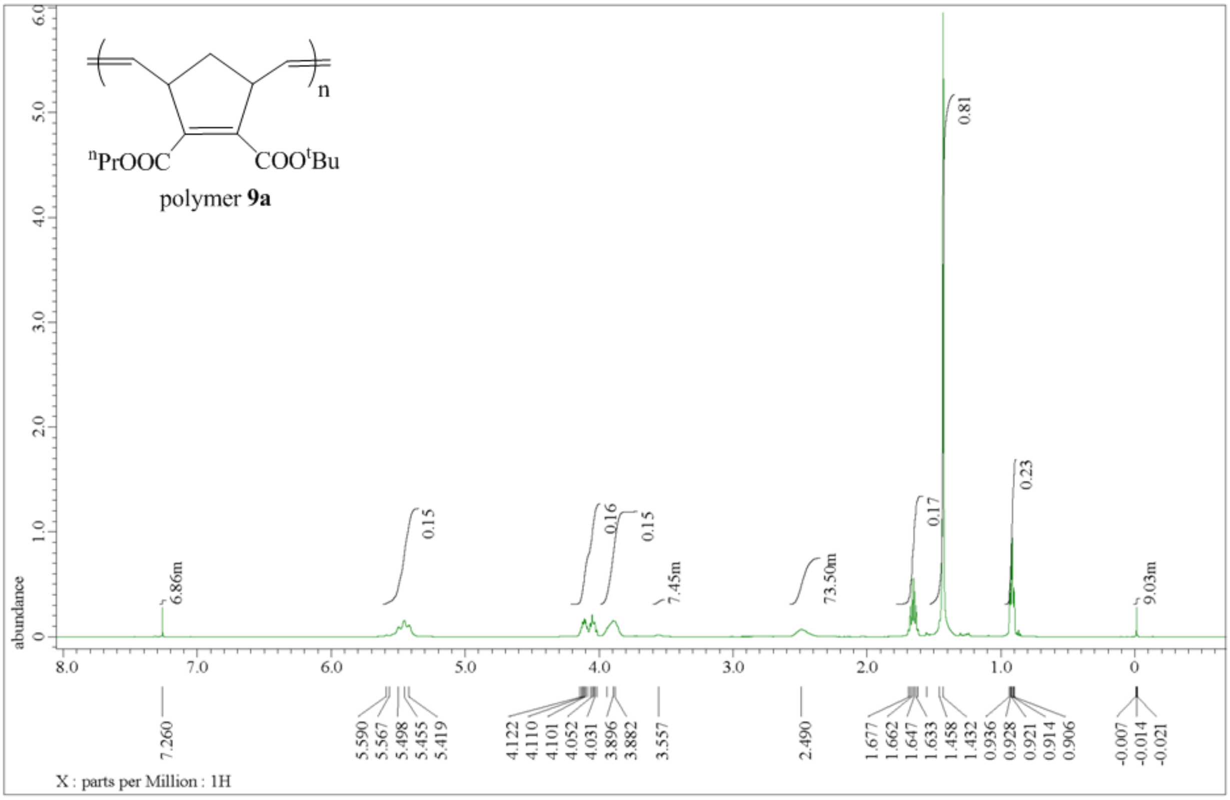

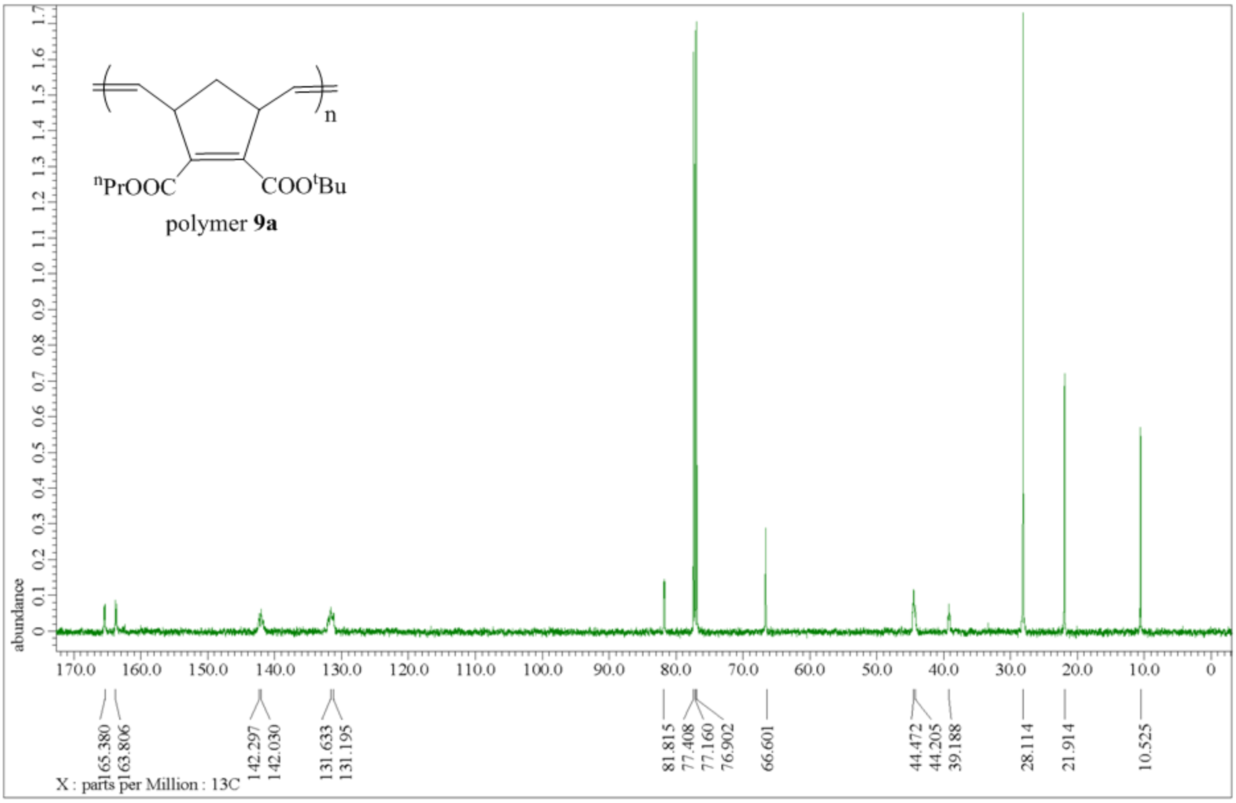

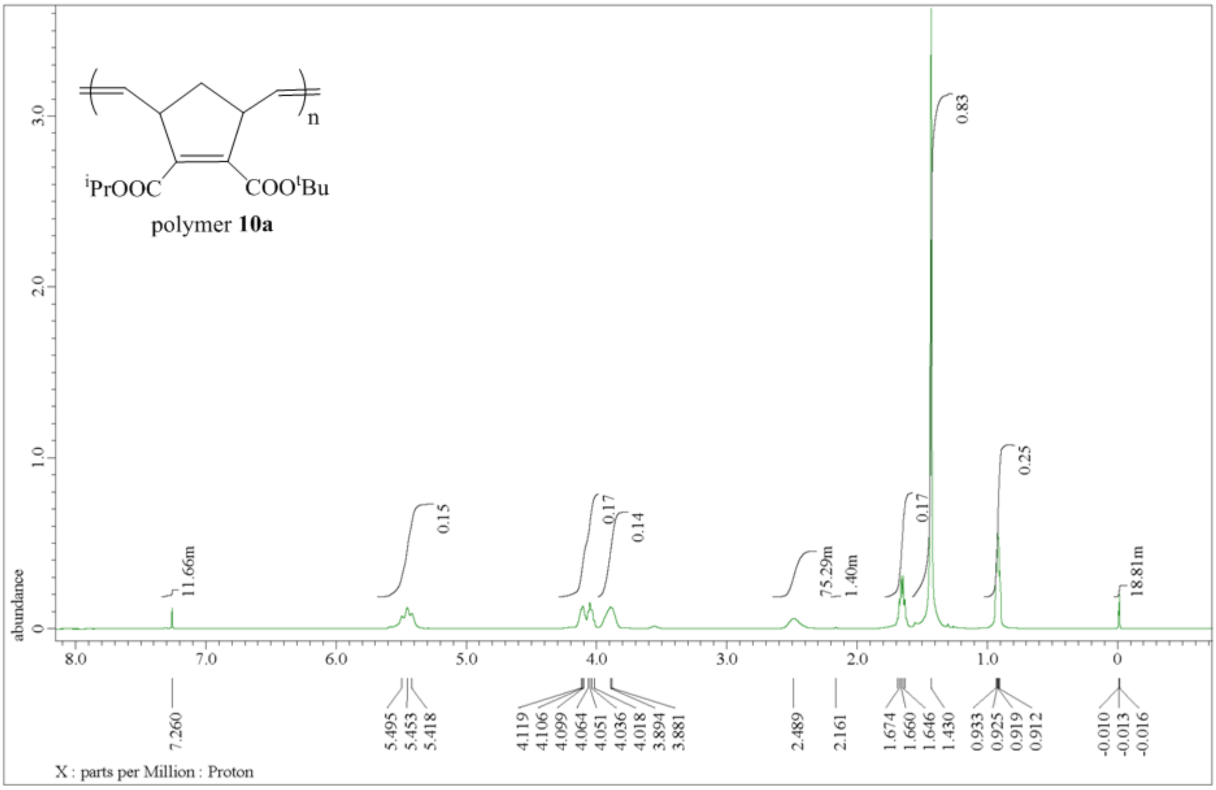

iPrOOC
COOtBu
n
polymer 10a
abundance
X : parts per Million : Proton
11.66m
0.15
0.17
0.14
75.29m
1.40m
0.17
0.83
0.25
18.81m
7.260
5.495
5.453
5.418
4.119
4.106
4.099
4.064
4.051
4.036
4.018
3.894
3.881
2.489
2.161
1.674
1.660
1.646
1.430
0.933
0.925
0.919
0.912
-0.010
-0.013
-0.016

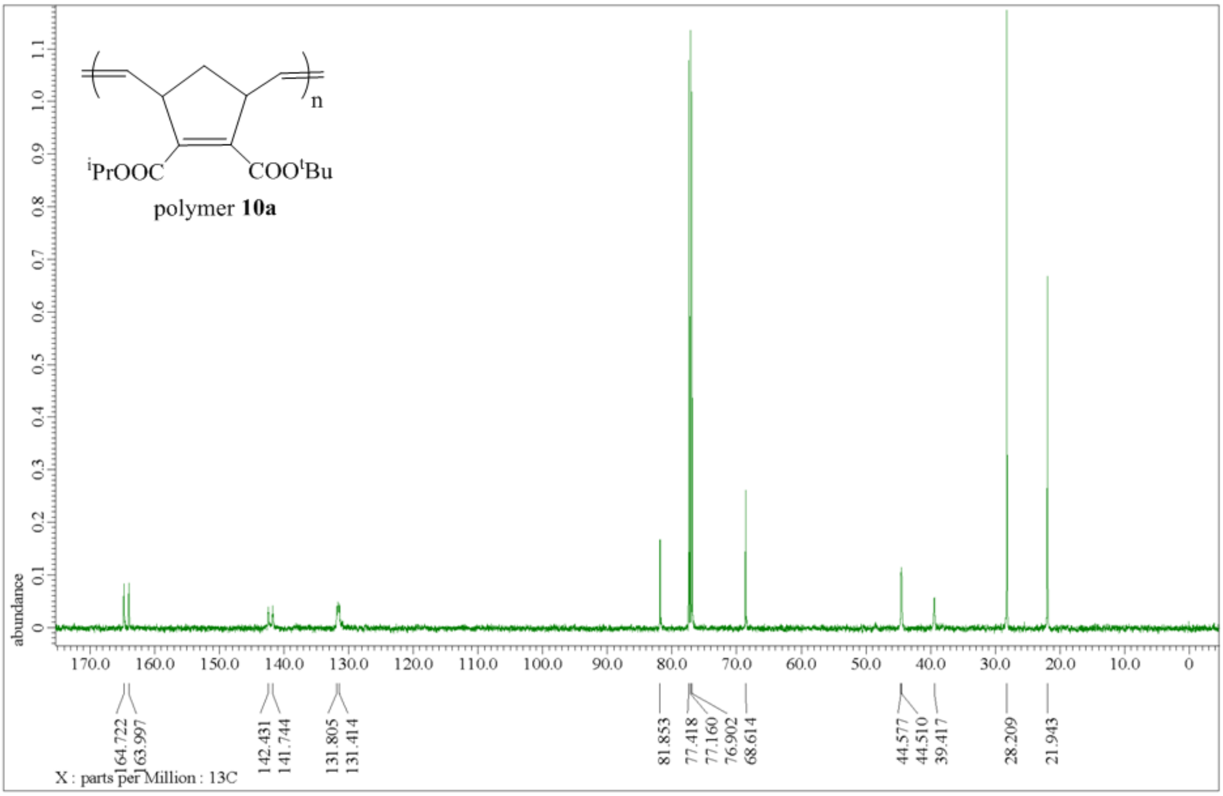

iPrOOC
COOtBu
n
polymer 10a
abundance
X : parts per Million : 13C
164.722
163.997
142.431
141.744
131.805
131.414
81.853
77.418
77.160
76.902
68.614
44.577
44.510
39.417
28.209
21.943

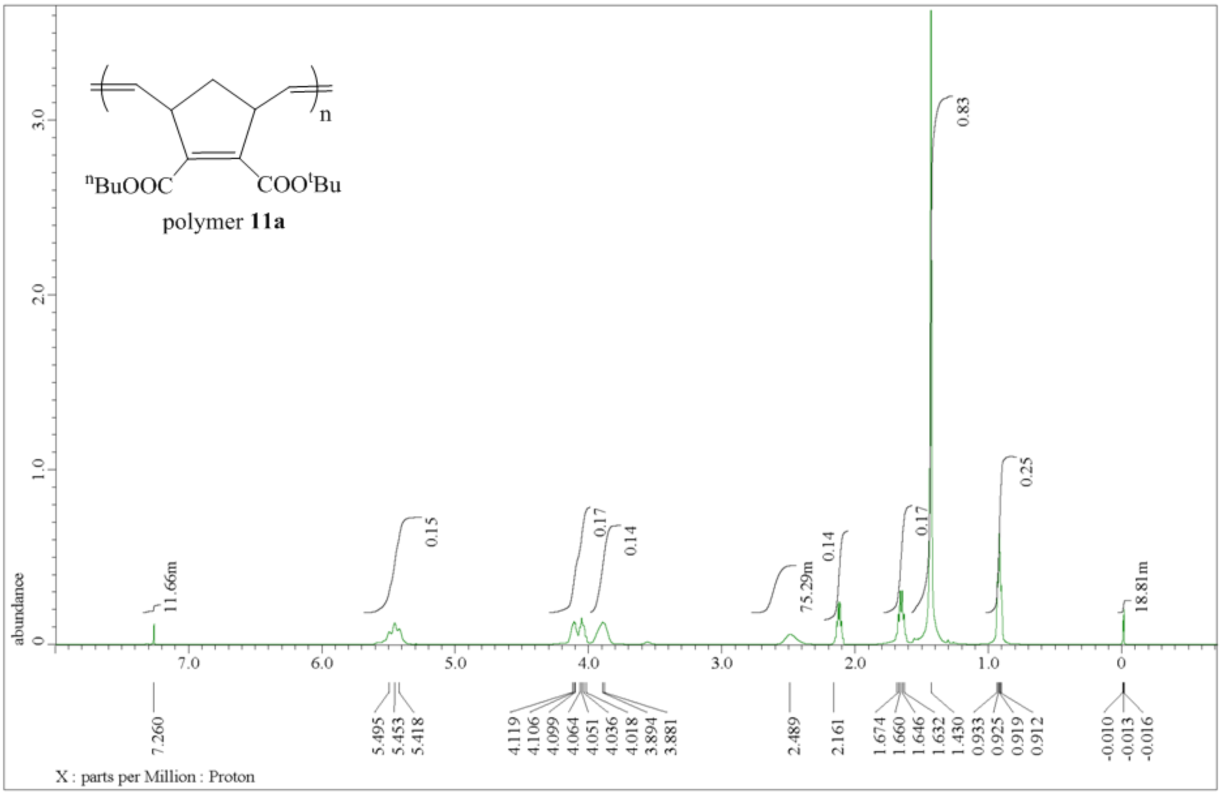
polymer 11a
nBuOOC
COOtBu
n
abundance
X : parts per Million : Proton
11.66m
0.15
0.17
0.14
75.29m
0.14
0.17
0.83
0.25
18.81m
7.260
5.495
5.453
5.418
4.119
4.106
4.099
4.064
4.051
4.036
4.018
3.894
3.881
2.489
2.161
1.674
1.660
1.646
1.632
1.430
0.933
0.925
0.919
0.912
-0.010
-0.013
-0.016

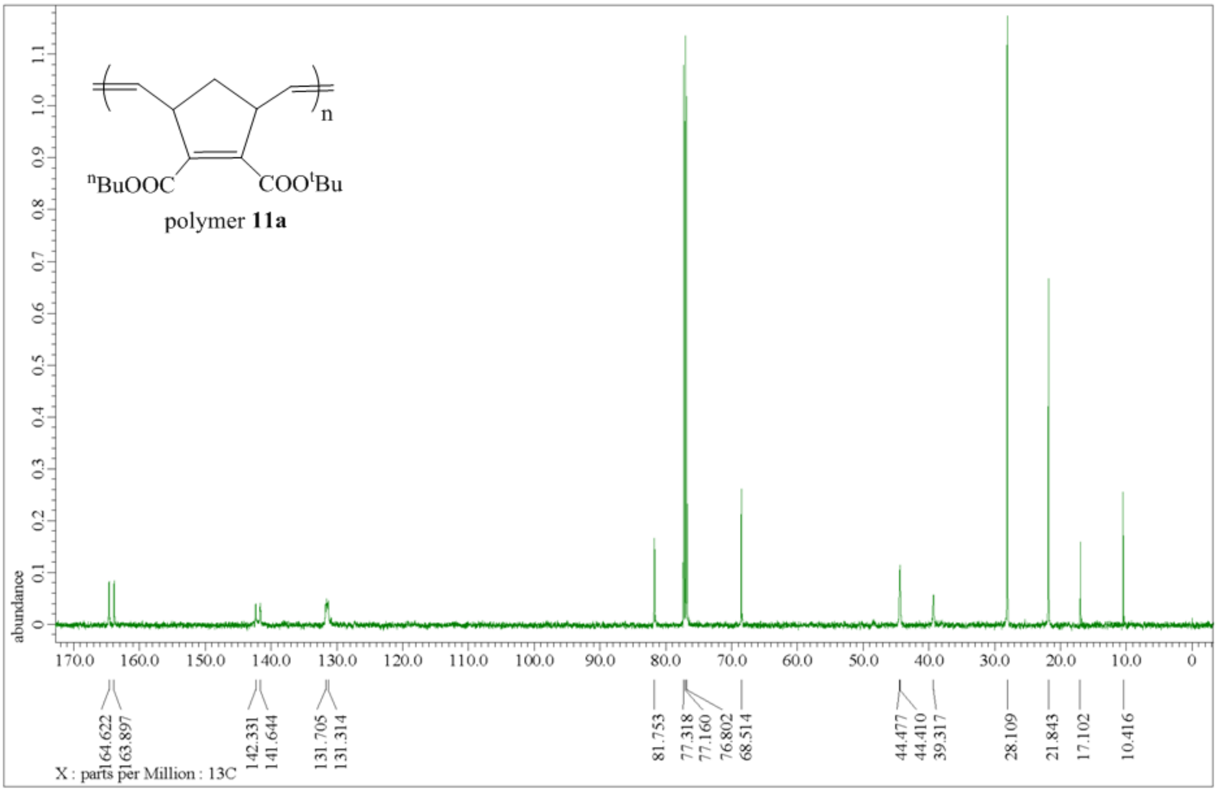
polymer 11a
nBuOOC
COOtBu
n
abundance
X : parts per Million : 13C
164.622
163.897
142.331
141.644
131.705
131.314
81.753
77.318
77.160
76.802
68.514
44.477
44.410
39.317
28.109
21.843
17.102
10.416

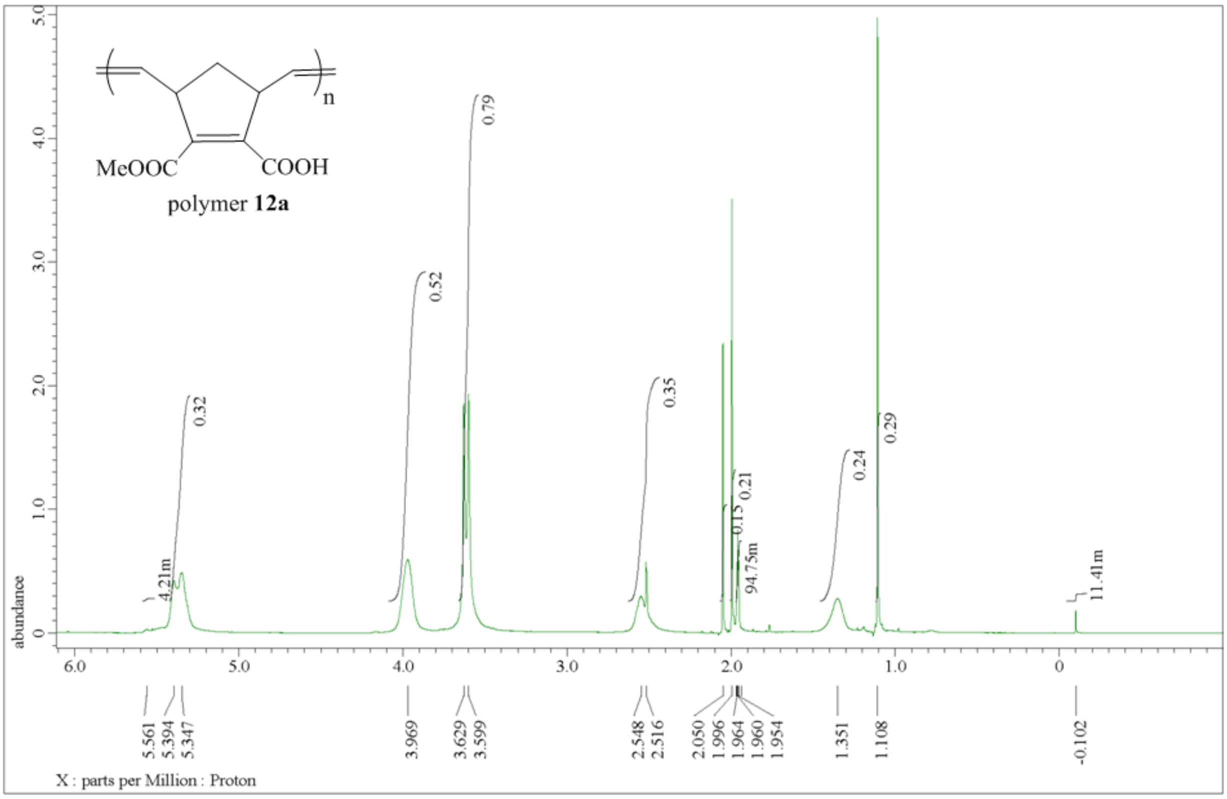

MeOOC
COOH
n
polymer 12a
abundance
X : parts per Million : Proton

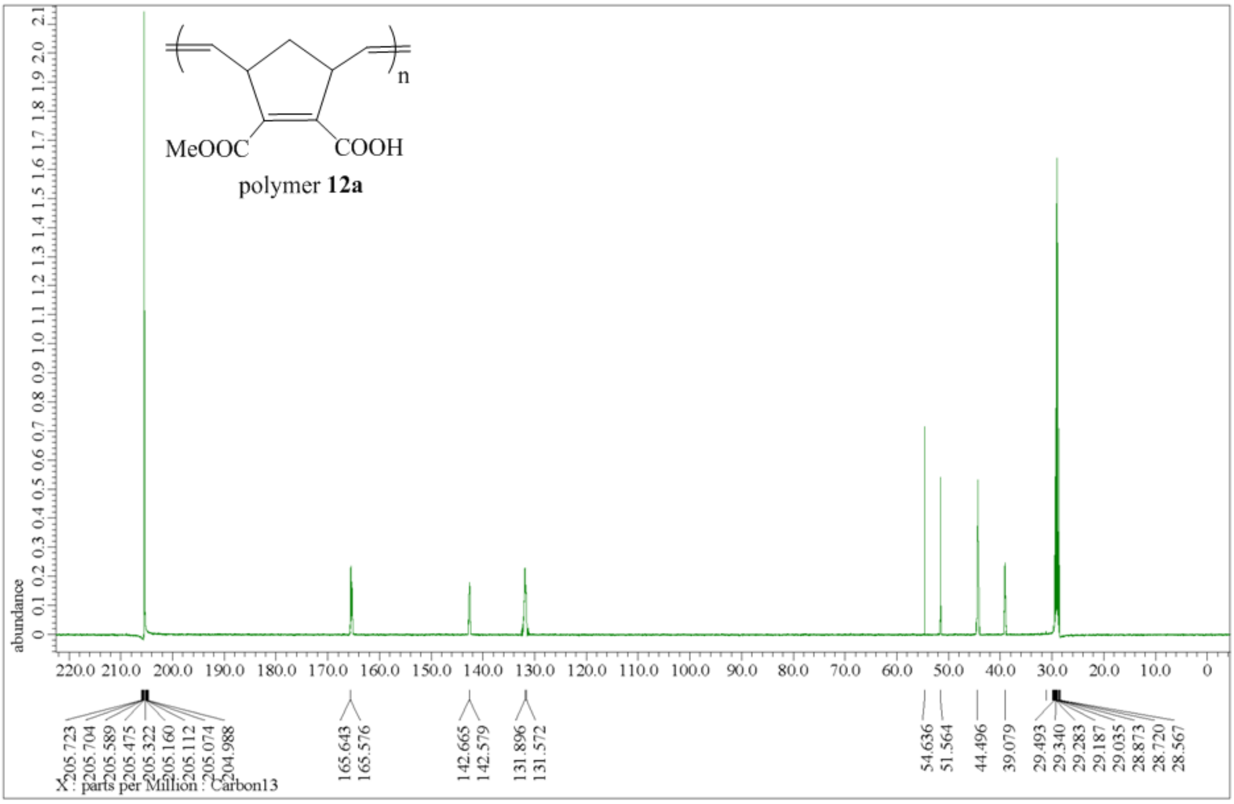

MeOOC
COOH
n
polymer 12a
abundance
X : parts per Million : Carbon13

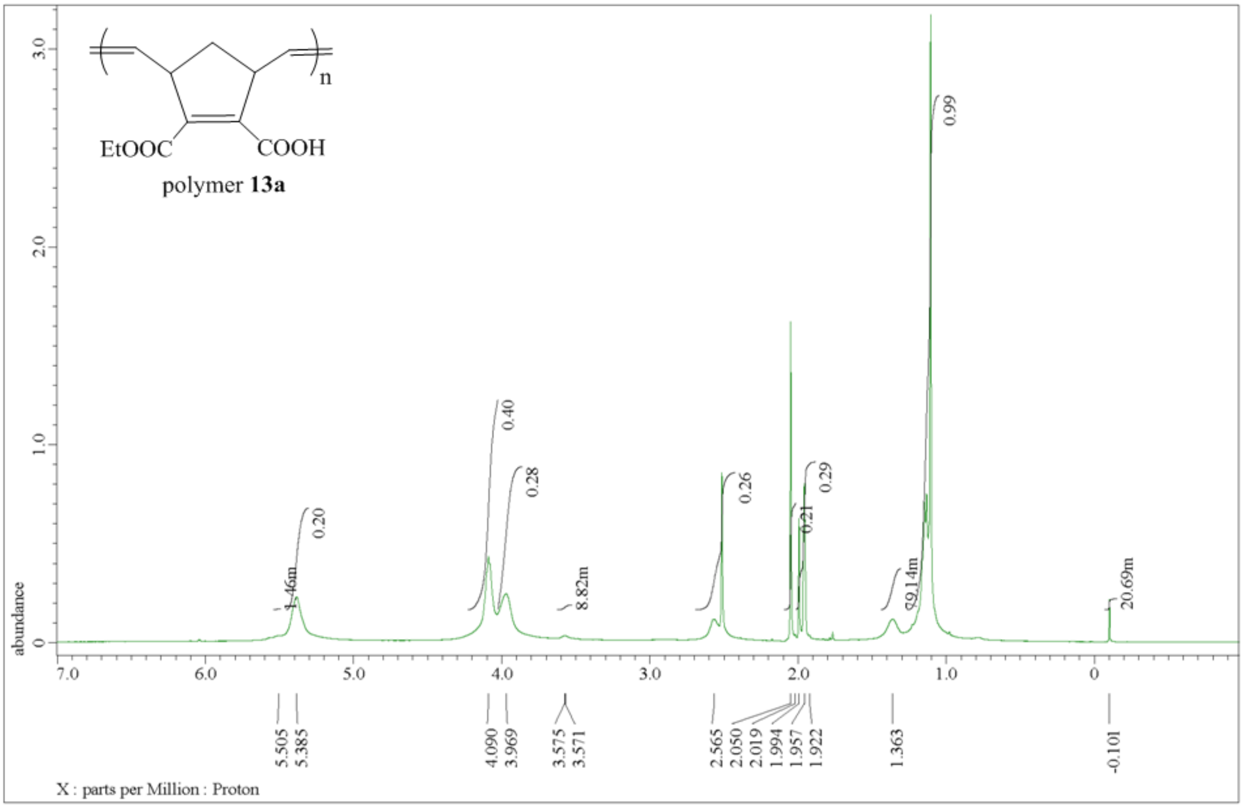

EtOOC
COOH
polymer **13a**
abundance
X : parts per Million : Proton
5.505
5.385
4.090
3.969
3.575
3.571
2.565
2.050
2.019
1.994
1.957
1.922
1.363
-0.101
0.20
0.40
0.28
0.26
0.21
0.29
0.99
1.46m
8.82m
79.14m
20.69m

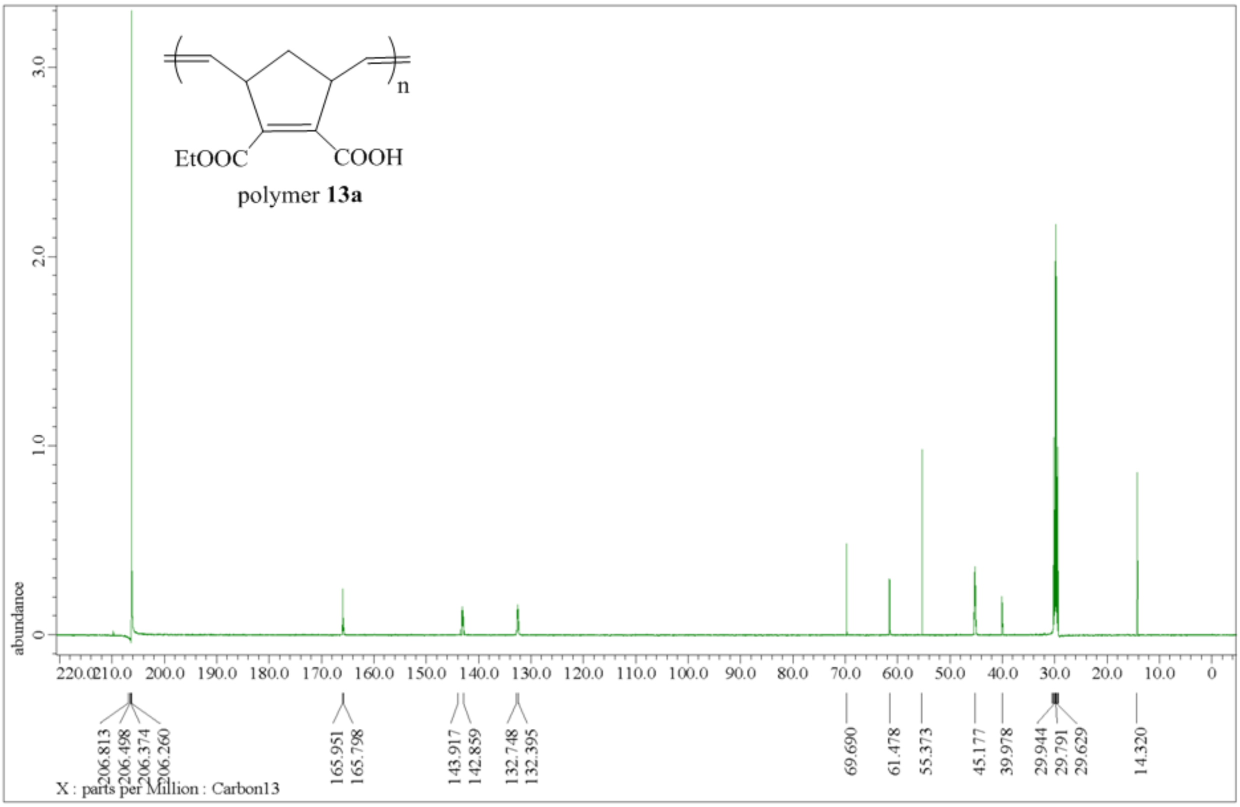

EtOOC
COOH
polymer **13a**
abundance
X : parts per Million : Carbon13
206.813
206.498
206.374
206.260
165.951
165.798
143.917
142.859
132.748
132.395
69.690
61.478
55.373
45.177
39.978
29.944
29.791
29.629
14.320

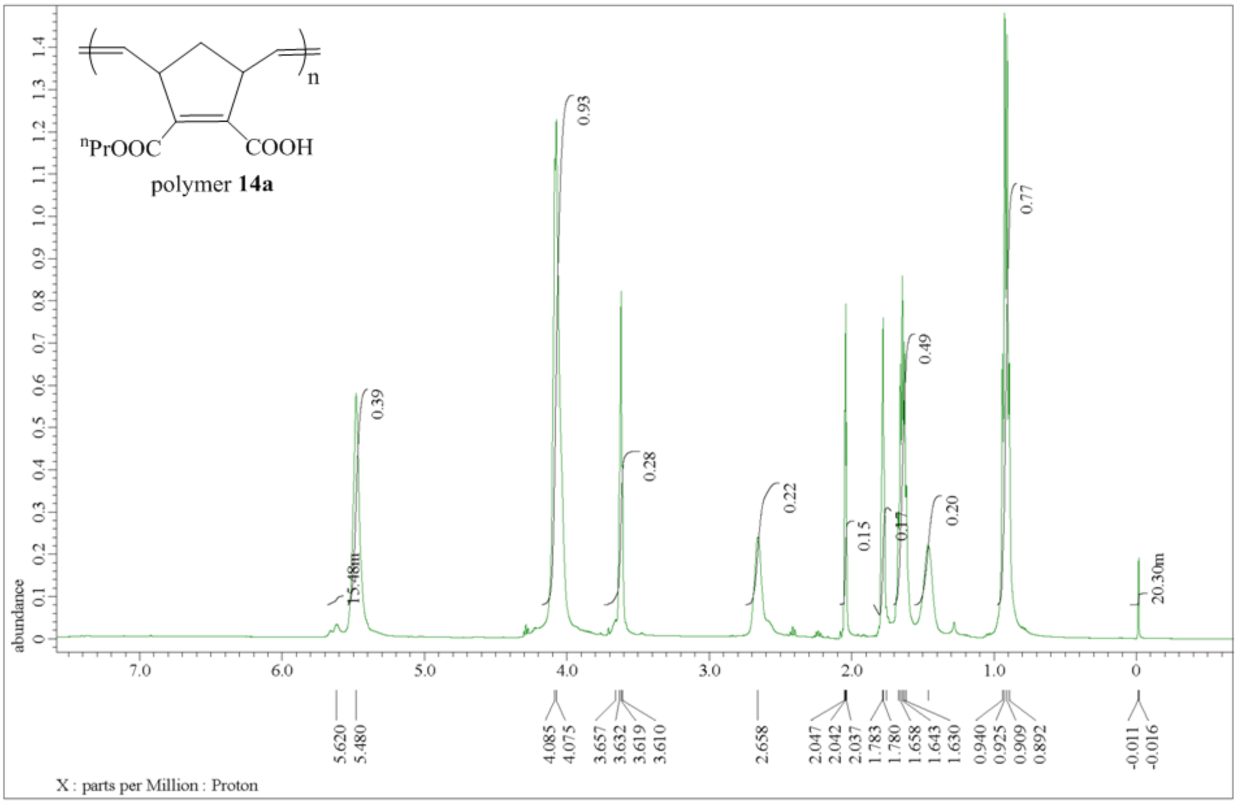

^{n}PrOOC
COOH
n
polymer **14a**
abundance
X : parts per Million : Proton

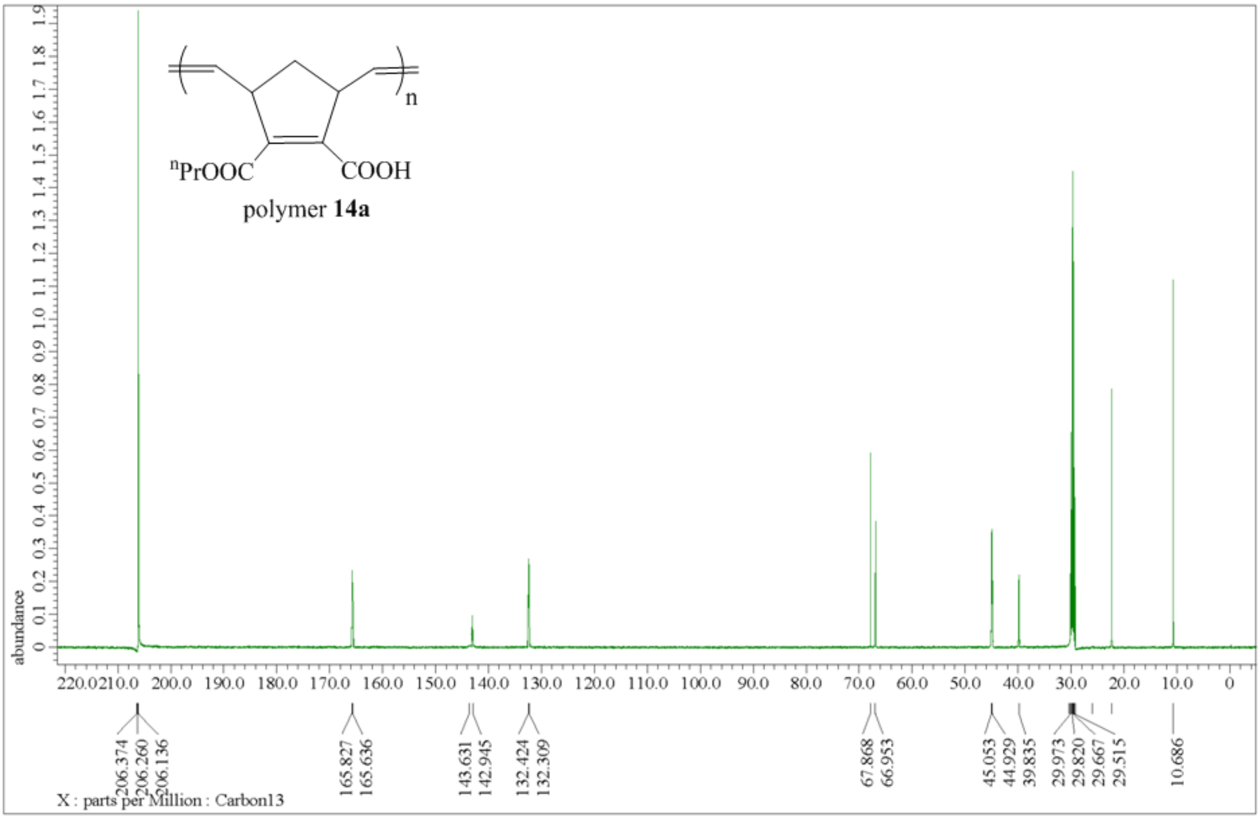

^{n}PrOOC
COOH
n
polymer **14a**
abundance
X : parts per Million : Carbon13

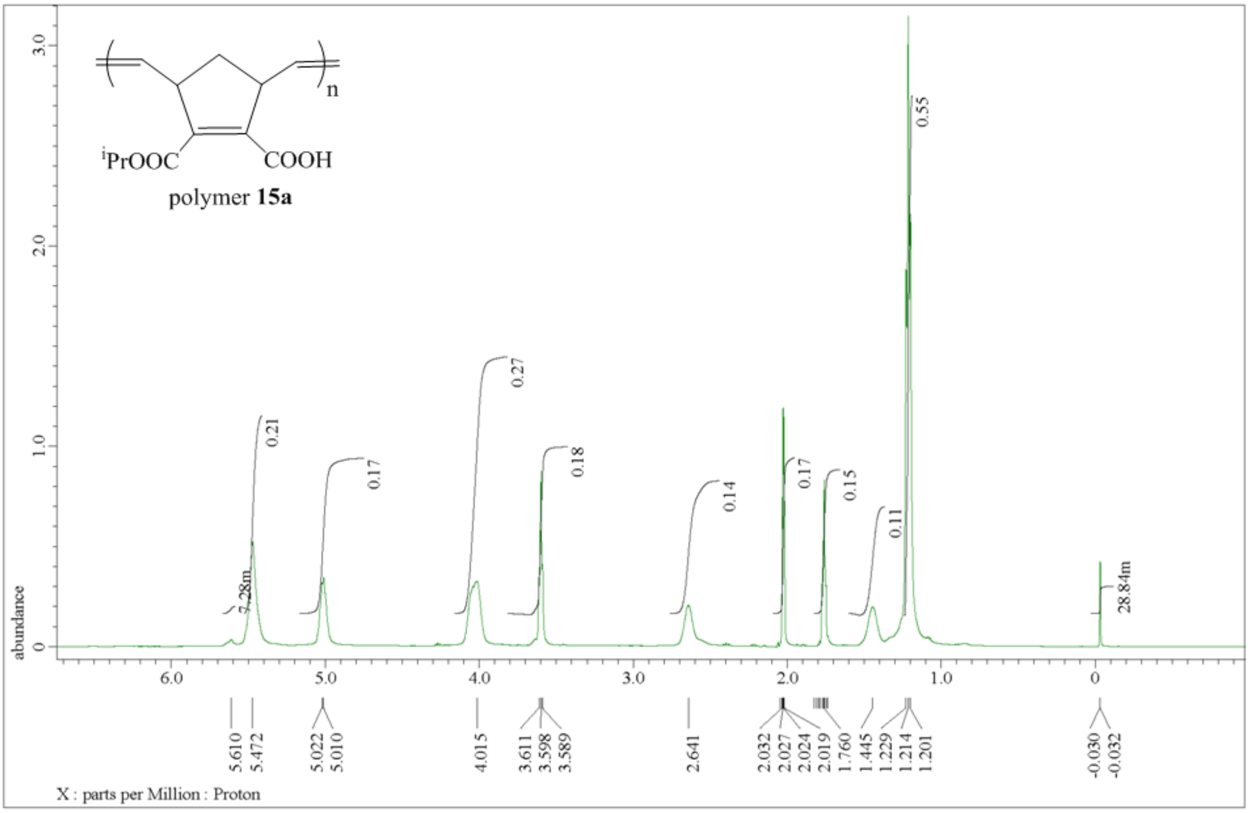

polymer **15a**
^{i}PrOOC
COOH
n
abundance
X : parts per Million : Proton
5.610
5.472
5.022
5.010
4.015
3.611
3.598
3.589
2.641
2.032
2.027
2.024
2.019
1.760
1.445
1.229
1.214
1.201
-0.030
-0.032
0.21
0.17
0.27
0.18
0.14
0.17
0.15
0.11
0.55
28.84m

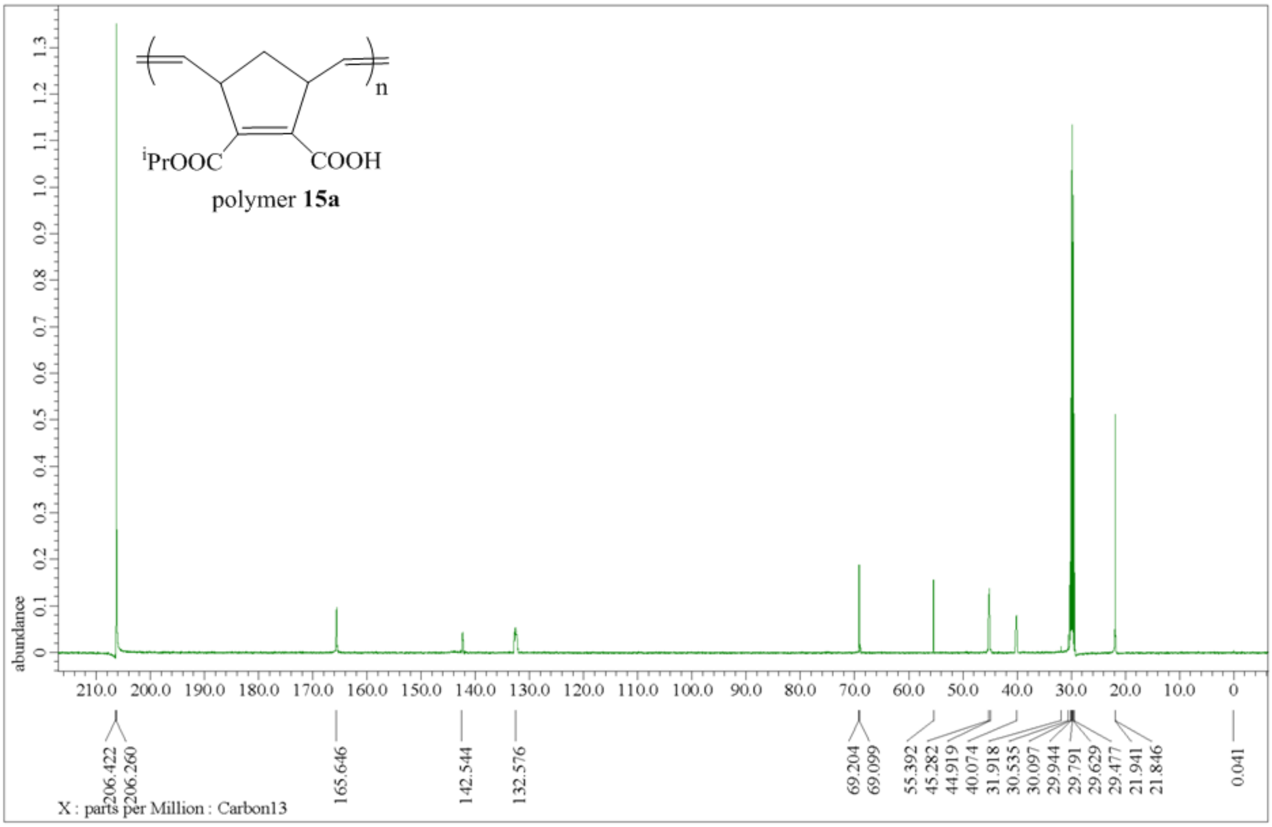

polymer **15a**
^{i}PrOOC
COOH
n
abundance
X : parts per Million : Carbon13
206.422
206.260
165.646
142.544
132.576
69.204
69.099
55.392
45.282
44.919
40.074
31.918
30.535
30.097
29.944
29.791
29.629
29.477
21.941
21.846
0.041

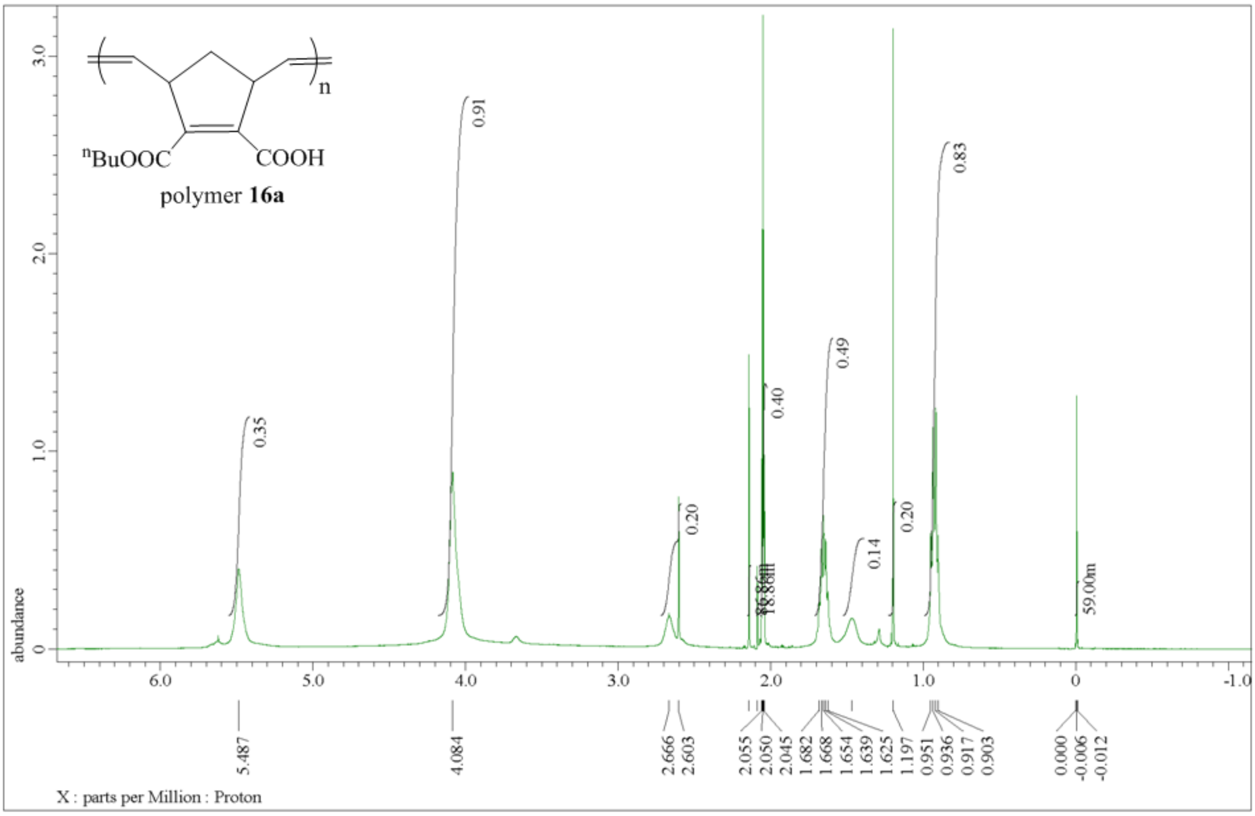

ⁿBuOOC
COOH
polymer 16a
abundance
X : parts per Million : Proton
5.487
4.084
2.666
2.603
2.055
2.050
2.045
1.682
1.668
1.654
1.639
1.625
1.197
0.951
0.936
0.917
0.903
0.000
-0.006
-0.012
0.35
0.91
0.20
0.40
0.49
0.14
0.20
0.83
59.00m

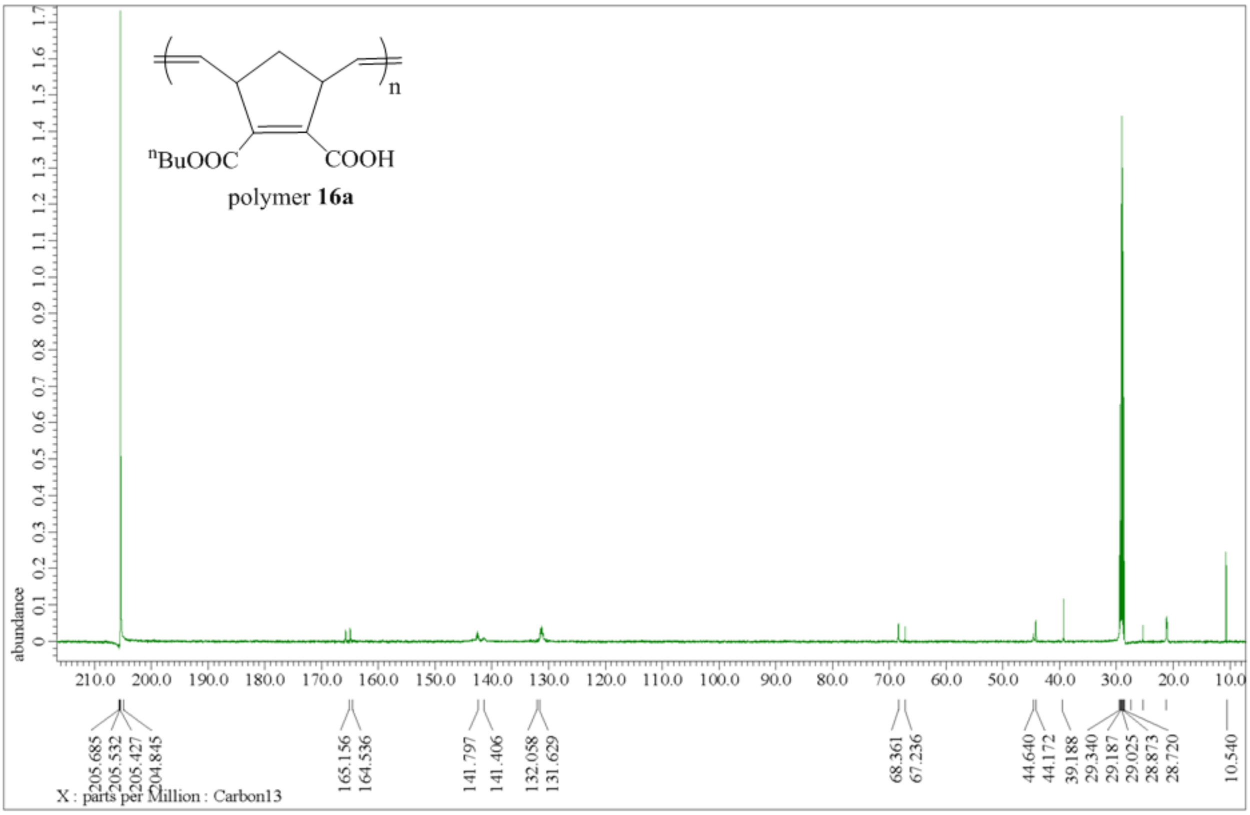

ⁿBuOOC
COOH
polymer 16a
abundance
X : parts per Million : Carbon13
205.685
205.532
205.427
204.845
165.156
164.536
141.797
141.406
132.058
131.629
68.361
67.236
44.640
44.172
39.188
29.340
29.187
29.025
28.873
28.720
10.540

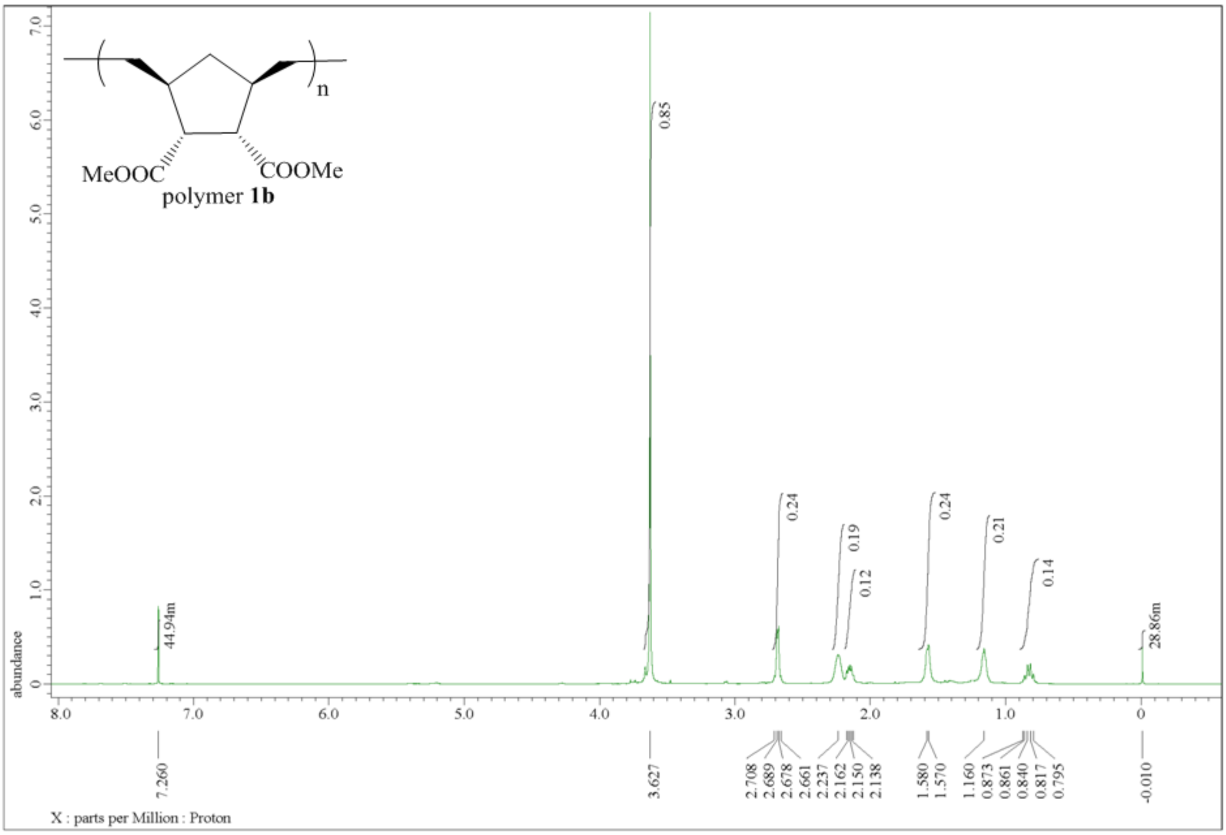
MeOOC
COOMe
n
polymer 1b
abundance
0.85
0.24
0.19
0.12
0.24
0.21
0.14
44.94m
28.86m
7.260
3.627
2.708
2.689
2.678
2.661
2.237
2.162
2.150
2.138
1.580
1.570
1.160
0.873
0.861
0.840
0.817
0.795
-0.010
X : parts per Million : Proton

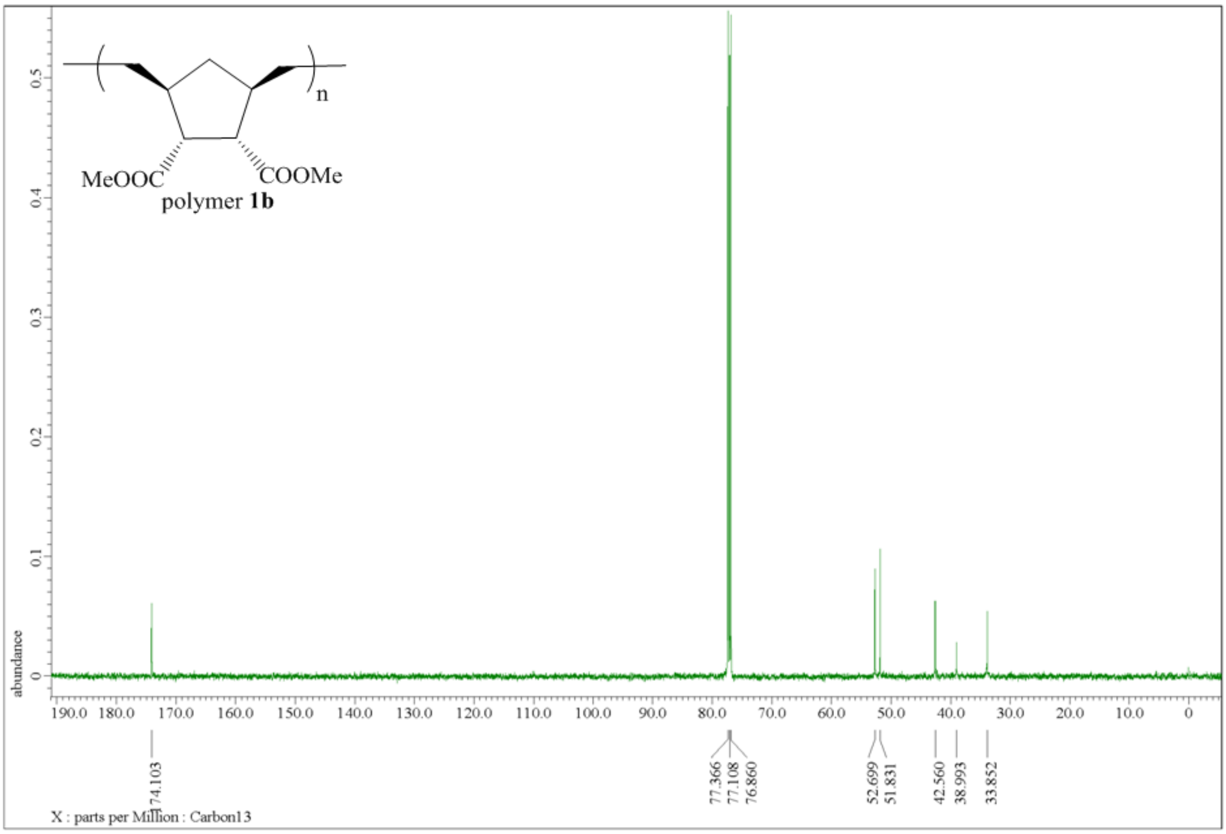
MeOOC
COOMe
n
polymer 1b
abundance
174.103
77.366
77.108
76.860
52.699
51.831
42.560
38.993
33.852
X : parts per Million : Carbon13

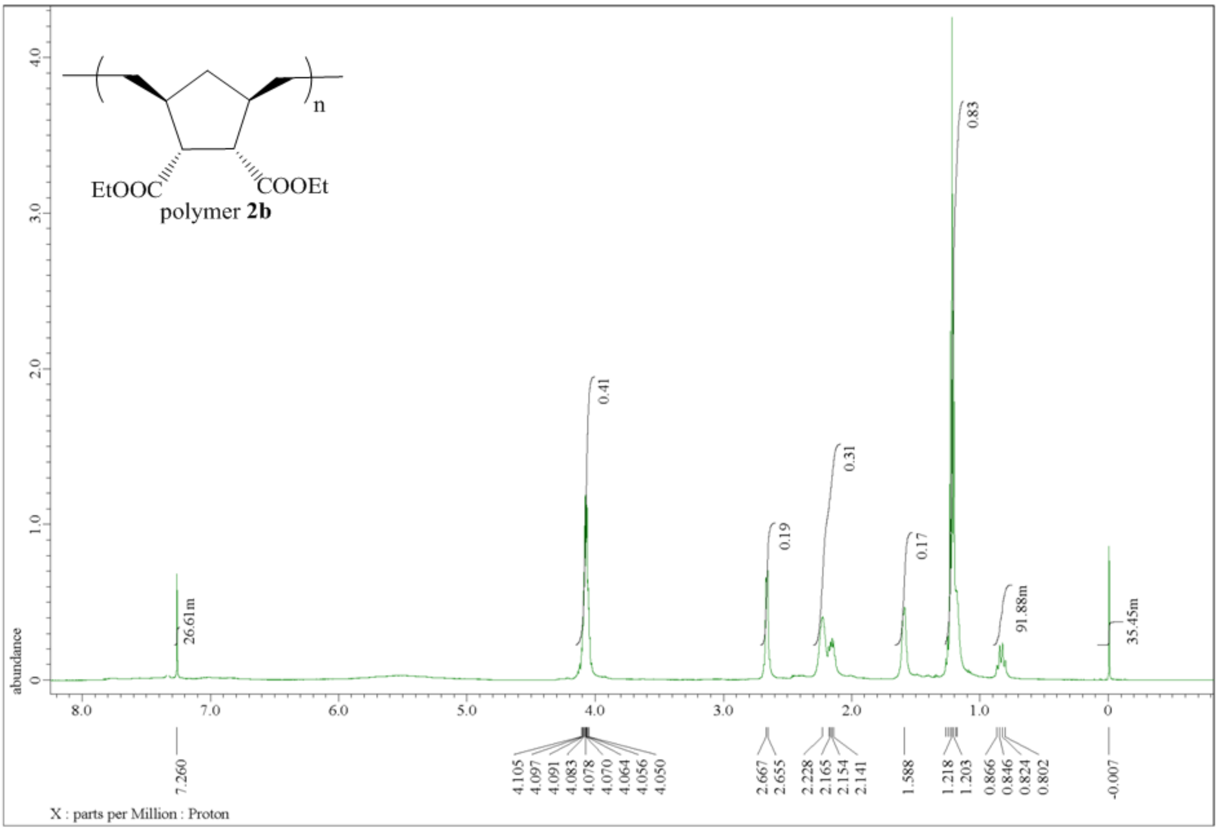
EtOOC
COOEt
polymer 2b
abundance
X : parts per Million : Proton

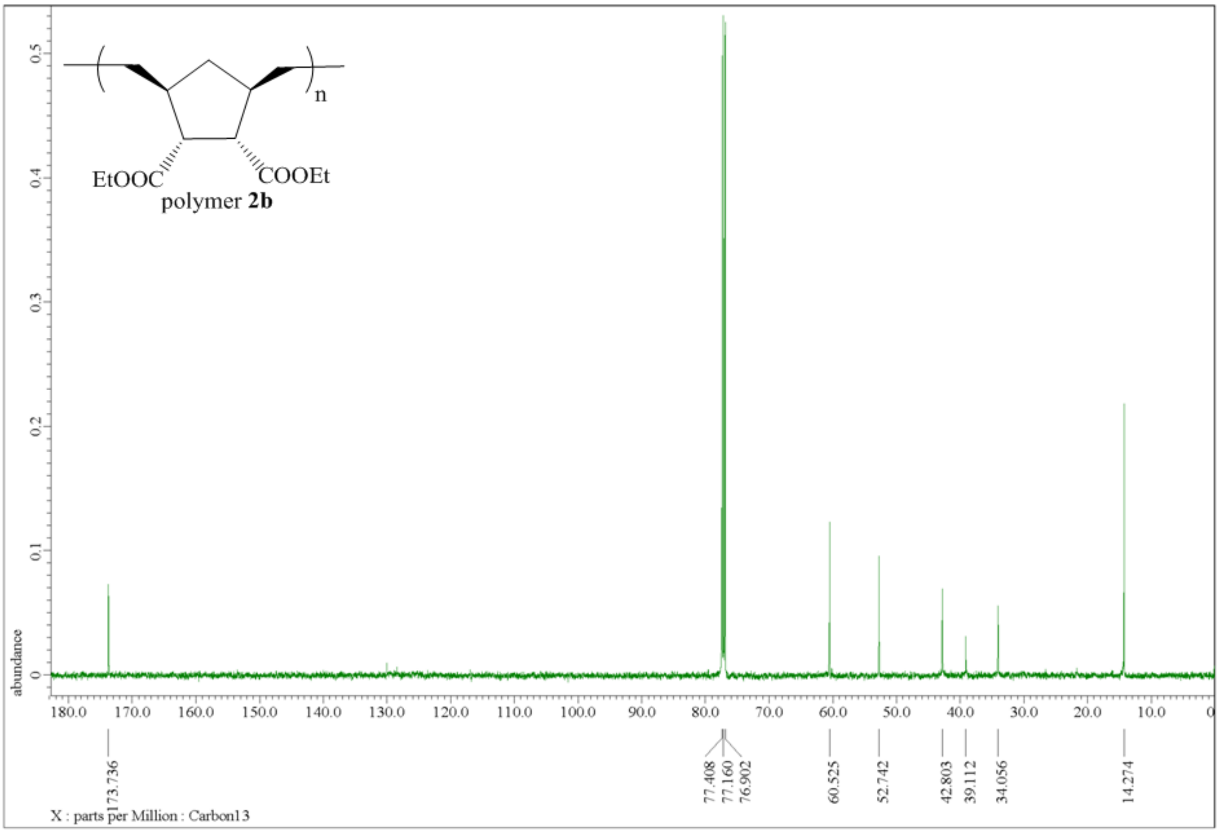
EtOOC
COOEt
polymer 2b
abundance
X : parts per Million : Carbon13

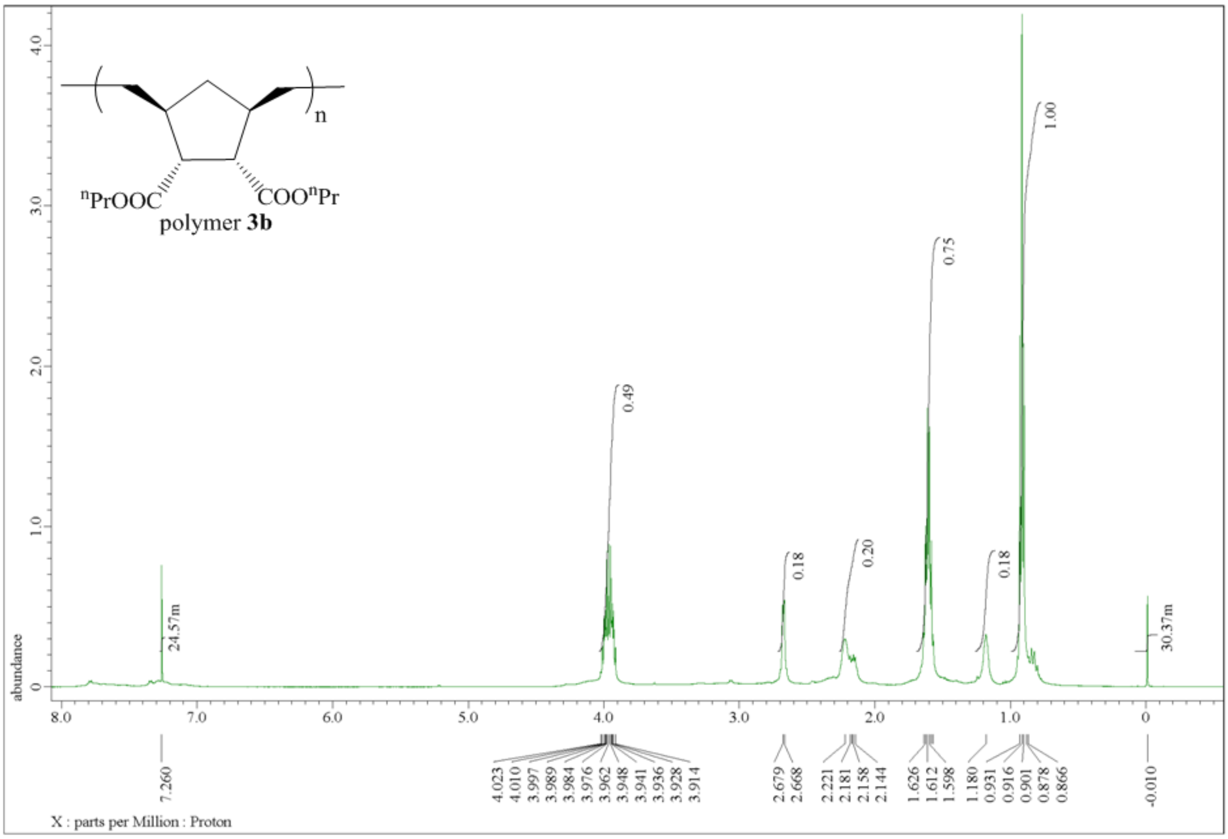

polymer 3b
^{n}PrOOC
COO^{n}Pr
n
24.57m
0.49
0.18
0.20
0.75
0.18
1.00
30.37m
7.260
4.023
4.010
3.997
3.989
3.984
3.976
3.962
3.948
3.941
3.936
3.928
3.914
2.679
2.668
2.221
2.181
2.158
2.144
1.626
1.612
1.598
1.180
0.931
0.916
0.901
0.878
0.866
-0.010
abundance
X : parts per Million : Proton

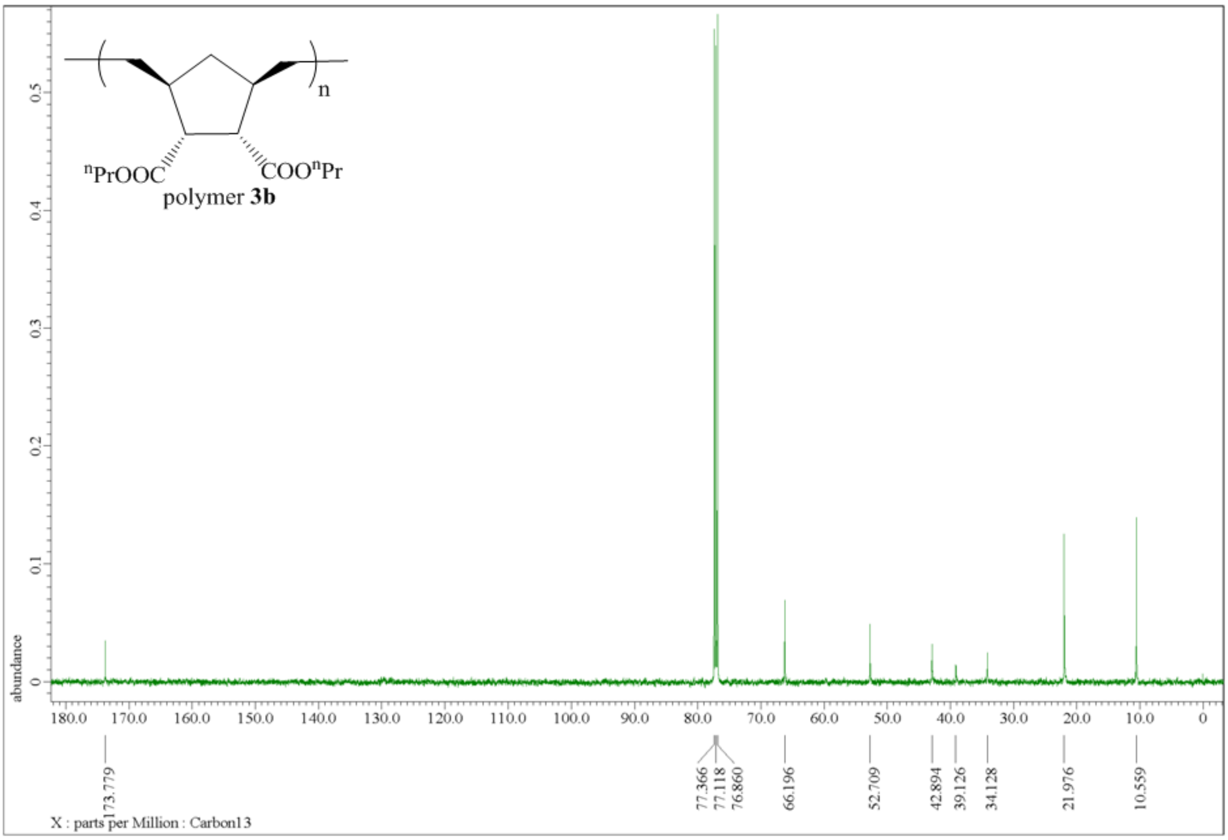

polymer 3b
^{n}PrOOC
COO^{n}Pr
n
173.779
77.366
77.118
76.860
66.196
52.709
42.894
39.126
34.128
21.976
10.559
abundance
X : parts per Million : Carbon13

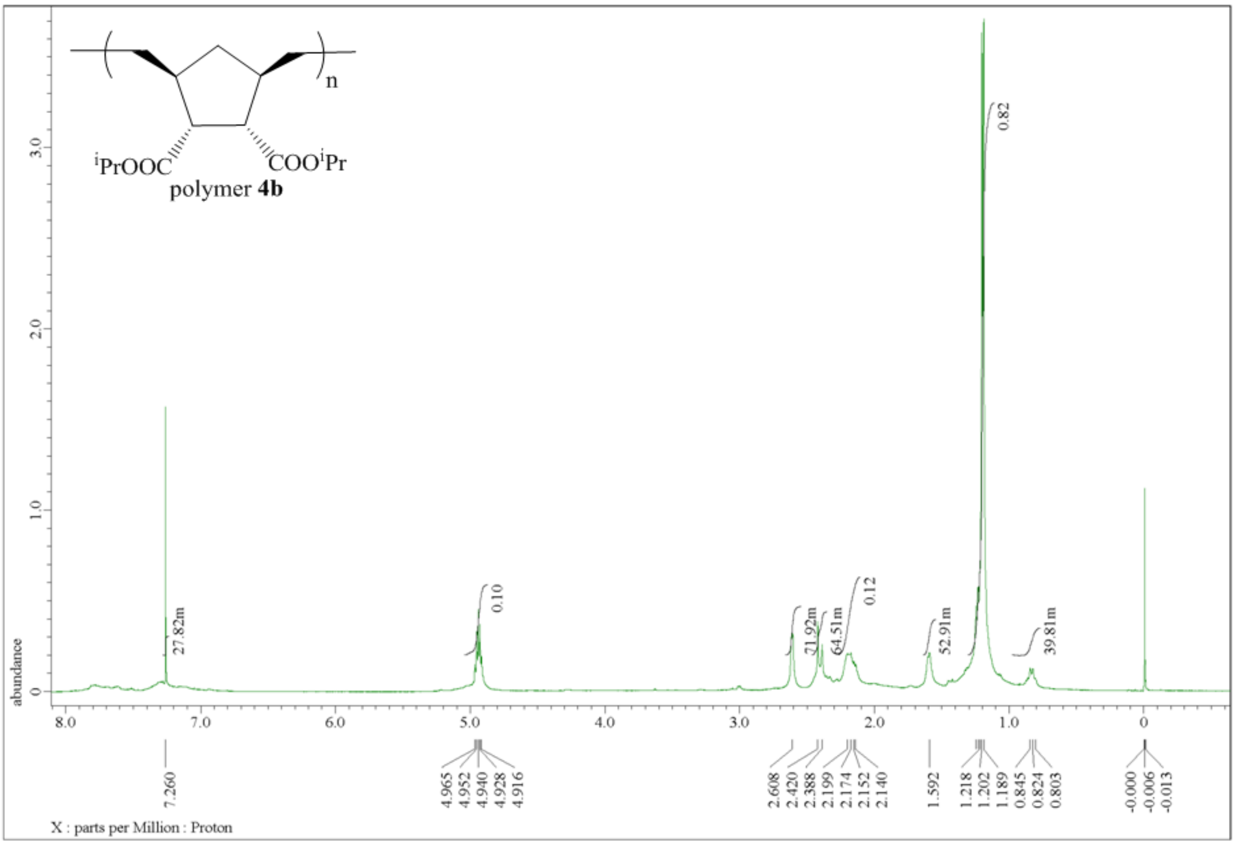

iPrOOC
COOiPr
n
polymer 4b
abundance
X : parts per Million : Proton
27.82m
0.10
71.92m
64.51m
0.12
52.91m
0.82
39.81m
7.260
4.965
4.952
4.940
4.928
4.916
2.608
2.420
2.388
2.199
2.174
2.152
2.140
1.592
1.218
1.202
1.189
0.845
0.824
0.803
-0.000
-0.006
-0.013

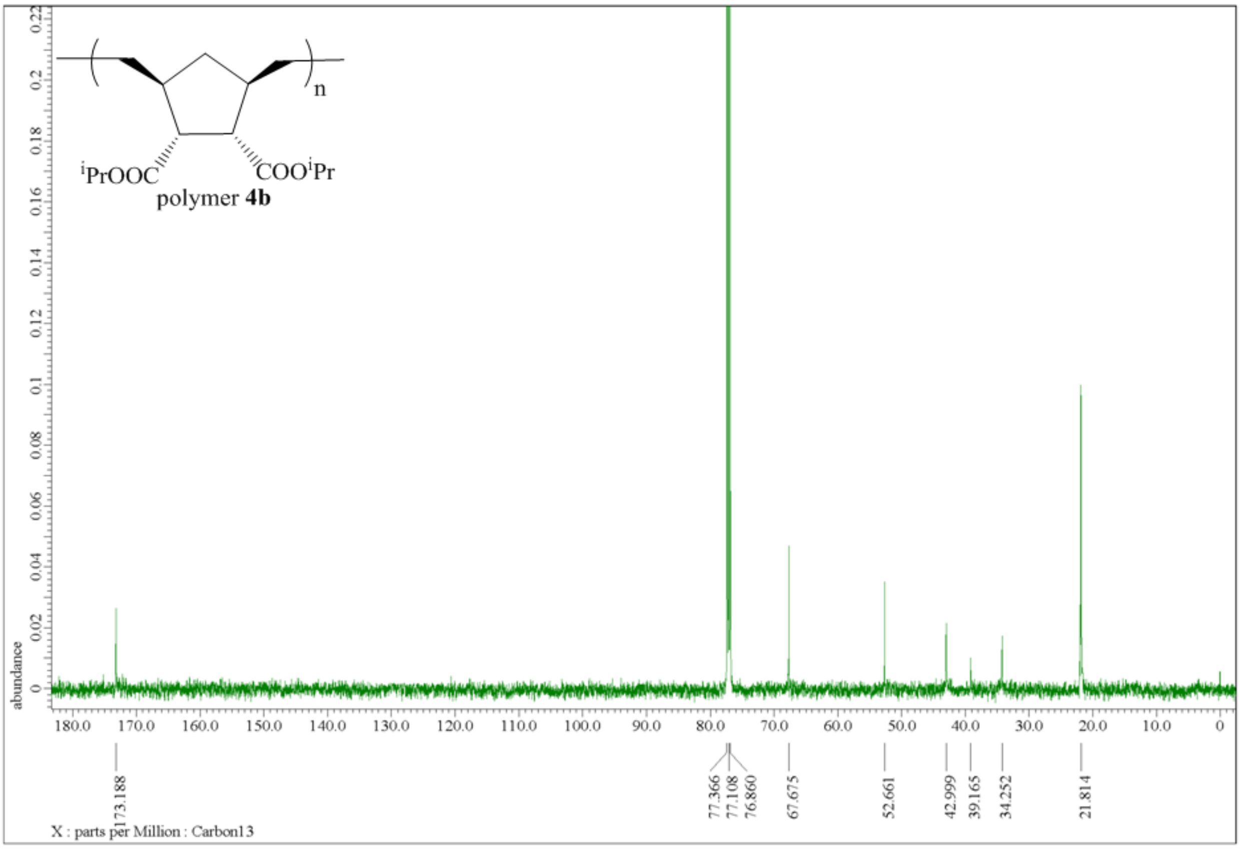

iPrOOC
COOiPr
n
polymer 4b
abundance
X : parts per Million : Carbon13
173.188
77.366
77.108
76.860
67.675
52.661
42.999
39.165
34.252
21.814

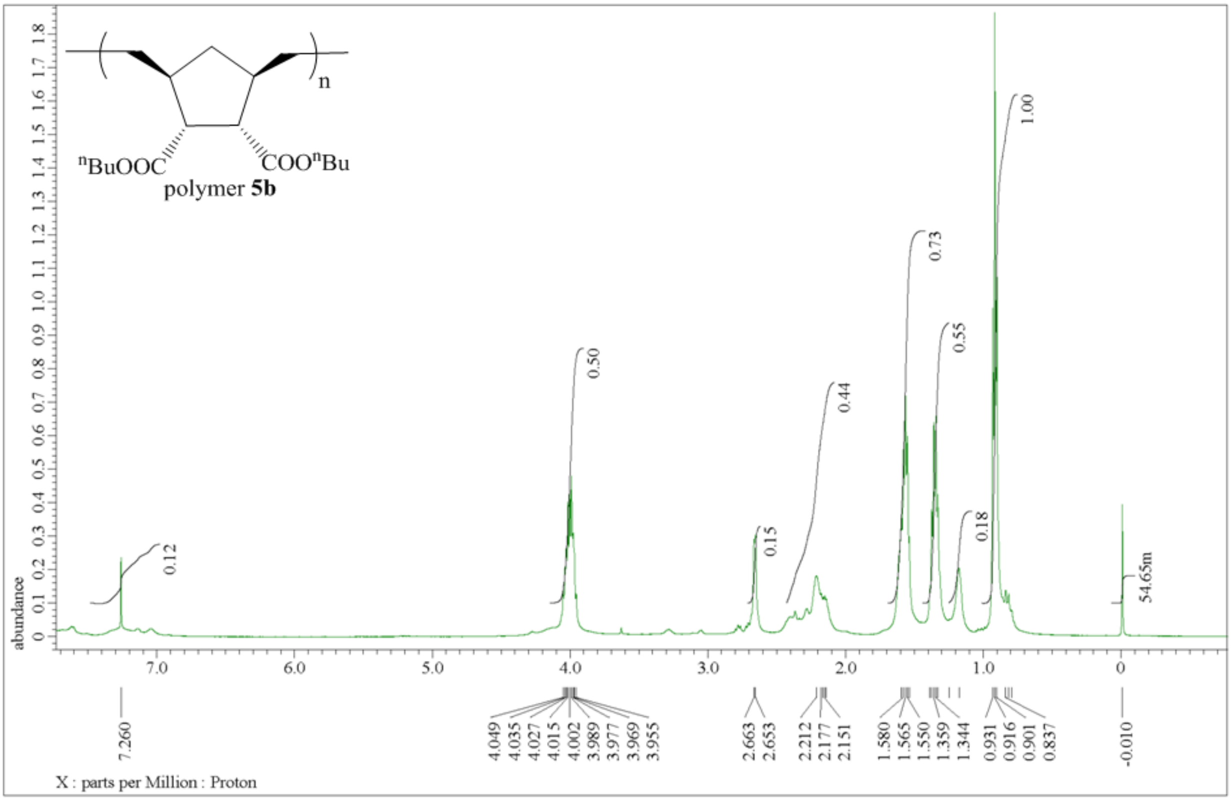

ⁿBuOOC
COOⁿBu
n
polymer 5b
abundance
X : parts per Million : Proton
0.12
0.50
0.15
0.44
0.73
0.55
0.18
1.00
54.65m
7.260
4.049
4.035
4.027
4.015
4.002
3.989
3.977
3.969
3.955
2.663
2.653
2.212
2.177
2.151
1.580
1.565
1.550
1.359
1.344
0.931
0.916
0.901
0.837
-0.010

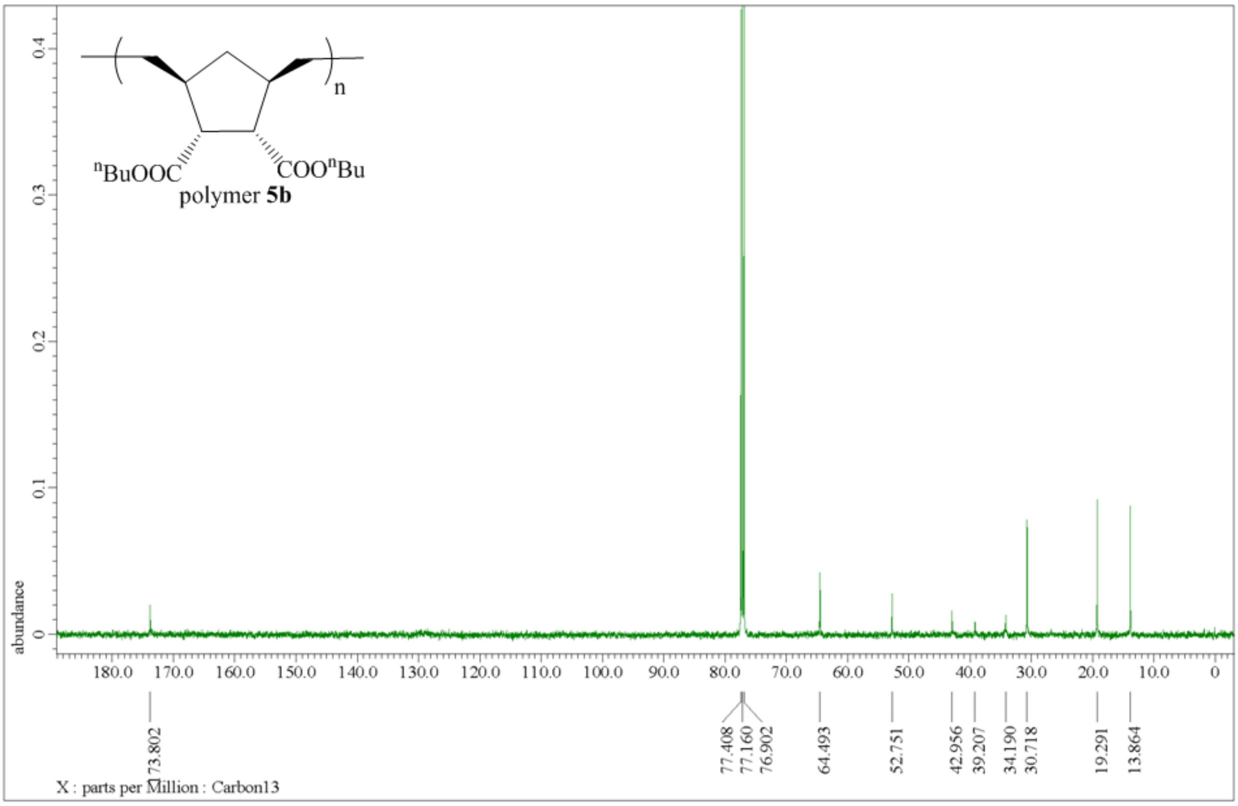

ⁿBuOOC
COOⁿBu
n
polymer 5b
abundance
X : parts per Million : Carbon13
173.802
77.408
77.160
76.902
64.493
52.751
42.956
39.207
34.190
30.718
19.291
13.864

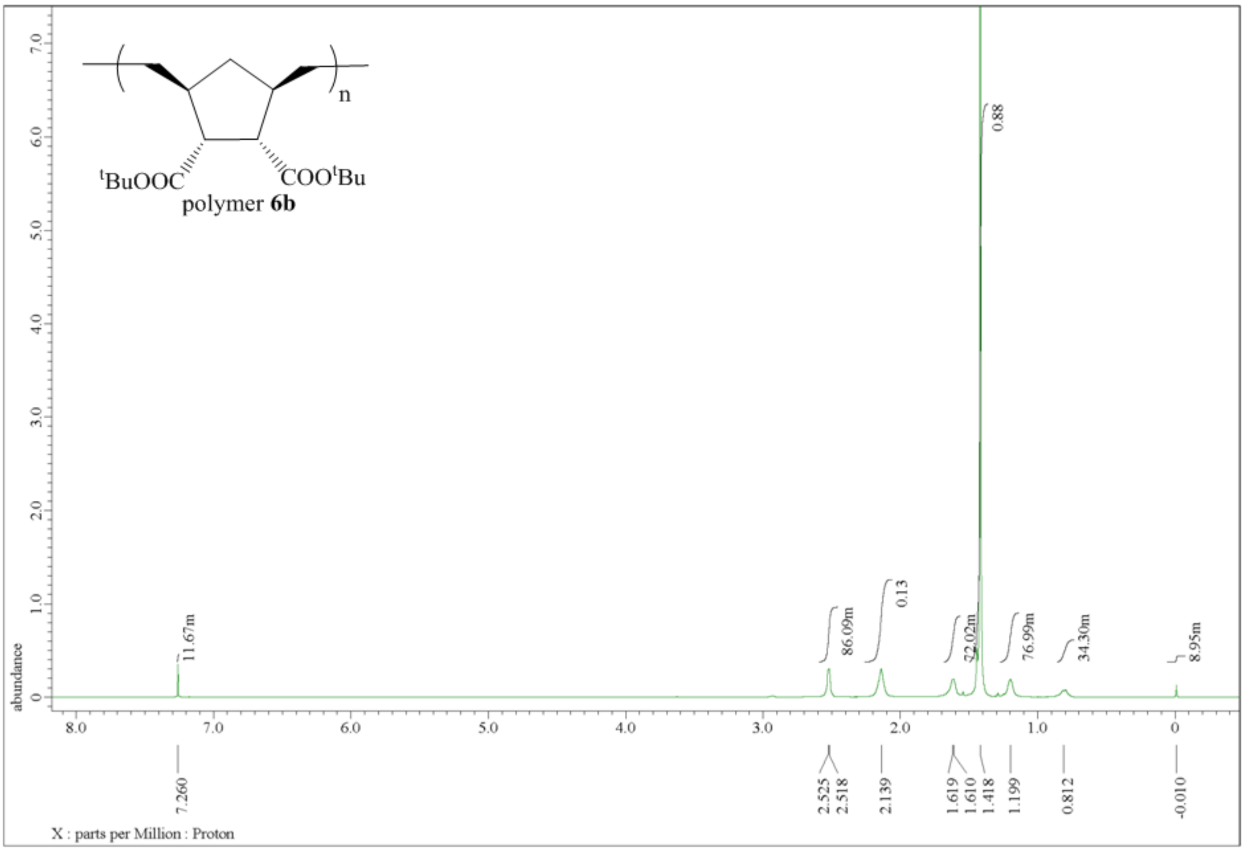

polymer **6b**
^{t}BuOOC
COO^{t}Bu
abundance
X : parts per Million : Proton
7.260
2.525
2.518
2.139
1.619
1.610
1.418
1.199
0.812
-0.010
11.67m
86.09m
0.13
22.02m
0.88
76.99m
34.30m
8.95m

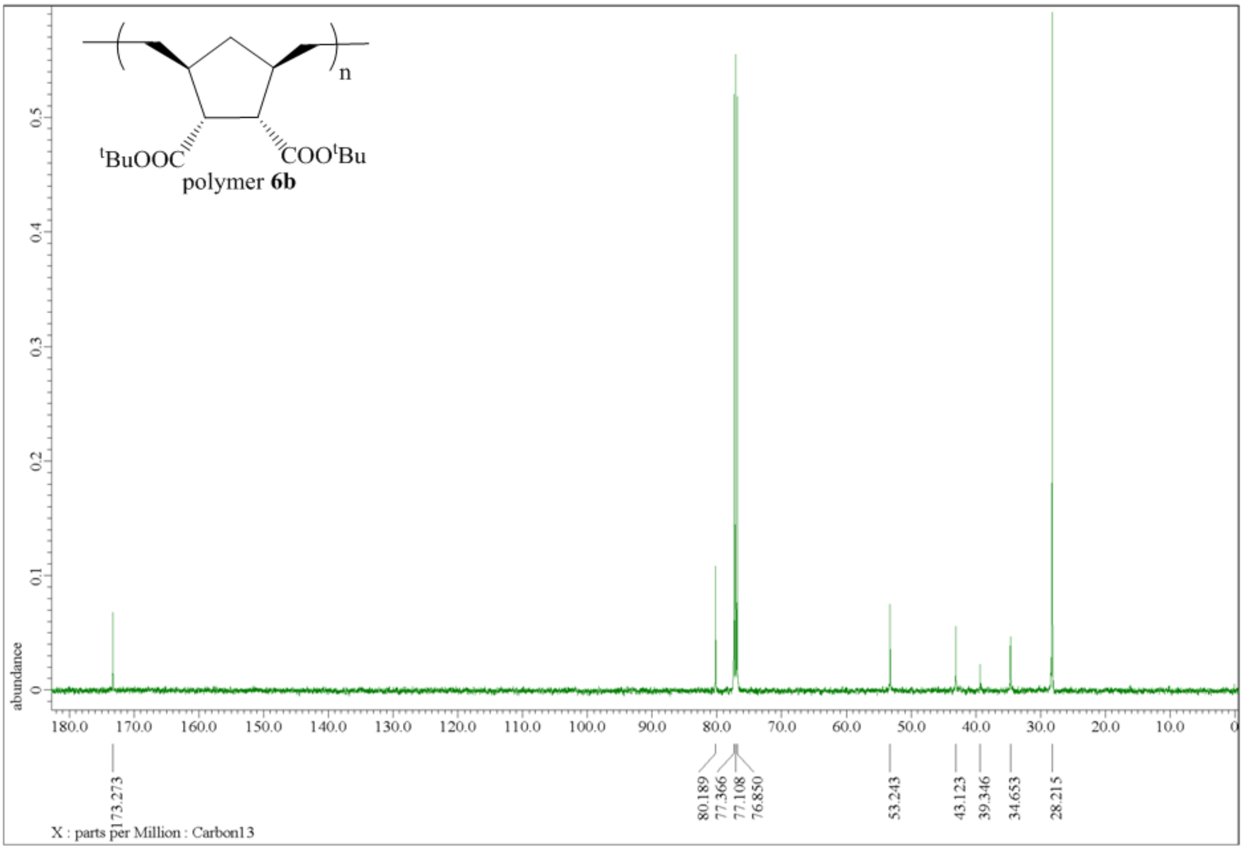

polymer **6b**
^{t}BuOOC
COO^{t}Bu
abundance
X : parts per Million : Carbon13
173.273
80.189
77.366
77.108
76.850
53.243
43.123
39.346
34.653
28.215

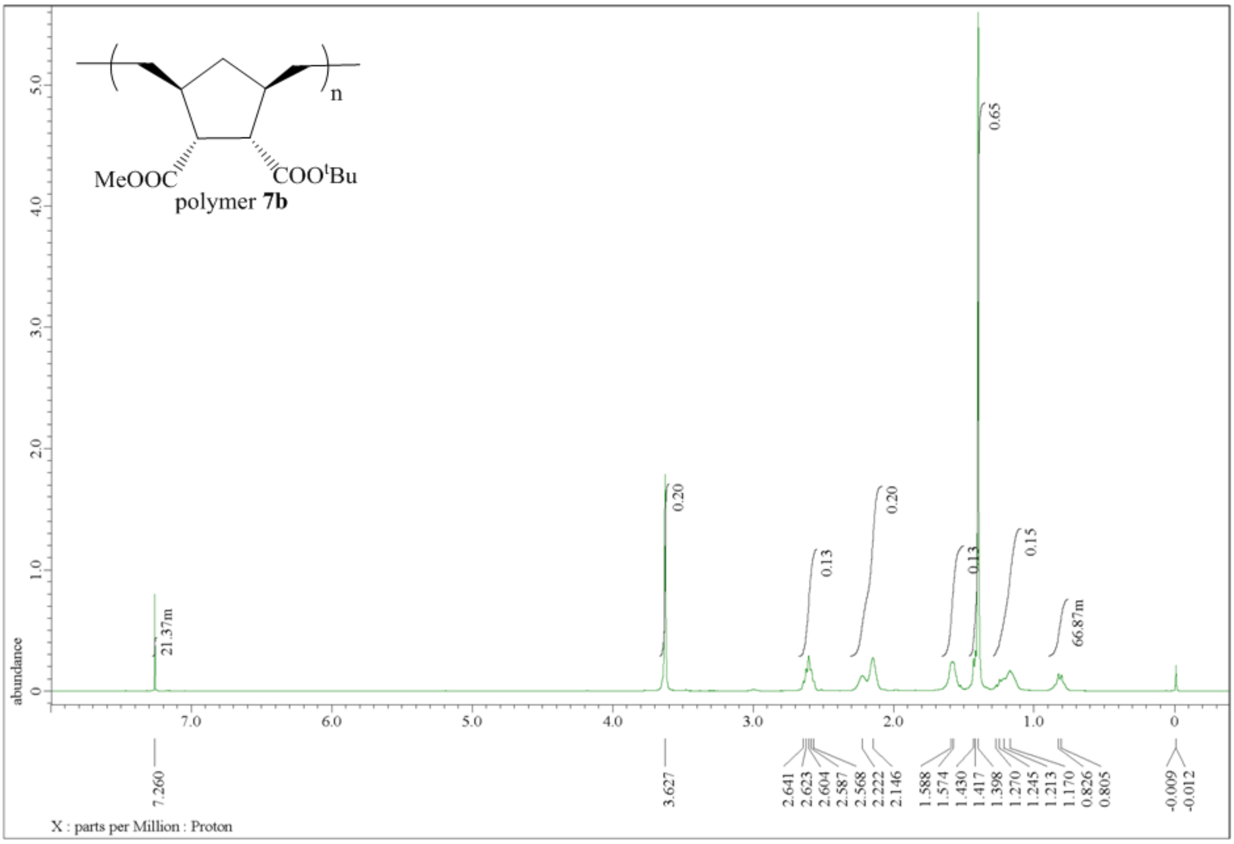
MeOOC
COO^{t}Bu
polymer 7b
n
abundance
21.37m
0.20
0.13
0.20
0.13
0.65
0.15
66.87m
7.260
3.627
2.641
2.623
2.604
2.587
2.568
2.222
2.146
1.588
1.574
1.430
1.417
1.398
1.270
1.245
1.213
1.170
0.826
0.805
-0.009
-0.012
X : parts per Million : Proton

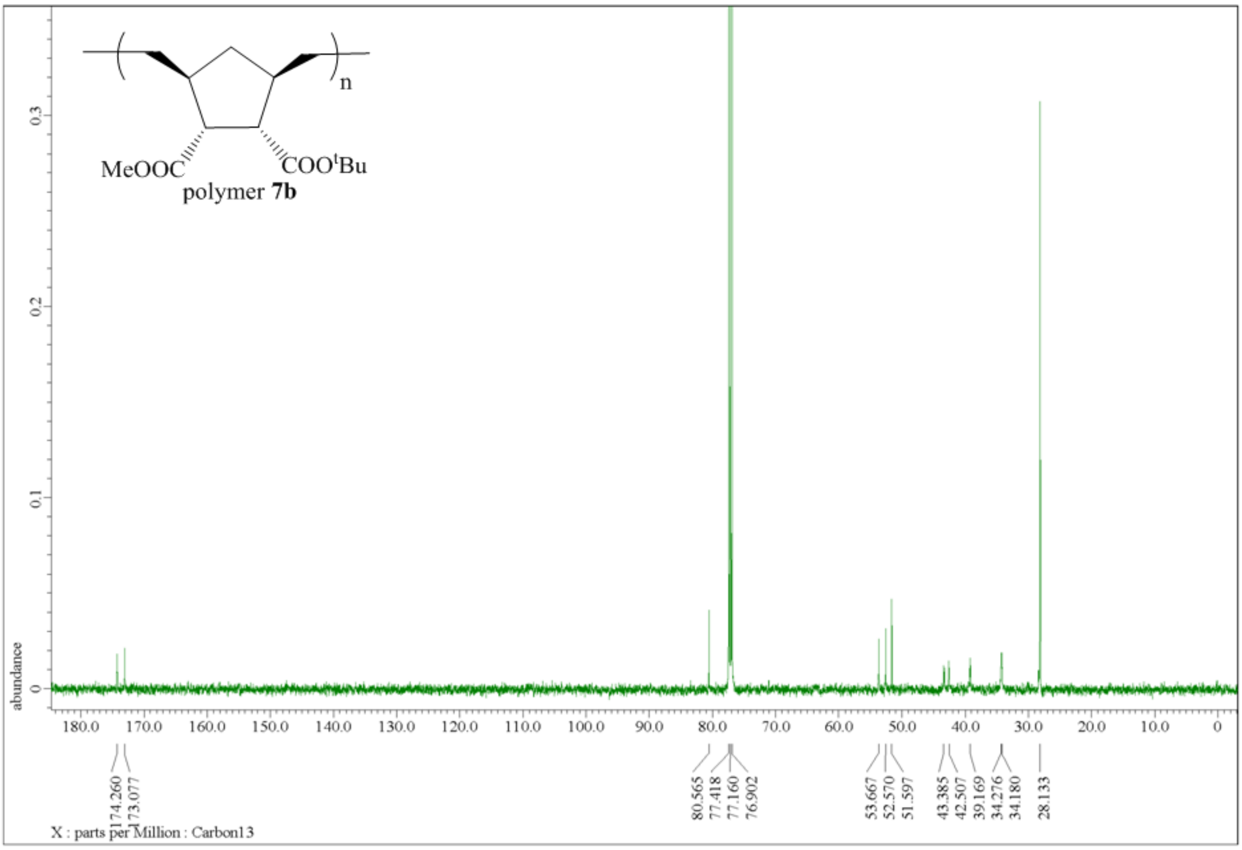
MeOOC
COO^{t}Bu
polymer 7b
n
abundance
174.260
173.077
80.565
77.418
77.160
76.902
53.667
52.570
51.597
43.385
42.507
39.169
34.276
34.180
28.133
X : parts per Million : Carbon13

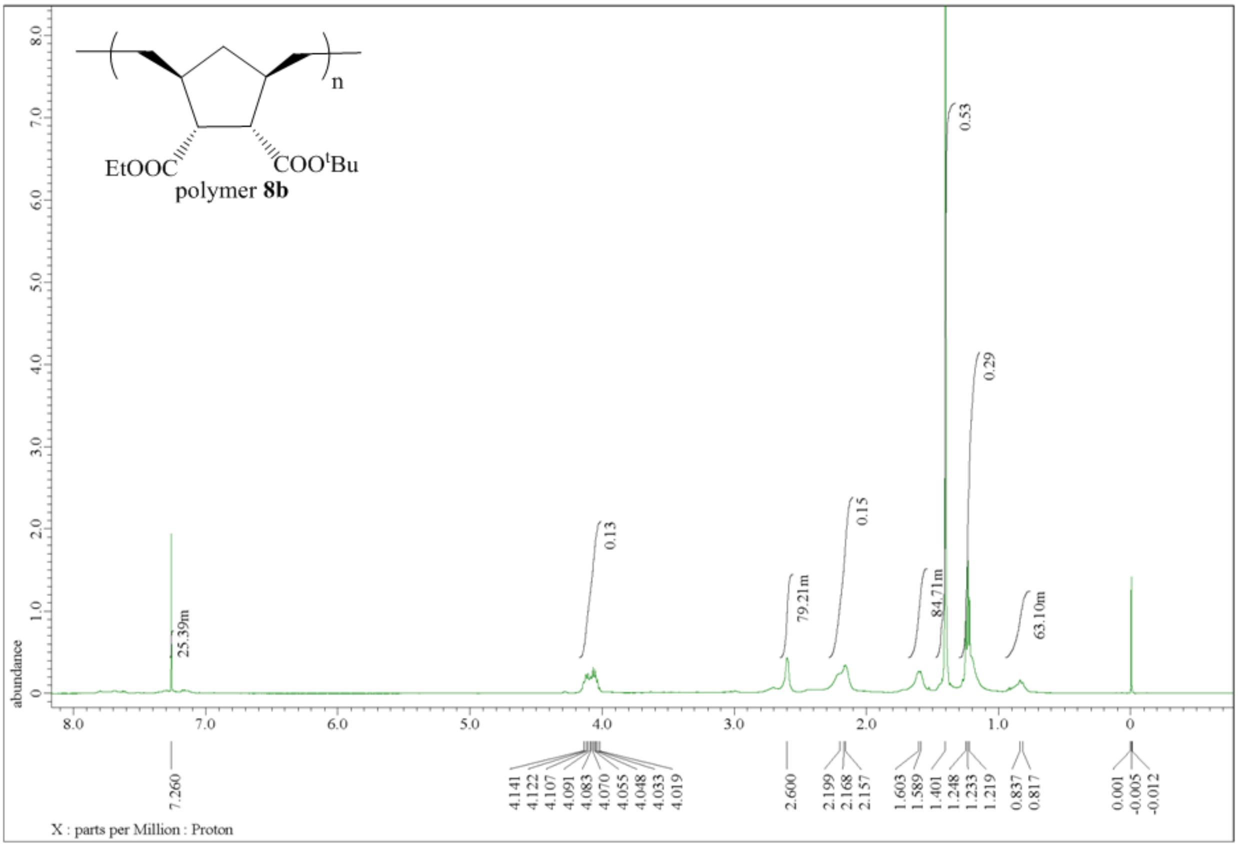
polymer **8b**
EtOOC
COO^{t}Bu
X : parts per Million : Proton
abundance

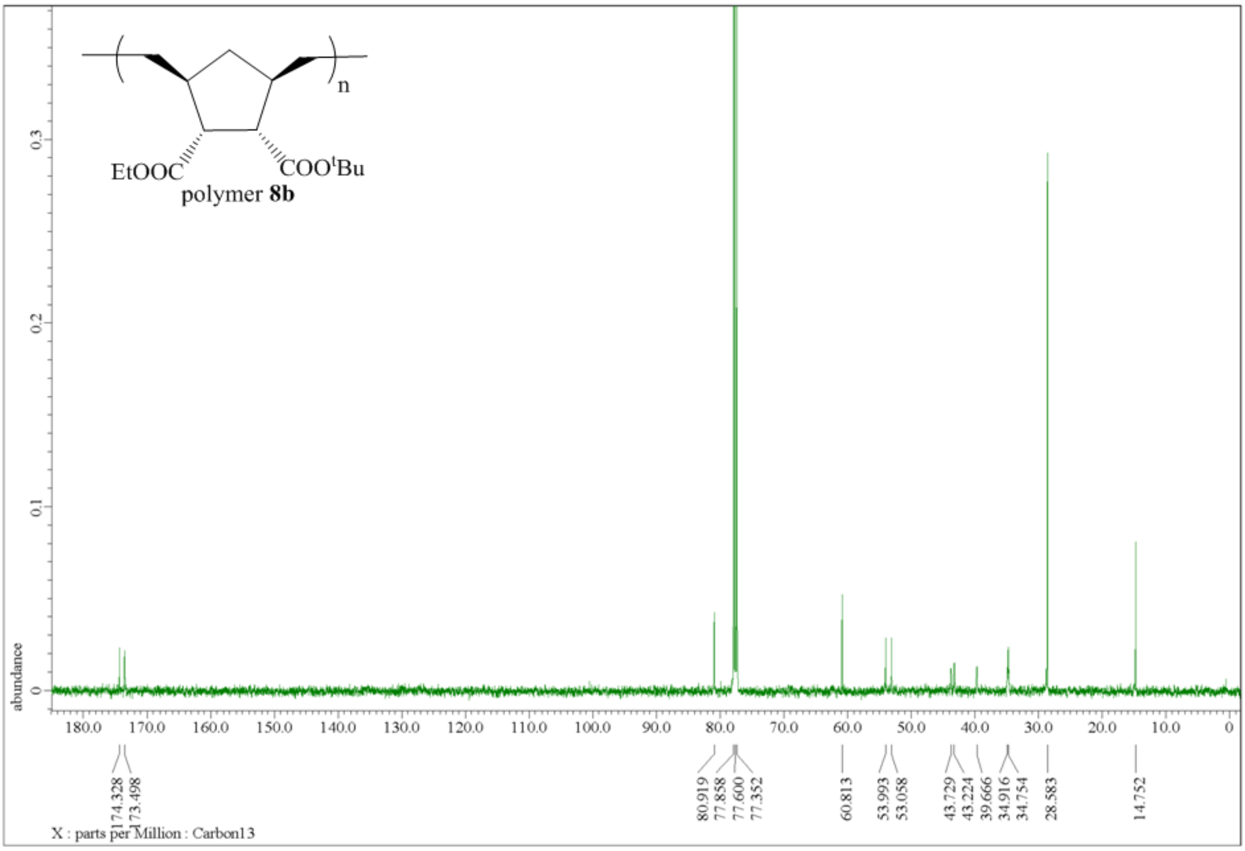
polymer **8b**
EtOOC
COO^{t}Bu
X : parts per Million : Carbon13
abundance

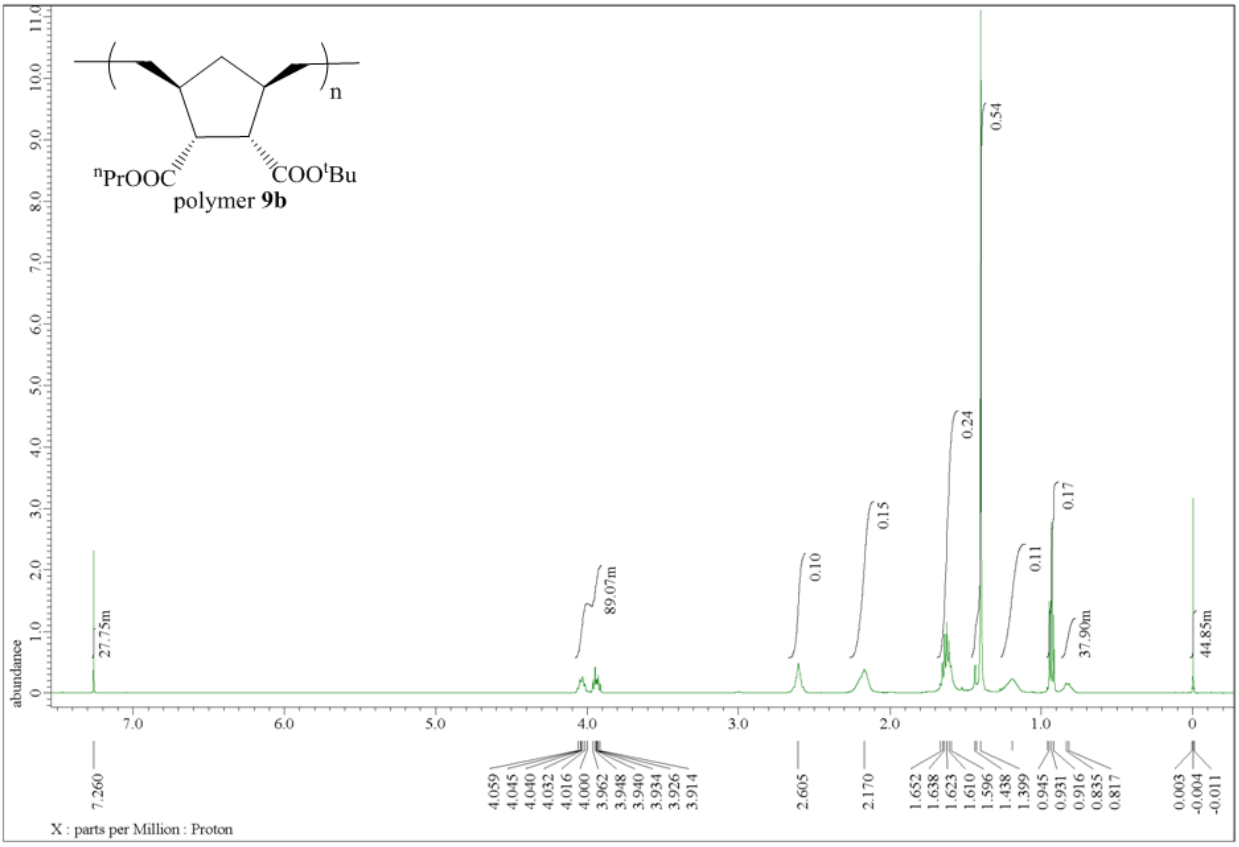

polymer **9b**
^{n}PrOOC
COO^{t}Bu
n
abundance
X : parts per Million : Proton
7.260
4.059
4.045
4.040
4.032
4.016
4.000
3.962
3.948
3.940
3.934
3.926
3.914
2.605
2.170
1.652
1.638
1.623
1.610
1.596
1.438
1.399
0.945
0.931
0.916
0.835
0.817
0.003
-0.004
-0.011
27.75m
89.07m
0.10
0.15
0.24
0.54
0.11
0.17
37.90m
44.85m

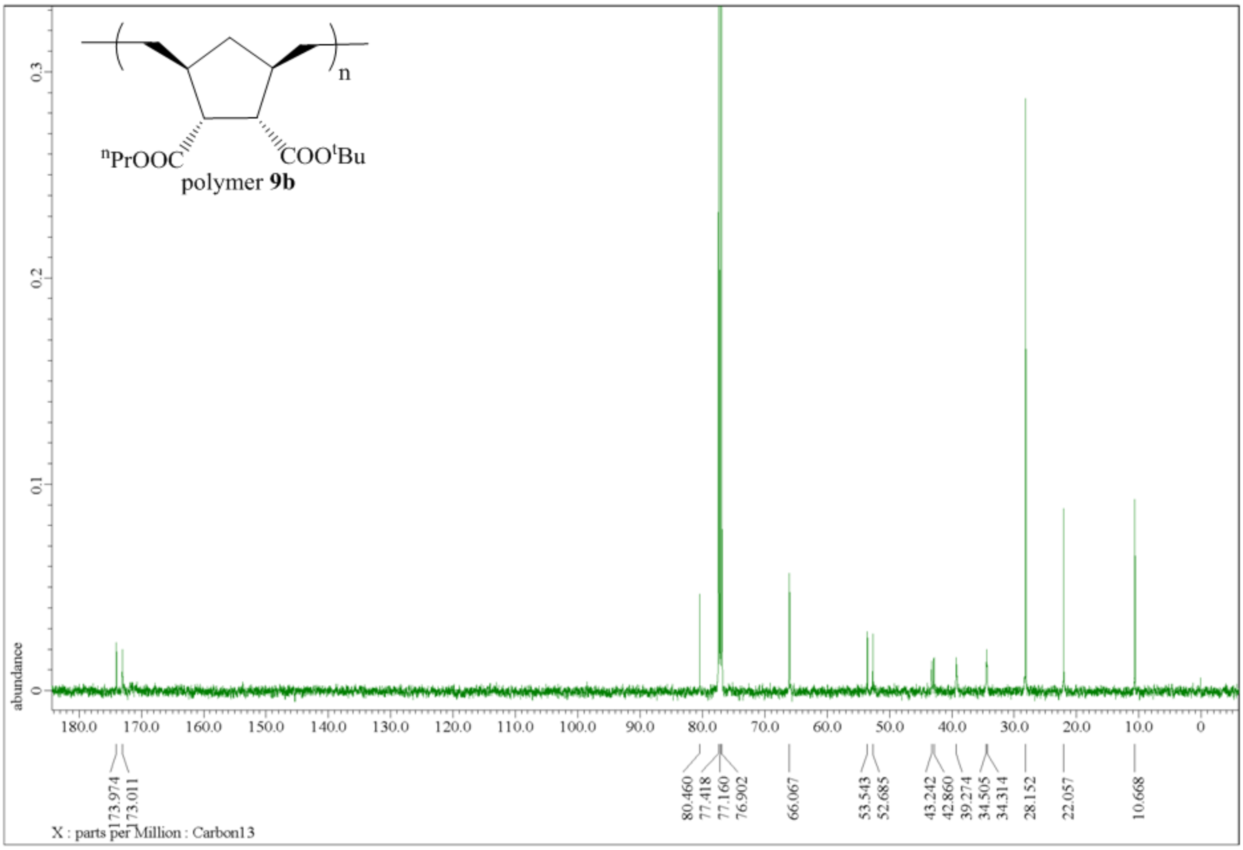

polymer **9b**
^{n}PrOOC
COO^{t}Bu
n
abundance
X : parts per Million : Carbon13
173.974
173.011
80.460
77.418
77.160
76.902
66.067
53.543
52.685
43.242
42.860
39.274
34.505
34.314
28.152
22.057
10.668

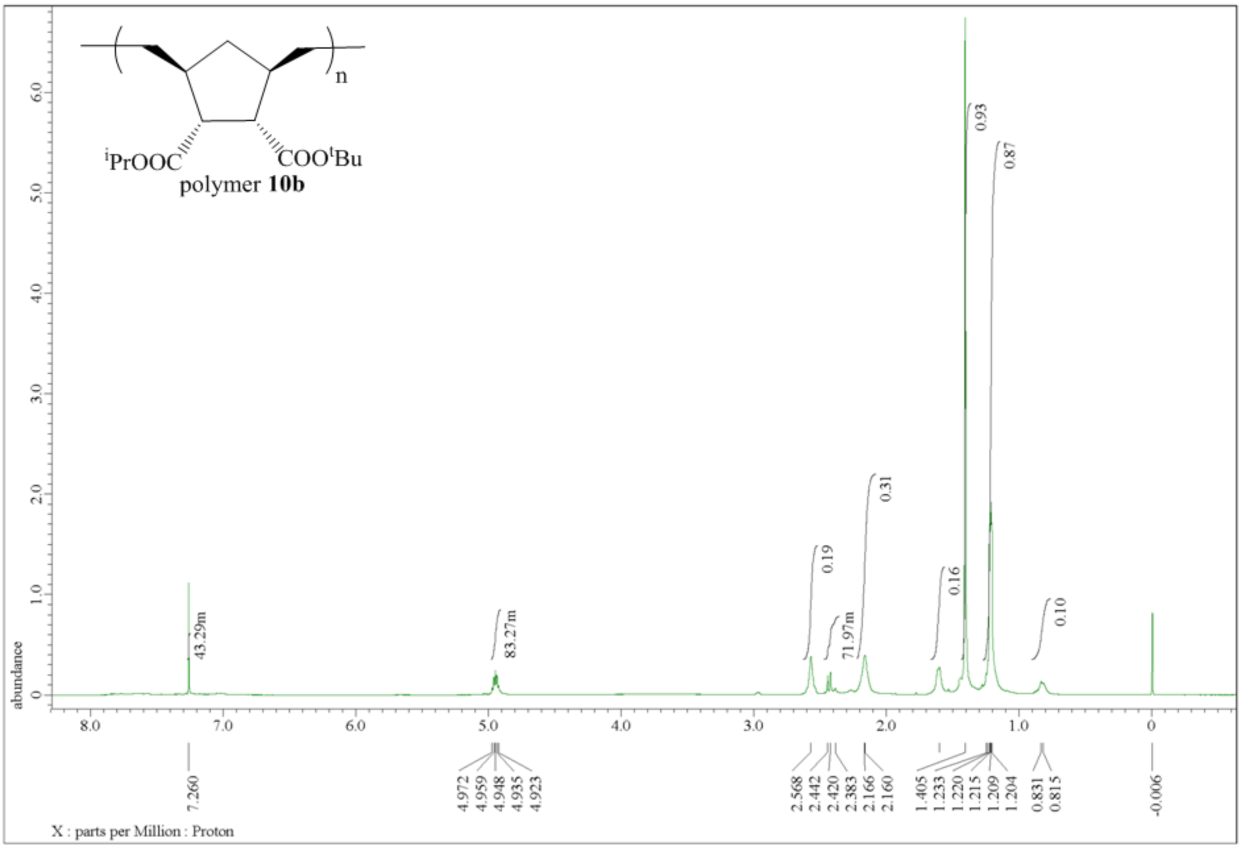
^{i}PrOOC
COO^{t}Bu
n
polymer **10b**
abundance
43.29m
83.27m
0.19
71.97m
0.31
0.16
0.93
0.87
0.10
7.260
4.972
4.959
4.948
4.935
4.923
2.568
2.442
2.420
2.383
2.166
2.160
1.405
1.233
1.220
1.215
1.209
1.204
0.831
0.815
-0.006
X : parts per Million : Proton

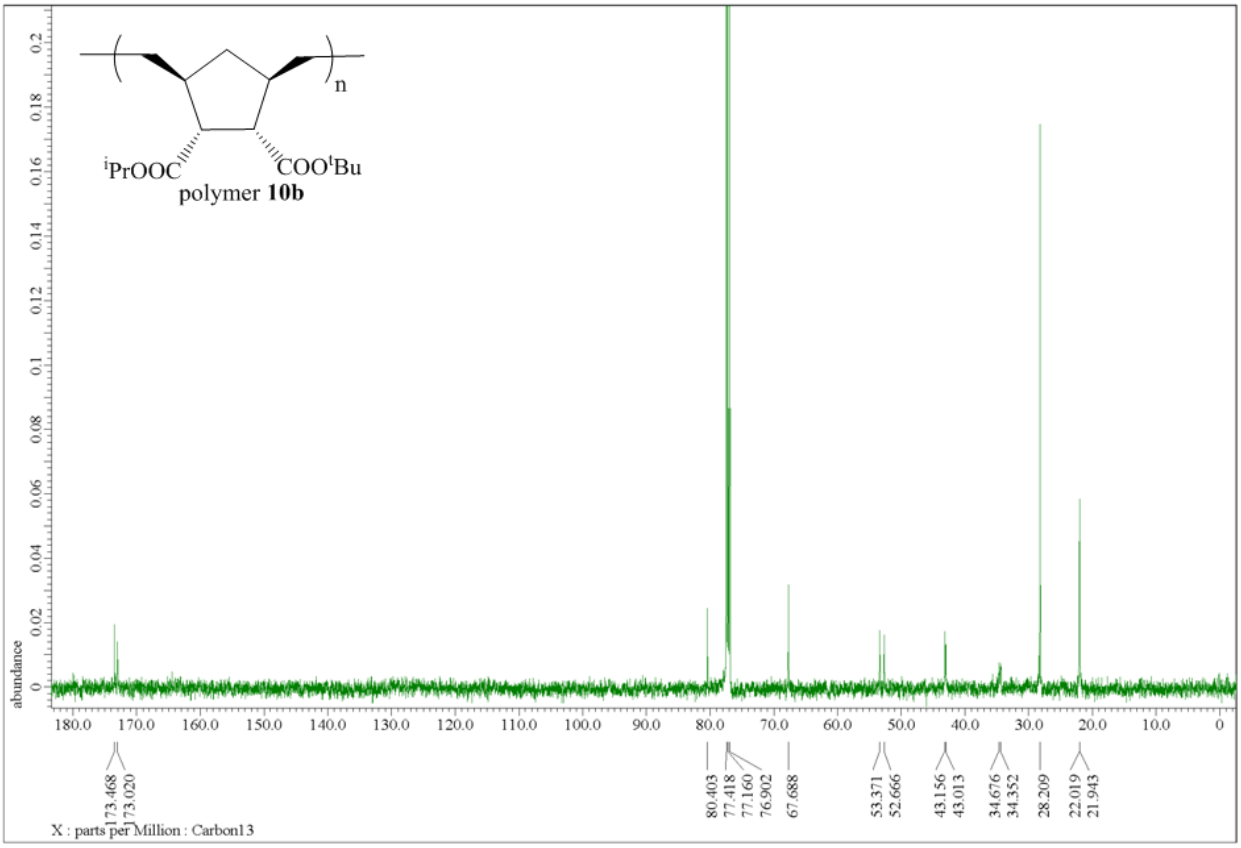
^{i}PrOOC
COO^{t}Bu
n
polymer **10b**
abundance
173.468
173.020
80.403
77.418
77.160
76.902
67.688
53.371
52.666
43.156
43.013
34.676
34.352
28.209
22.019
21.943
X : parts per Million : Carbon13

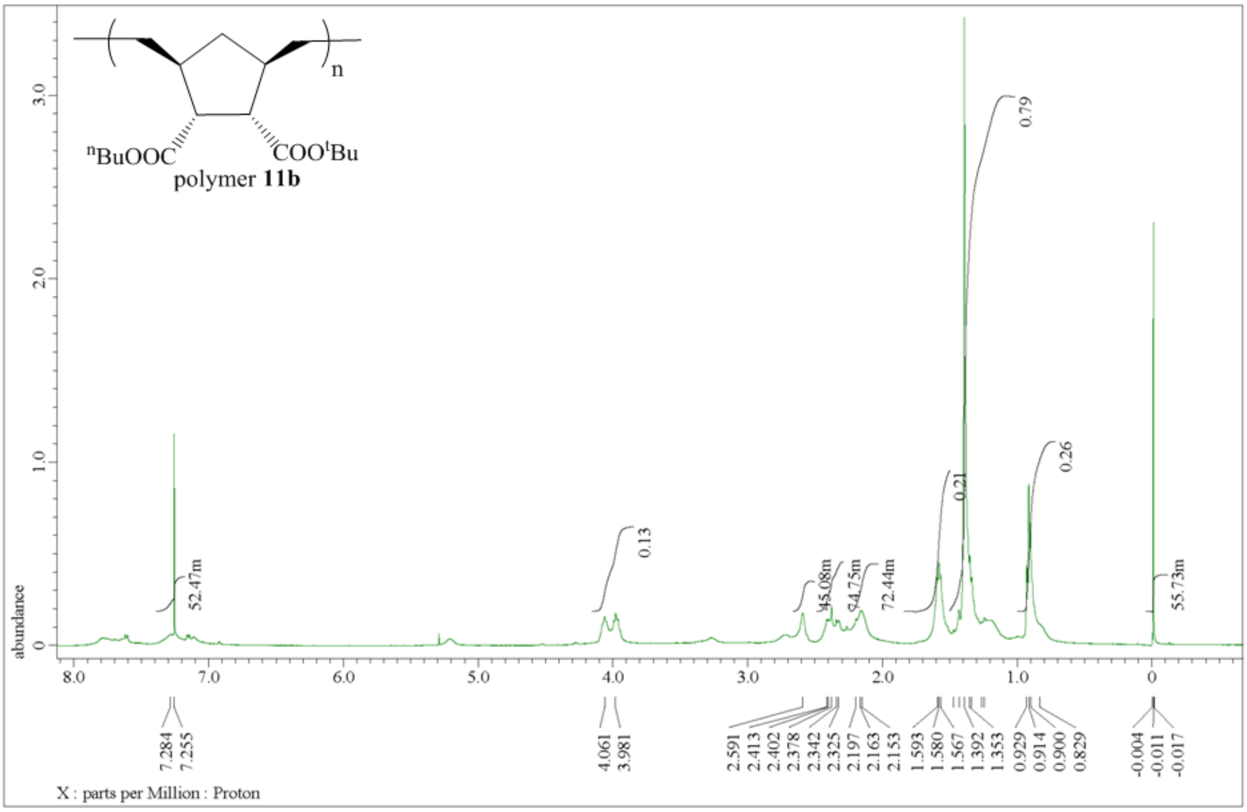

^{n}BuOOC
COO^{t}Bu
polymer **11b**
abundance
X : parts per Million : Proton

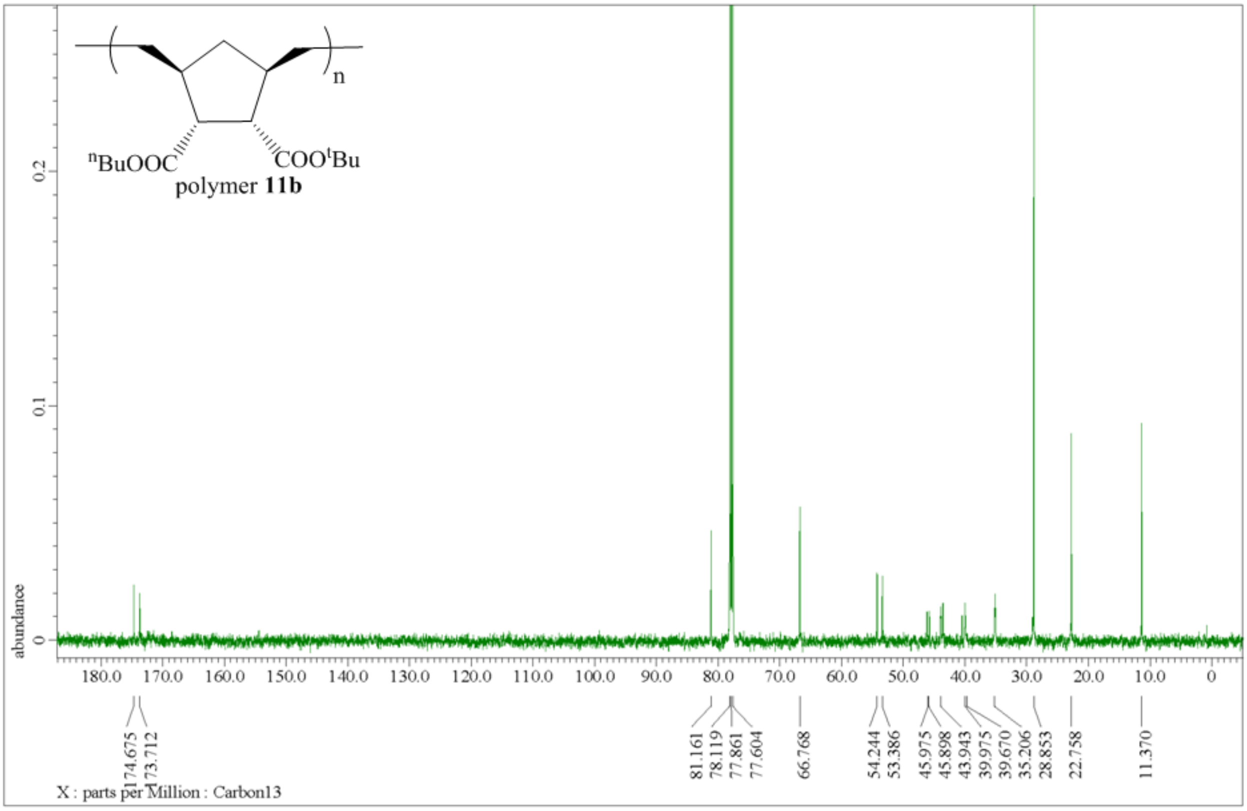

^{n}BuOOC
COO^{t}Bu
polymer **11b**
abundance
X : parts per Million : Carbon13

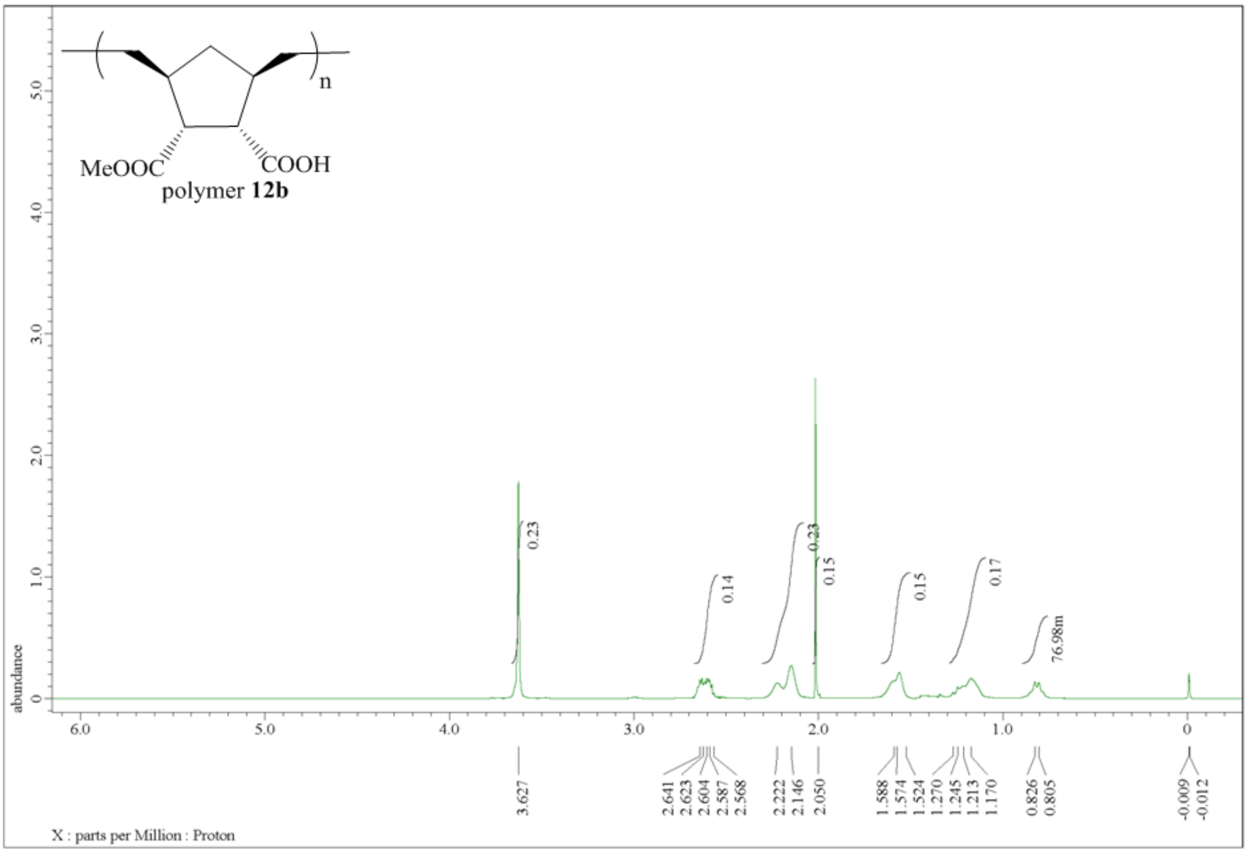
MeOOC
COOH
n
polymer 12b
abundance
0.23
0.14
0.23
0.15
0.15
0.17
76.98m
3.627
2.641
2.623
2.604
2.587
2.568
2.222
2.146
2.050
1.588
1.574
1.524
1.270
1.245
1.213
1.170
0.826
0.805
-0.009
-0.012
X : parts per Million : Proton

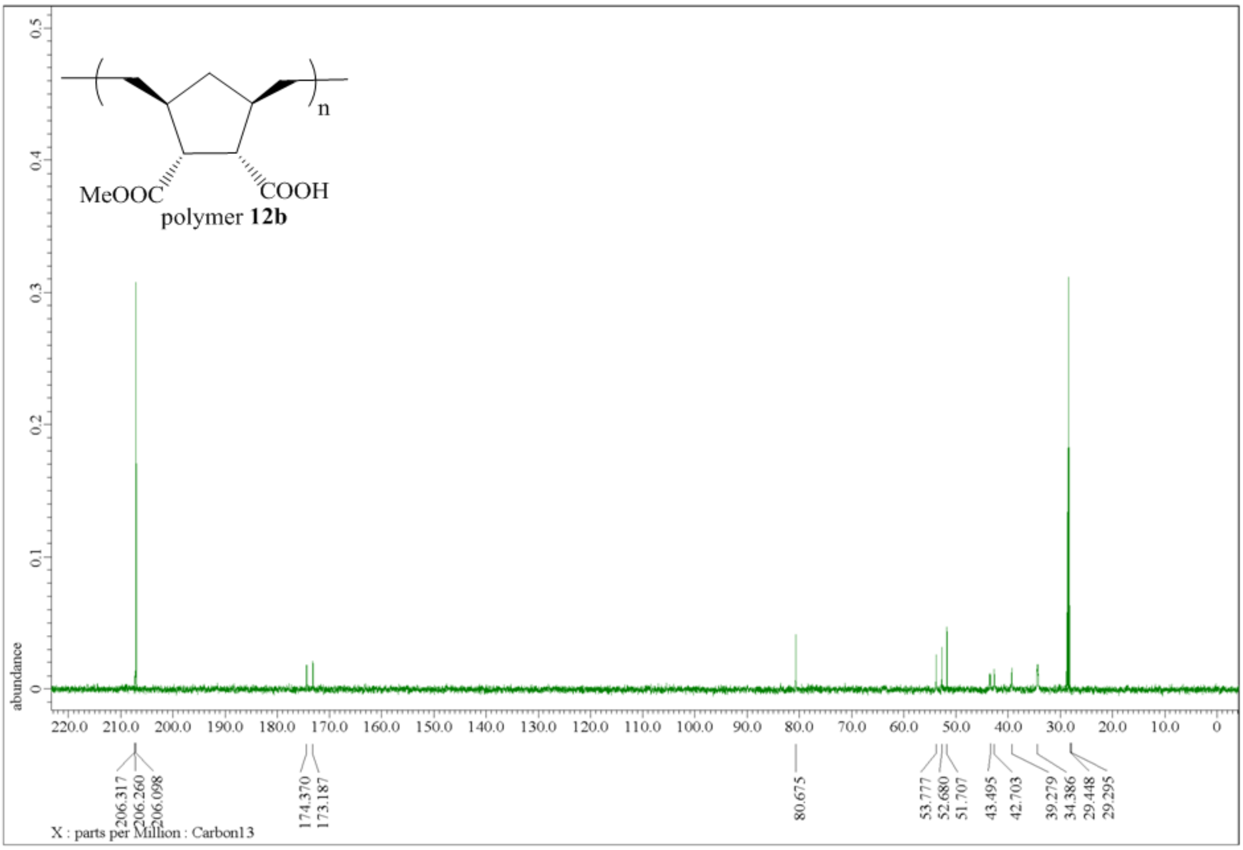
MeOOC
COOH
n
polymer 12b
abundance
206.317
206.260
206.098
174.370
173.187
80.675
53.777
52.680
51.707
43.495
42.703
39.279
34.386
29.448
29.295
X : parts per Million : Carbon13

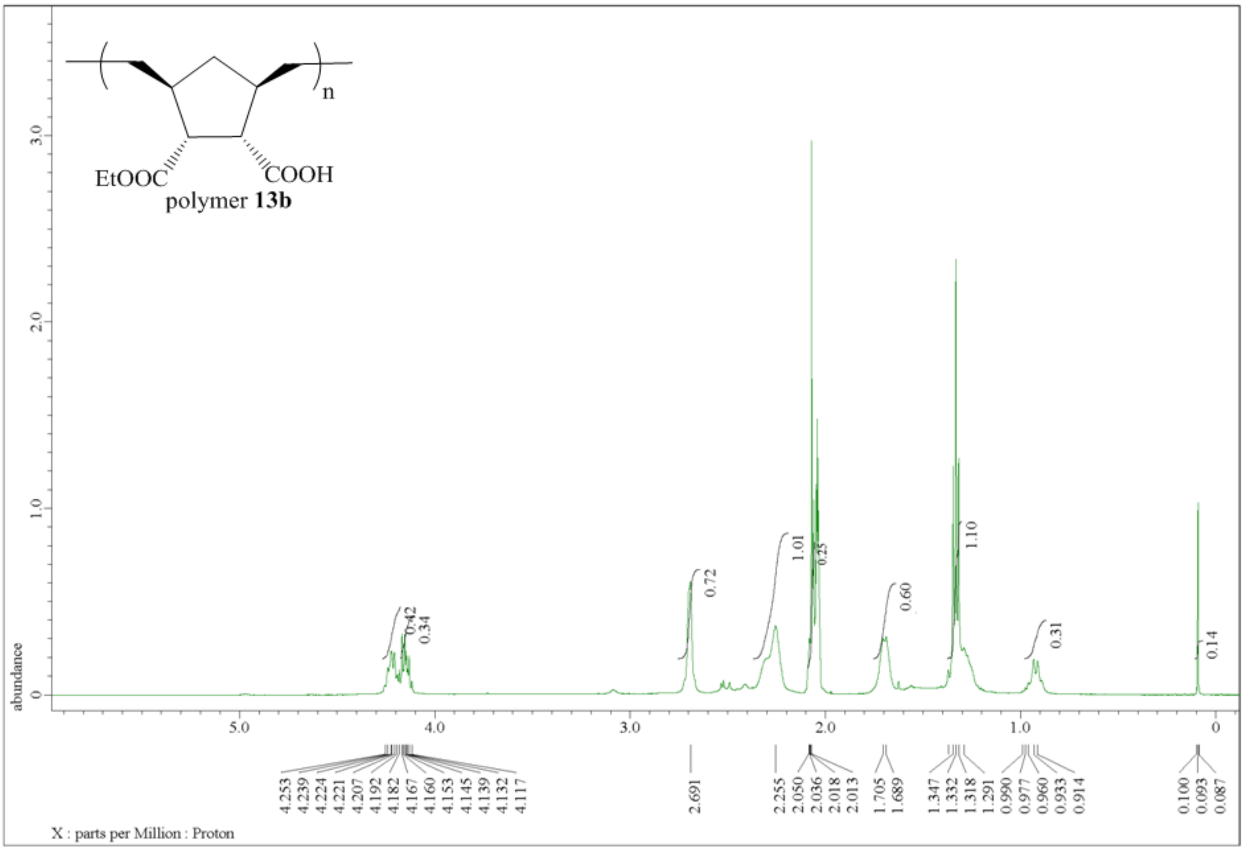
EtOOC
COOH
n
polymer 13b
abundance
X : parts per Million : Proton
4.253 4.239 4.224 4.221 4.207 4.192 4.182 4.167 4.160 4.153 4.145 4.139 4.132 4.117
2.691
2.255 2.050 2.036 2.018 2.013
1.705 1.689
1.347 1.332 1.318 1.291 0.990 0.977 0.960 0.933 0.914
0.100 0.093 0.087

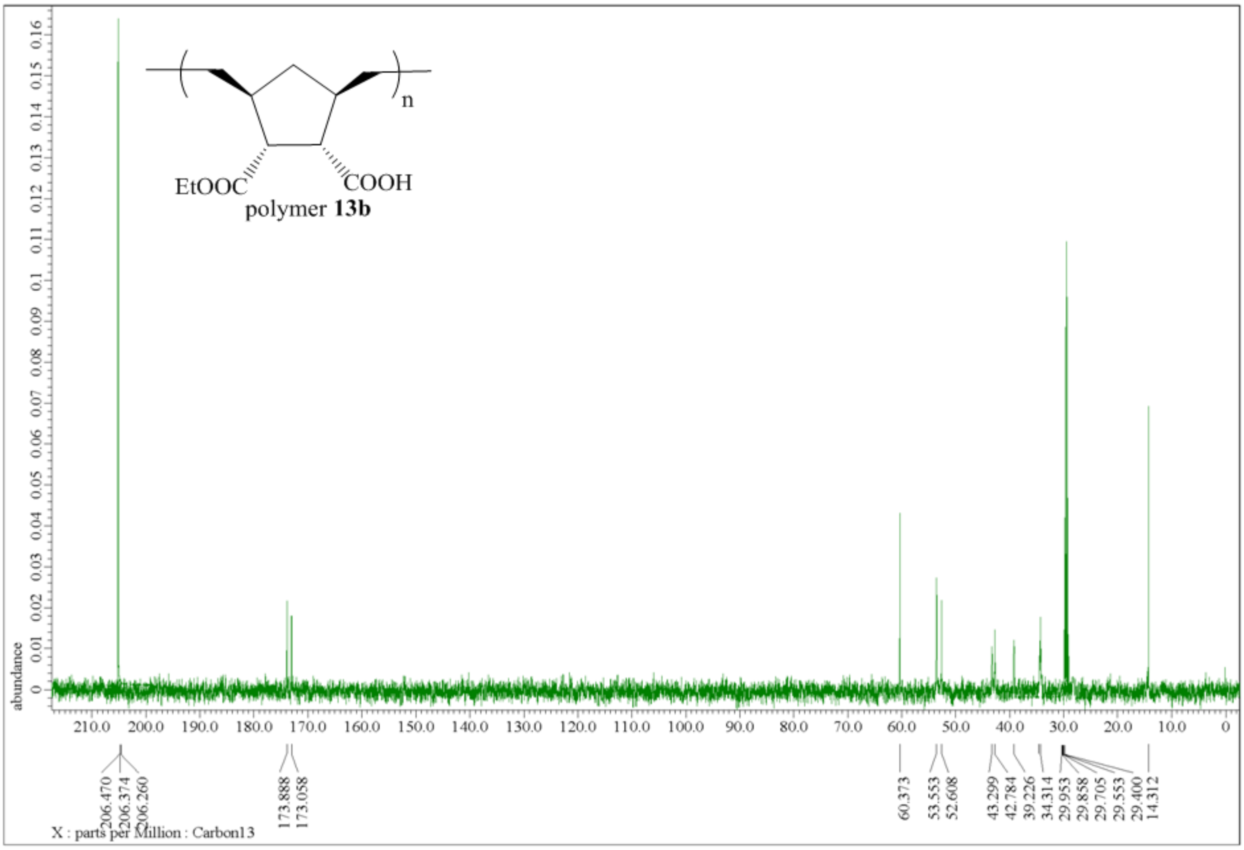
EtOOC
COOH
n
polymer 13b
abundance
X : parts per Million : Carbon13
206.470 206.374 206.260
173.888 173.058
60.373
53.553 52.608
43.299 42.784 39.226 34.314 29.953 29.858 29.705 29.553 29.400 14.312

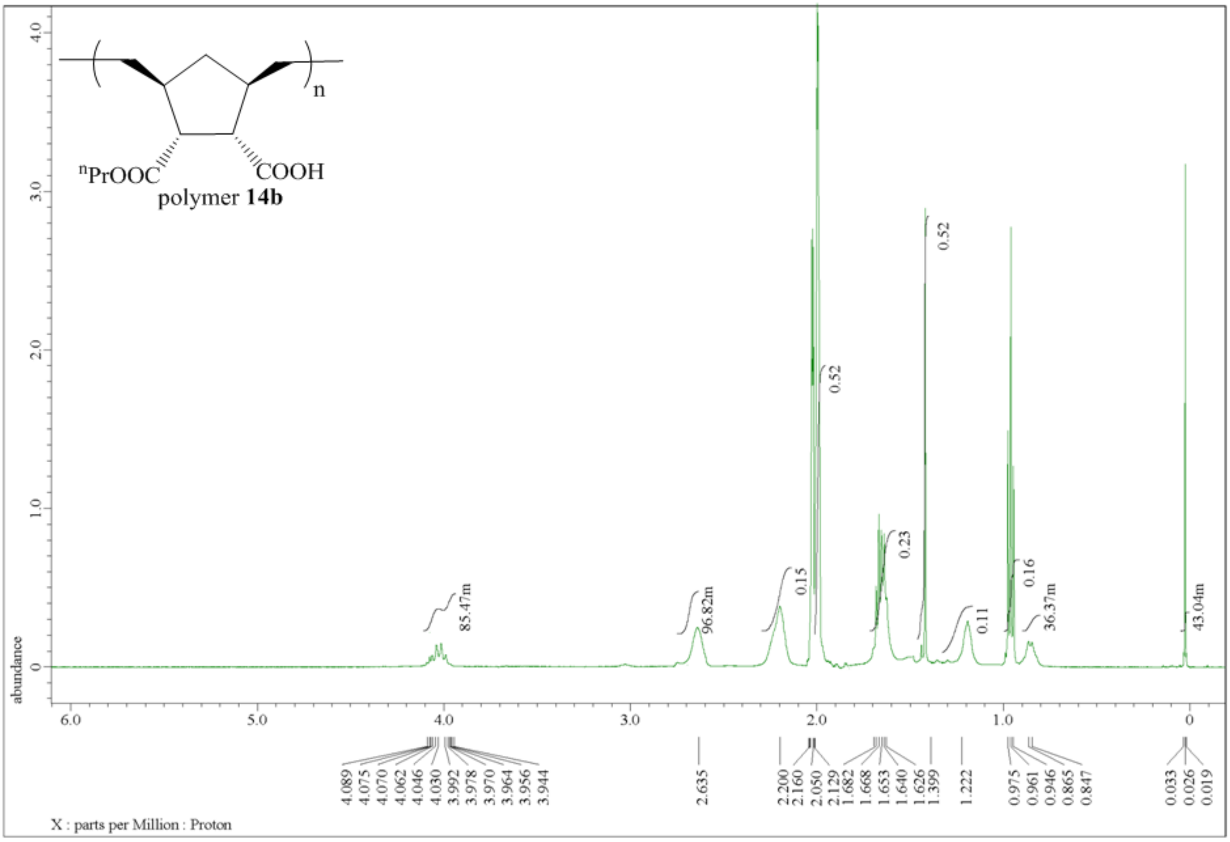

polymer 14b
abundance
X : parts per Million : Proton

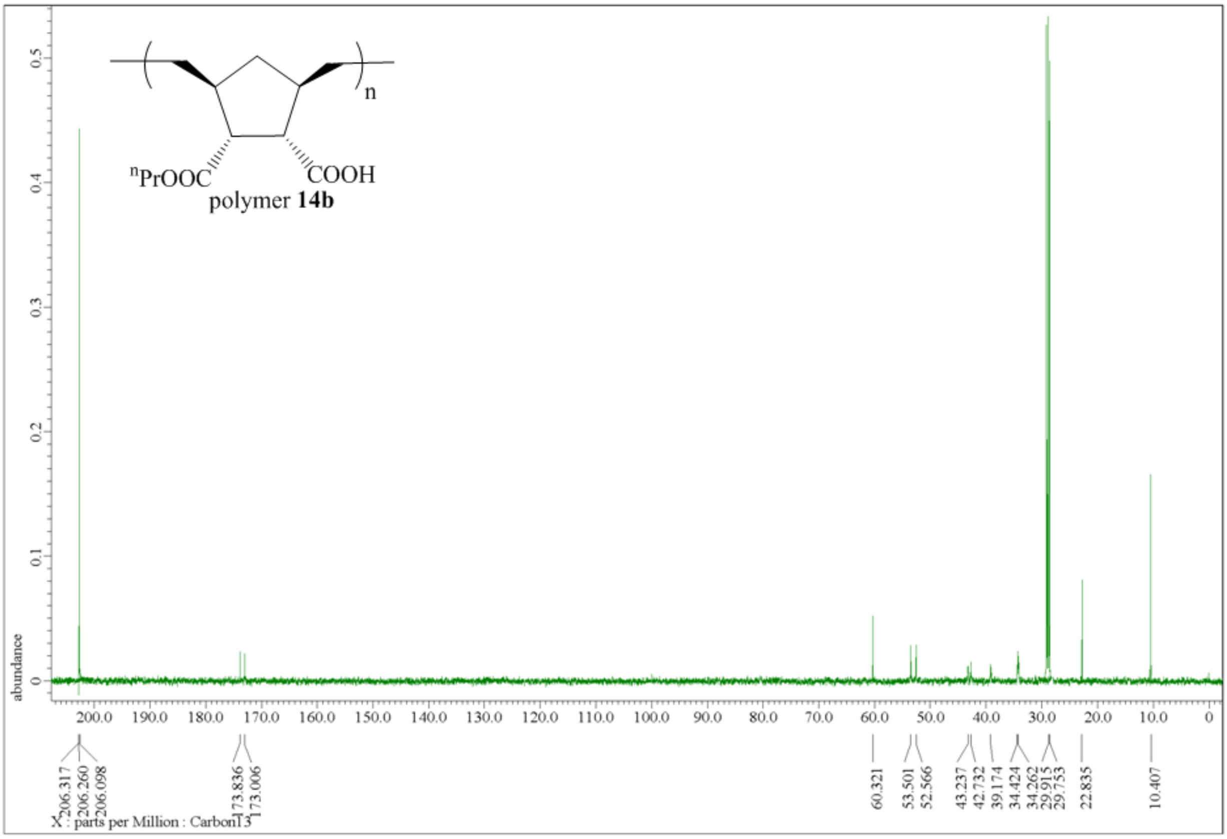

polymer 14b
abundance
X : parts per Million : Carbon13

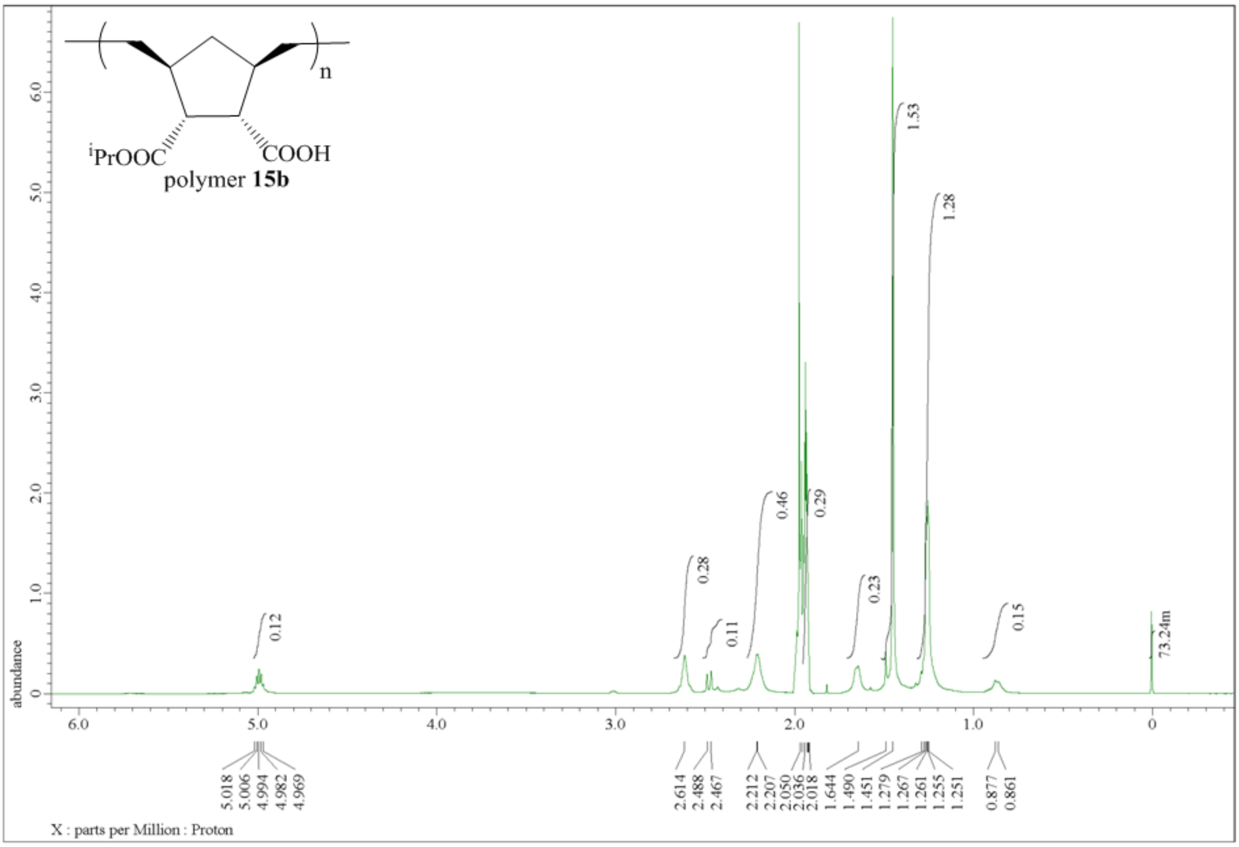
iPrOOC
COOH
polymer 15b
n
abundance
X : parts per Million : Proton
0.12
0.28
0.11
0.46
0.29
0.23
1.53
1.28
0.15
73.24m
5.018
5.006
4.994
4.982
4.969
2.614
2.488
2.467
2.212
2.207
2.050
2.036
2.018
1.644
1.490
1.451
1.279
1.267
1.261
1.255
1.251
0.877
0.861

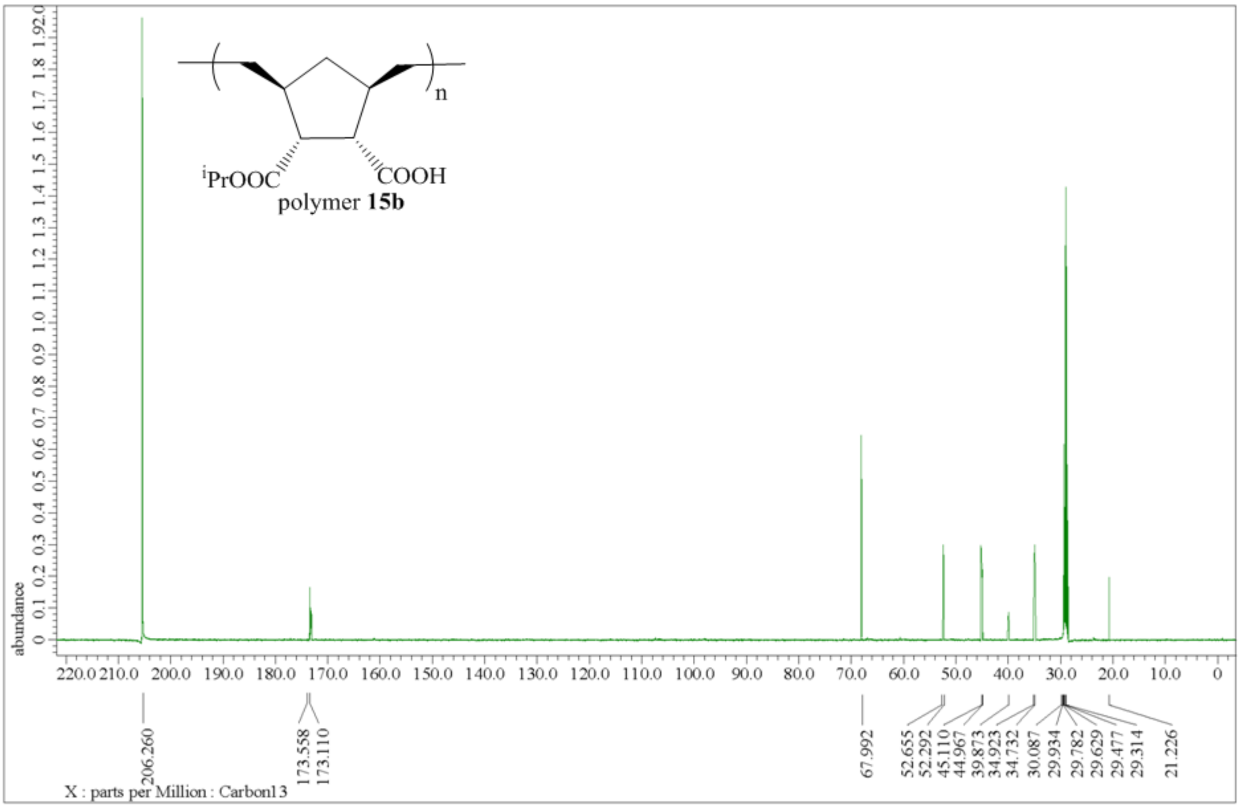
iPrOOC
COOH
polymer 15b
n
abundance
X : parts per Million : Carbon13
206.260
173.558
173.110
67.992
52.655
52.292
45.110
44.967
39.873
34.923
34.732
30.087
29.934
29.782
29.629
29.477
29.314
21.226

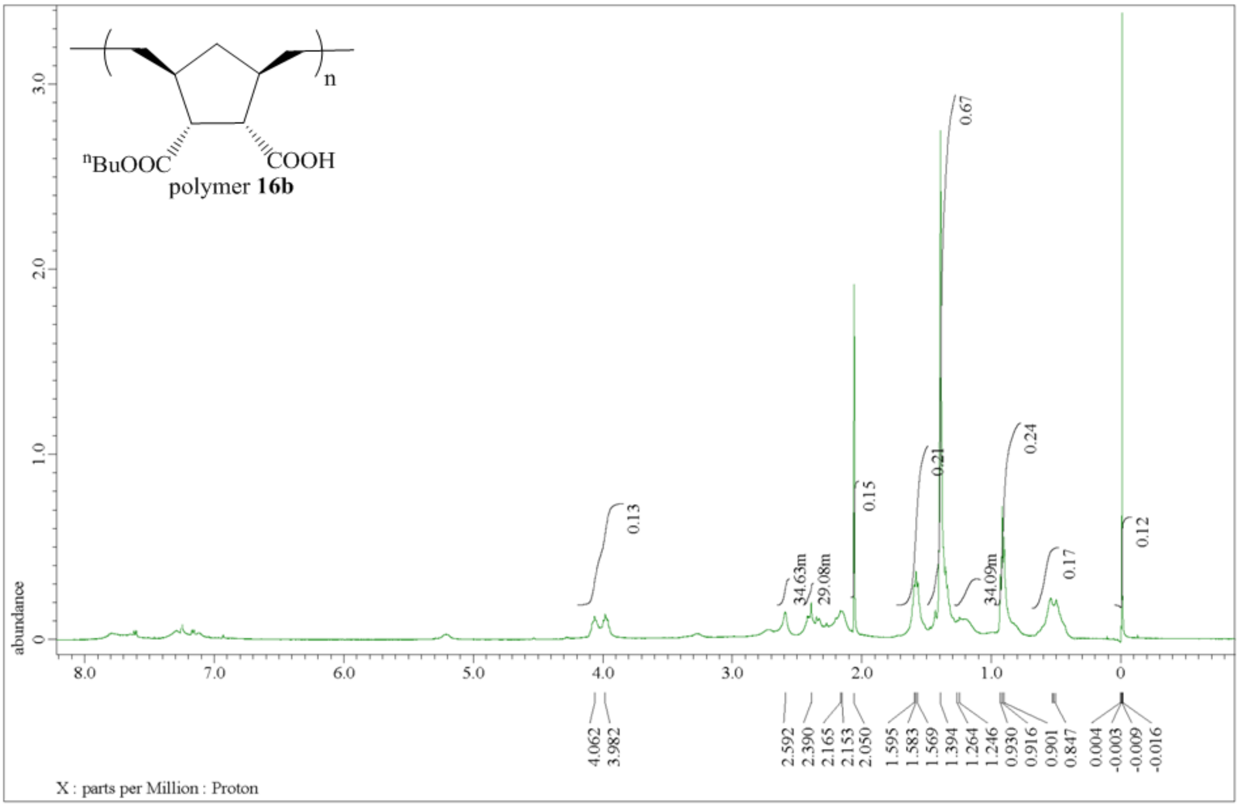

^{n}BuOOC
COOH
polymer **16b**
abundance
X : parts per Million : Proton

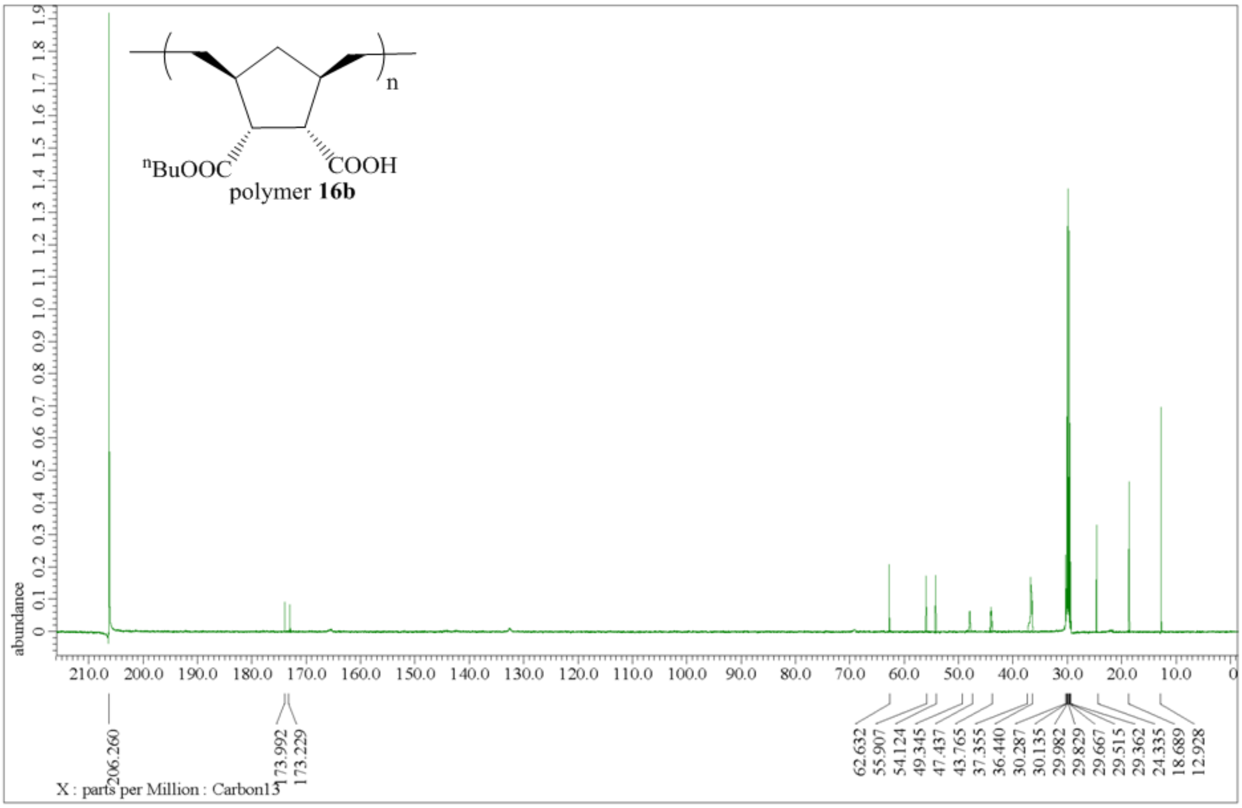

^{n}BuOOC
COOH
polymer **16b**
abundance
X : parts per Million : Carbon13
